# Integrating phylogenetic, phylogeographic, and morphometric analyses to reveal cryptic lineages within the genus *Asaccus* (Reptilia: Squamata: Phyllodactylidae) in Iran

**DOI:** 10.1186/s40850-024-00203-1

**Published:** 2024-06-26

**Authors:** Kamran Kamali, Masoud Nazarizadeh, Faezeh Fatemizadeh, Saeed Salmabadi, Chih–Ming Hung, Mohammad Kaboli

**Affiliations:** 1 Iranian Herpetology Institute, Tehran, Iran; 2 Biodiversity Research Center, Academia Sinica, Taipei, Taiwan; 3 Institute of Parasitology, Biology Centre CAS, České Budějovice, Czech Republic; 4 Faculty of Science, University of South Bohemia, České Budějovice, Czech Republic; 5 Department of Environmental Science, Faculty of Natural Resources, University of Tehran, Tehran, Iran

**Keywords:** Genus *Asaccus*, Leaf-toed geckos, Phylogeny, Phylogeography, Traditional morphology, Speciation, Evolutionary significant units.

## Abstract

The Middle Eastern endemic genus *Asaccus* comprises Southwest Asian leaf-toed geckos. To date, this genus includes 19 species of leaf-toed geckos (seven in Arabia and 12 in the Zagros Mountains). Despite a recent study on the taxonomy and phylogeny of *Asaccus* species in Iran, controversies still remain surrounding the phylogeny and phylogeography of the genus. Here, we used an integrative approach to determine the phylogeny and phylogeography of *Asaccus* species using two mitochondrial genes (12 S and Cyt *b*), and one nuclear gene (*c-mos*). Our results uncovered 22 distinct lineages, demonstrating a significant cryptic diversity that challenges the current morphological classifications of these species. Phylogenetic analyses reinforce the monophyly of the *Asaccus* group, positioning *A. montanus* as a basal lineage, which supports a deep evolutionary divergence dating back to the Late Oligocene, approximately 27.94 million years ago. This genetic diversity also highlights the impact of historical climatic and geographical changes on species diversification. The findings advocate for an integrative approach combining both molecular and morphological data to resolve species identities accurately, thereby enhancing conservation strategies to protect these genetically distinct lineages.

**Supplementary Information:**

The online version contains supplementary material available at 10.1186/s40850-024-00203-1.

## Introduction

The Zagros Mountains are renowned for their exceptional species richness and endemism, serving as a vital biodiversity hotspot in Iran [1, 2]. These mountains emerged from the northeastward movement of the Arabian Plate, which collided with the Eurasian Plate around 10–12.4 million years ago (Mya) [3]. This collision reshaped the terrain and fostered a unique ecological niche, positioning the Zagros as a crucial glacial refuge. The mountain system is divided into northern, central, and southern regions, providing a stable environment that has influenced the speciation patterns of various reptiles. This stability enabled their survival and promoted evolutionary divergence as geographical isolation limited species’ dispersal [4]. The discovery of numerous endemic reptile species, including geckos, highlights the region’s rich biodiversity. Among these finds are several species within the family Phyllodactylidae and others reptiles, emphasizing the Zagros Mountains’ role as a refuge for a wide array of reptile species [5–7].

*Asaccus* (Dixon & Anderson, 1973), a diverse genus within the Phyllodactylidae family (leaf-toed geckos), comprises species predominantly found in the Middle Eastern and South Asian regions. These geckos are exquisitely adapted to arid and semi-arid habitats, featuring cryptic coloration that blends seamlessly with their rocky surroundings and specialized toe pads that enhance their ability to navigate and climb in such challenging environments. One of the key characteristics of *Asaccus* species is the absence of colacal sacs. *Asaccus* species have both nocturnal and diurnal behaviors (some have been recorded active during daytime at the entrance of caves), primarily hunting insects and other small invertebrates under the cover of darkness. Furthermore, these geckos possess unique microstructures on their feet that enable them to adhere firmly to various surfaces, facilitating their remarkable climbing abilities. Their reproductive strategies also reflect adaptations to harsh environments, with some species exhibiting remarkable resilience by laying fewer, but more robust, eggs [5].

Recent population genetic studies in the Zagros region have highlighted the importance of this genus as a focal point for understanding genetic diversity and speciation within challenging habitats. Despite the intrinsic scientific interest surrounding the *Asaccus* species in the Zagros, research efforts have been limited, and comprehensive studies covering the entire region are scarce. Since 1994, the number of *Asaccus* species recognized as endemic to the Zagros massif has risen to 10, indicating a growing but still incomplete understanding of their diversity and distribution [4, 8–20]. This incremental discovery highlights both the richness of the Zagros as a habitat for unique species and the urgent need for further, more expansive genetic and ecological studies to fully comprehend the range and evolutionary dynamics of the *Asaccus* genus in this biodiverse region.

In the present study, we aimed to provide insight into the taxonomy, phylogeny, and phylogeography of Southwest Asian leaf-toed geckos, covering samples from across the entire Zagros region, as well as examining their phylogenetic relations with samples from the Hajar Mountains of Oman and the United Arab Emirates (UAE). Our objectives were to (i) investigate the taxonomy of Southwest Asian leaf-toed geckos, (ii) uncover their phylogenetic relationships, (iii) examine the phylogeographic processes, including the timing of *Asaccus* divergence and colonization from the Hajar Mountains into Iran, and (iv) update the distribution range of *Asaccus* species in Iran.

## Materials and methods

### Taxon sampling

**Fig. 1:**
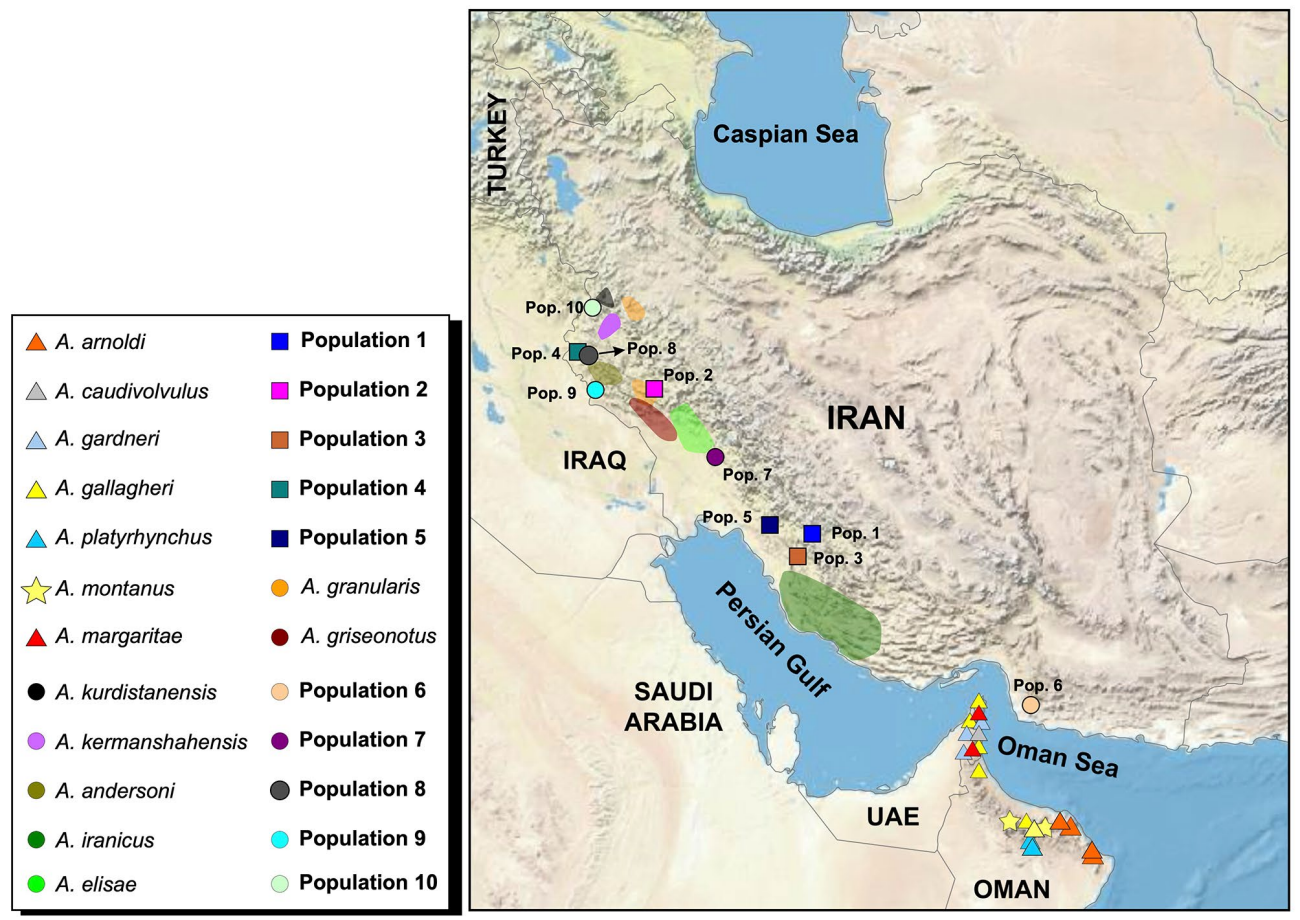
Geographical distribution of *Asaccus* populations sampled across their range, detailing the specific localities and the corresponding species identified at each site

We collected 106 *Asaccus* specimens from the Zagros Mountains between April 2017 and August 2019, as outlined in Table S1. All experimental protocols received approval from the Research Ethics Committee at the Natural Environment Zoological Museum of the University of Tehran (NEZMUT). The sampling and handling of animals were conducted in accordance with the relevant guidelines of the Animal Use and Care Committee at the Department of Environment of Iran (DOEI), under permit number 97/23,491. This study adheres to the ARRIVE guidelines, available at https://arriveguidelines.org. Specimen collection was performed manually during daylight hours and around sunset. Our objective was to collect samples from all previously described *Asaccus* species in Iran at their type localities to facilitate more reliable inferences. All 106 specimens underwent morphological analysis, while molecular analyses were conducted on 48 samples. Also, morphological data from 115 specimens from previous studies [12] were added to our morphological analysis. Importantly, all sampling procedures were non-invasive, and each specimen was released back into its habitat immediately after data collection. Additionally, we included sequence data from 241 specimens available at GenBank (comprising 239 *Asaccus* sequences and two outgroups) as reported by Carranza et al. [13], Simό-Riudalbas et al. [12], and Fattahi et al. [21]. A comprehensive list of all specimens, including their taxonomic identifications, GenBank accession numbers, sample voucher codes, and geographical ranges is provided in Table S1. *Ptyodactylus hasselquistii* [22] and *P. ruusaljibalicus* [23] served as outgroups. Therefore, a total of 347 samples from the Hajar Mountains as well as the Zagros range, extending from western to southern Iran, were analyzed in this study (Fig. 1, Tables S1 and S4).

### Molecular analyses

#### DNA extraction and amplification

DNA extraction and amplification was carried out for 48 samples out of 106 collected specimens (Table S1). Extraction of total genomic DNA from tail tissue samples was performed using Thermo Scientific GeneJET Genomic DNA Purification Kit (Thermofisher, Inc.). Two mitochondrial, the mitochondrial 12 S ribosomal RNA (12 S; 473 bp) and the cytochrome *b* (Cyt *b*; 399 bp), and one nuclear, the nuclear oocyte maturation factor MOS (*c*-*mos*; 567 bp) gene fragments were amplified. For 12 S, the polymerase chain reaction (PCR) parameters included pre-denaturation at 94 °C for 30s, 61 °C for 10s, and 72 °C for 30s for 33 cycles using the primers 12SaGekko and 12SbGekko (Table 1; Carranza et al., 2016). For Cyt *b*, thermal cycling conditions included pre-denaturation at 94 °C for 45s, 55 °C for 45s, and 72 °C for 10s for 40 cycles. For *c*-*mos*, PCR consisted of 35 cycles at 94 °C for 30s, 57.5 °C for 15s, and 72 °C for 30s using a pair of primers designed for this study (kam1 and kam2; Table 1). PCR products were electrophoresed in a 1% low melting agarose gel stained with ethidium bromide. Sequencing of the PCR products was performed by direct double-strand cycle sequencing in both directions on 3730XL DNA Analyzer (ABI) at the DNA Sequencing Core Facility of Academia Sinica, Taiwan. Also, sequence data of a total of 239 *Asaccus* and two *Ptyodactylus* individuals were obtained from GenBank and used in the molecular analyses.

Two datasets were used in our genetic analyses: Dataset 1, which contains 287 sequences of the 12 S gene used for genetic distance-based analyses, with sequences having missing data excluded to ensure accuracy and prevent misleading results [24]; and Dataset 2, comprising 137 sequences of the three genes (12 S, *c-mos*, and Cyt *b*) concatenated into a 1439 bp alignment for phylogenetic analyses. In Dataset 2, to avoid redundancy and focus on genetic diversity, the samples of *Asaccus* species from Arabia were reduced to a single sample for each haplotype.

**Table 1:** List of primers used for amplification and sequencing

Gene	Primer	Sequence (5’–3’)	source
12 S	12SaGekko 12SbGekko	F: CAAACTAGGATTAGATACCCTACTATGC	Carranza et al., 2016
		R: GAGGGTGACGGGCGGTGTGTAC	Carranza et al., 2016
Cyt *b*	Salvi1	F: GACTGGCATCATTTGTGTCTGCTRCA	Carranza et al., 2016
	Cytb2	R: CTTTAAGGAGTTCAGGAGCTCGATGGGT	Carranza et al., 2016
*c-mos*	Kam1	F: GACTGGCATCATTTGTGTCTGCTRCA	This study
	Kam2	R: CTTTAAGGAGTTCAGGAGCTCGATGGGT	This study

### Sequence analyses

We examined sequences using SeqScape version 2.6 and AliView version 1.25, both from Applied Biosystems, Darmstadt [25]. To ensure accuracy, all protein-coding gene fragments (Cyt *b* and *c-mos*) were translated into amino acids to verify the correct reading frames. For multiple sequence alignments, we employed MAFFT version 7 [26] with the default parameter settings (Auto strategy, Gap opening penalty: 1.53, offset value: 0.0). To facilitate comprehensive downstream phylogenetic analyses, sequences of the two mitochondrial gene fragments (12 S and Cyt *b*) and the nuclear gene (*c-mos*) were concatenated.

We computed genetic distances, translation/transversion ratios, and nucleotide composition for Dataset 1 using MEGA version 5 [27]. Pairwise genetic distances among populations were assessed using uncorrected pairwise genetic distances of the 12 S gene fragment, supported by 1,000 bootstraps (Table S2). Further, we estimated other genetic statistics, such as the number of haplotypes (h), haplotype diversity (Hd), polymorphic sites (S), nucleotide diversity (Pi), average number of nucleotide differences (K), Fu’s F values and their corresponding *p*-values, and Tajima’s D using DnaSP version 5.1 [28] (Table 2).

### Phylogenetic analyses and haplotype networks

To reconstruct phylogenetic relationships among the studied species, we utilized Maximum Likelihood (ML) and Bayesian Inference (BI) methods on Dataset 2. The best models of molecular evolution and the optimal partitioning schemes for Dataset 2 were determined using PartitionFinder version 2.1.1 [29], employing the Bayesian Information Criterion (BIC) for model selection and a greedy search algorithm for exploring partitioning schemes. We treated the 12 S gene as a single data block and partitioned Cyt *b* and *c-mos* by codons (Table S3). ML analyses were performed using IQ-TREE version 1.6.12 [30], adhering to the best-fitting model suggested by PartitionFinder (Table S3). The reliability of the ML trees was assessed through 1000 replicates using the Ultrafast bootstrap analysis method [31]. To prevent overestimation of support values, the ‘-bnni’ option, which improves branch support estimation, was applied.

We conducted BI tree inference using BEAST version 1.10.4 [32], with two independent runs comprising 2 × 10^8 MCMC iterations each, sampling every 20,000 generations to ensure a comprehensive exploration of the phylogenetic parameter space. A birth-death process was employed as the underlying model for these analyses [33], selected based on the best-fit partitioning schemes identified by PartitionFinder (Table S3), to accurately model lineage diversification over time. To assess the convergence and reliability of our analyses, we monitored the Effective Sample Sizes (ESS) and posterior trace plots using Tracer version 1.7.1 [34], ensuring that ESS values were sufficiently high to indicate reliable parameter estimation. The results from the individual runs were combined using LogCombiner, discarding the first 10% of samples as burn-in to eliminate the initial phase of each run where the model may not have fully stabilized. Finally, we generated the consensus ultrametric tree using TreeAnnotator version 1.10.4, both tools being integral components of the BEAST software package.

We reconstructed two haplotype networks using Dataset 1 to elucidate genetic relationships at both intra- and inter-specific levels. For intra-specific analyses, we employed the TCS method within PopArt version 1.7 to examine haplotypes within each lineage. For inter-specific relationships, we generated a neighbor-net network based on uncorrected patristic distances, a measure of genetic distance, in SplitsTree version 4.6 [35], utilizing 1,000 bootstrap replicates to assess the robustness of the network. This dual approach allows for a comprehensive understanding of genetic relationships across and within species.

### Molecular divergence times

We estimated species divergence times using BEAST, employing previously published mean rates of evolution for Cyt *b* (mean: 0.0228, standard deviation: 0.00806) and 12 S (mean: 0.00755, standard deviation: 0.00247) based on prior specifications [12, 13]. For mitochondrial genes, we used an uncorrelated lognormal clock to account for rate variation among branches, and a strict clock for the nuclear gene to assume a constant rate of evolution across the tree. A random starting tree was applied along with a uniform base substitution prior (0,100) and a uniform alpha prior (0,10) to model rate variation among sites. Clock and substitution models were kept unlinked to allow for independent rate variation. To accurately account for heterozygous sites in nuclear gene partitions and prevent their interpretation as missing data, we manually modified the XML file to Ambiguities = “true”. This ensures that all genetic information contributes to the analysis, enhancing accuracy. Furthermore, we incorporated a secondary calibration point, derived from previous publications, for time-tree calibration. Specifically, we set the divergence time between two *Asaccus* clades in Oman at 10–25 Mya, with a normal distribution (mean: 16.13, standard deviation: 1), as reported by Simó-Riudalbas et al. [12]. Nodes were considered strongly supported if they had posterior probabilities (PP) greater than or equal to 0.95 and ML bootstrap values greater than or equal to 70%, following established thresholds for robust phylogenetic inferences [36].

### Species delimitation analyses

To determine species boundaries within the genus *Asaccus*, we employed novel taxonomic methods for species delimitation, integrating both single-locus and multi-locus datasets. We applied three single-locus tree-based methods to phylogenetic trees inferred from the mitochondrial dataset (12 S and Cyt *b*): the Bayesian Poisson Tree Process model (bPTP) [37], which estimates speciation rates in a Bayesian framework; the single threshold General Mixed Yule-Coalescent model (GMYC) [38, 39], designed to identify the transition point between intraspecific coalescence and interspecific diversification; and the Multi-rate PTP (mPTP) [40], which extends the PTP model by allowing for variable rates of evolution across branches, thereby accommodating the differential evolutionary rates observed among species.

Additionally, we applied a multi-locus method via the Bayesian Phylogenetics and Phylogeography (BPP) program version 3.4 y [41] to the 12 S, Cyt *b*, and *c-mos* genes. This approach incorporates the multispecies coalescent model, effectively accommodating incomplete lineage sorting and gene tree heterogeneity. The BPP analysis was initiated with a random starting tree, applying uniform base substitution priors (0,100) and uniform alpha priors (0,10) to model rate variation among sites, which is crucial for accurately inferring species delimitation under the multispecies coalescent model.

For the bPTP analysis, we ran 500,000 MCMC generations with a burn-in of 10% and a thinning interval of 500, assessing convergence through MCMC trace vs. log-likelihood plots—a critical step to ensure the robustness of species delimitation results. The mPTP model’s effectiveness in dealing with unevenly sampled datasets was leveraged by its unique approach to model evolution rates (λ) individually for each species branch, contrasting with the single-rate assumption in traditional PTP models. This method’s reliance on the Akaike Information Criterion for model selection allows for a more nuanced understanding of species boundaries.

The GMYC analysis was conducted on a summarized ultrametric tree obtained from BEAST, using the ‘splits’ R package [42], which facilitates the identification of speciation events based on branching patterns within the tree. Finally, the BPP analysis was further refined by setting Ambiguities to “true” in the XML file to account for heterozygous sites, thereby avoiding their treatment as missing data. This adjustment ensures that all genetic information contributes to species delimitation. A guide tree from the concatenated dataset was used in BEAST, with the A10 mode for species delimitation (species tree = 0, species delimitation = 1), adopting a conservative approach where only speciation events with posterior probabilities ≥ 0.99 were considered for analyses [43].

To compare tree-based and distance-based methods of species delimitations, we implemented a distance-based approach using the Automatic Barcode Gap Discovery method (ABGD) [44]. ABGD utilizes a model-based confidence limit to distinguish haplotypes as separate “species” by identifying interspecific divergence through barcode gaps, which are significant genetic differences not observed within species. This method iteratively performs cluster inference and gap detection on the results of the previous clustering stage, leading to a final partition [45]. We processed our alignment through the ABGD web interface (https://bioinfo.mnhn.fr/abi/public/abgd/abgdweb.html), selecting the Jukes-Cantor (JC69) substitution model. This model was chosen for its ability to account for base substitution without assuming equal rates of change across bases, which is appropriate for our dataset’s nature. The analysis parameters were set with a prior for the maximum value of intraspecific divergence ranging from 0.001 to 0.1, a gap width of 1.0, and 15 recursive steps to ensure thorough exploration of potential species boundaries.

### Morphological analyses

#### Morphological samples and variables

In total, 106 *Asaccus* specimens from the Zagros Mountains were evaluated for morphological, morphometric and meristic analyses (Table S4). Only groups comprising three or more specimens were included to ensure statistical robustness in the morphological analyses. Additionally, morphometric data for 115 vouchered specimens were incorporated from previously published literature [12, 13], of which 61 included meristic data, enriching our dataset with comparative historical benchmarks. Morphometric variables were meticulously measured on the right side of each specimen using a vernier caliper accurate to 0.02 mm. The variables included head length (HL), head height (HH), head width (HW), eye diameter (ED), snout length (SL), snout-vent length (SVL), snout width (SW), trunk length (TrL), humerus length (LHu), femur length (LFe), ulna length (LUn), and tibia length (LTb). Tail length was excluded from the measurements due to the prevalence of lost or regenerated tails, which could introduce inconsistencies. For meristic measurements, five characters were quantified under a dissecting microscope: the number of lower labial scales (LLS), longitudinal rows of dorsal tubercles (Tlrow), number of postmentals (PMS), number of upper labial scales (ULS), and number of expanded lamellae rows under the 4th toe (LT4), providing a comprehensive suite of morphological data to assess species variation and delineation.

### Multivariate analyses

To investigate size and shape differences between *Asaccus* species, we performed independent analyses on 12 morphometric and five meristic characters. To normalize the data and ensure homogeneity of variance, all measurements were log10-transformed. The refined dataset comprised 221 specimens for morphometric analyses and 167 for meristic analyses (Table S4). We then conducted a Principal Component Analysis (PCA) on the correlation matrix, a technique that reduces the dimensionality of the data while preserving most of the variation, to visually assess shape variation between species. SVL values were specifically excluded from the PCA to focus on shape differences independent of overall size. Following this, a one-way ANOVA was applied to each principal component to determine how individual variables contribute to morphometric differences between species. This test was chosen for its ability to identify statistically significant differences between group means. Similarly, one-way ANOVA was utilized for all pairs of species using log-transformed SVL measurements to assess size differences. Additionally, for taxonomic delimitations, we applied one-way ANOVA to each meristic and morphometric variable, testing for morphological differences between pairs of species, ensuring a comprehensive evaluation of both size and shape variations.

## Results

### Genetic variation

Dataset 2 contained a concatenated alignment of 1,439 base pairs (bp) consisting of 342 variable (*V*) and 299 parsimony informative (*PIS*) sites (473 bp 12 S: *V* = 137, *PIS* = 121; 399 bp Cyt *b*: *V* = 180, *PIS* = 157; 567 bp *c*-*mos*: *V* = 25, *PIS* = 21) for 127 individuals. The base composition of the sequences was T = 24.2%, C = 25.9%, A = 29.6% and G = 20.3%.

We found 60 haplotypes in the 287 sequences of 12 S from Dataset 1, of which 32 haplotypes were identified as Iranian leaf-toed geckos. Nucleotide diversity ranged from 0.00096 (*A. margaritae*) to 0.0825 (*A. griseonotus*). Haplotype diversity ranged from 0.359 (*A*. *margaritae*) to 0.900 (**Population 1**). The Fu’s *F*s test returned a significantly positive value, also the highest value, for *A*. *griseonotus* (9.682; *p* = 0.001) and *A. arnoldi* (8.421; *p* = 0.002), respectively. Although Fu’s *F*s values for other species were not significant, positive values were found in *A*. *montanus*, *A*. *elisae*, *A*. *granularis*, *A*. *kermanshahensis*, **Population 4**, and **Population 3**, suggesting that these species might have been subjected to bottleneck events or genetic drift. Furthermore, negative values found in, *A. gardneri*, *A*. *gallagheri*, *A*. *iranicus*, *A*. *kurdistanensis*, and **Population 1** indicate that these species might have undergone recent demographic expansions. All measured genetic characteristics are shown in Table 2.

**Table 2:** Genetic characteristics of *Asaccus* species with sample sizes greater than four (n: number of samples, h: number of haplotypes, HD: haplotype diversity, *Pi*: nucleotide diversity, K: average numbers of nucleotide differences, Fu’s *F*s: a measure of selection or population expansion, P: Fu’s *p*-value, Tajima’s D: Tajima’s D value). The values were calculated from sequences of Dataset 1

Species		*n*	h	HD (SD)	Pi (SD)	K	S	Fu’s FS	*P*	Tajima’s D
*A. arnoldi*		13	4	0.718 (0.089)	0.03431 (0.00317)	12.077	24	8.421	0.002	2.43201
*A. gardneri*		42	3	0.516 (0.077)	0.0024 (0.00072)	0.8595	8	–0.689	0.179	–1.51843
*A. gallagheri*		57	12	0.827 (0.029)	0.0140 (0.00434)	4.901	63	0.775	0.138	–2.26371
*A. margaritae*		27	2	0.359 (0.091)	0.00096 (0.00024)	0.3589	1	0.975	0.379	0.61939
*A. montanus*		33	12	0.881 (0.031)	0.04584 (0.00212)	17.464	38	5.991	0.005	2.79788
*A. elisae*		8	2	0.536 (0.123)	0.0015 (0.00035)	0.5357	1	0.866	0.411	1.16650
*A. granularis*		12	4	0.697 (0.090)	0.0098 (0.00341)	3.424	13	2.372	0.162	–0.86645
*A. griseonotus*		11	5	0.818 (0.083)	0.0825 (0.03469)	31.2	95	9.682	0.001	–0.18227
*A. iranicus*		33	5	0.799 (0.046)	0.0042 (0.00034)	1.473	7	–2.200	0.062	–0.42095
*A. kermanshahensis*		4	2	0.667 (0.204)	0.01326 (0.00406)	4.6667	7	3.856	0.177	2.17956
*A. kurdistanensis*		5	3	0.700 (0.218)	0.00237 (0.00089)	0.800	2	–0.829	0.244	–0.97256
**Population 4**		5	2	0.600 (0.175)	0.00336 (0.00098)	1.200	2	1.688	0.390	1.45884
**Population 1**		5	3	0.900 (0.161)	0.00712 (0.00194)	2.800	6	–0.445	0.305	0.19092
**Population 3**		5	2	0.400 (0.0563)	0.00339 (0.00201)	1.200	3	1.688	0.390	–1.04849

### Phylogenetic and haplotype network reconstructions

**Fig. 2:**
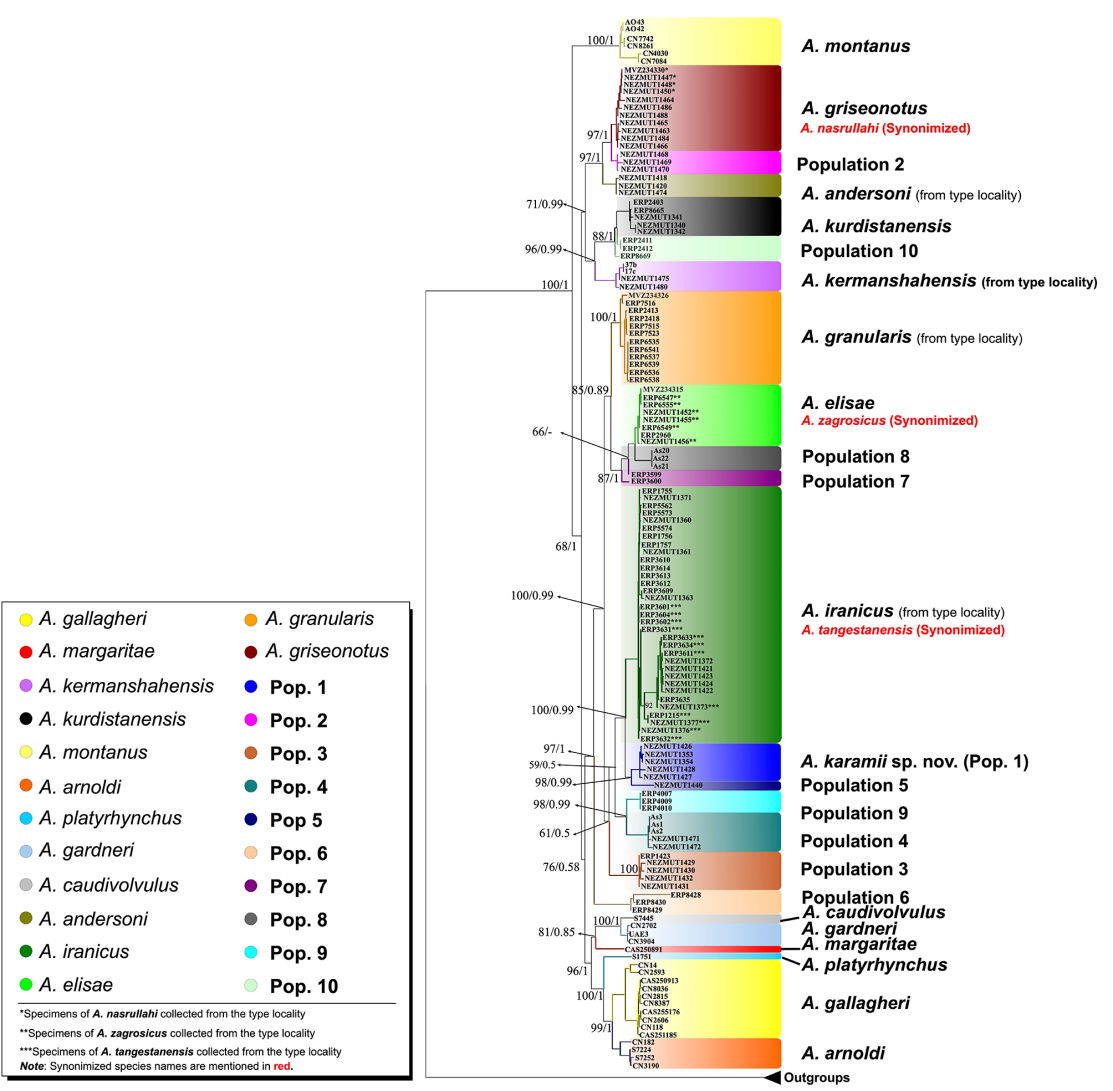
Phylogenetic relationships of *Asaccus* species using the concatenated dataset of two mitochondrial (Cyt *b* and 12 S) and one nuclear (*c*-*mos*) genes. PP and ML bootstrap support values are shown next to the nodes

The BI and ML phylogenetic analyses of Dataset 2 yielded congruent trees, with the majority of nodes being well-supported (ML bootstrap values > 70%, PP > 0.95; Fig. 2). For the Bayesian tree inference, posterior trace plots and effective sample size (ESS > 200) of the runs were assessed in Tracer to ensure convergence. In both reconstructions, the Hajar endemic *A. montanus* emerged as the basal lineage to the remaining lineages with considerably high BI PP (1.00) and ML bootstrap (100) support values. Furthermore, *A. andersoni*, *A*. *kermanshahensis*, *A*. *kurdistanensis*, *A*. *griseonotus*, *A*. *nasrullahi*, **Population 2**, and **Population 10** in northern Zagros diverged into well-supported clades with high BI PP (1.00) but moderate ML bootstrap (68) values and separated from the other Iranian (*A*. *elisae*, *A*. *zagrosicus*, *A*. *iranicus*, and *A*. *tangestanensis*) and Arabian (*A*. *arnoldi*, *A*. *caudivolvulus*, *A*. *gardneri*, *A*. *gallagheri*, *A*. *margaritae*, and *A*. *platyrhynchus*) *Asaccus* species (Fig. 2). *A*. *granularis* samples from the type locality (ERP2413, ERP2418, ERP6535, ERP6536, ERP6537, ERP6538, ERP6539, ERP6541, ERP7515, ERP7516, ERP7523) and those collected in this study from north of Pol-e-Dokhtar, close to the type locality (NEZMUT1468, NEZMUT1469, and NEZMUT1470; herein referred to as **Population 2**) were separated into two distinct clades (Fig. 2). However, we found no evidence of significant divergence between *A. zagrosicus* and *A*. *elisae* as all *A. zagrosicus* specimens collected from the type locality (NEZMUT1452, NEZMUT1455, NEZMUT1456, ERP2960, ERP6547, ERP6549, ERP6555) were nested within *A. elisae*. Similarly, no significant genetic differentiation was found among *A*. *iranicus* and *A*. *tangestanensis*, as all specimens of *A*. *iranicus* and *A*. *tangestanensis* formed a single well-supported clade. One specimen from the Museum of Vertebrate Zoology (MVZ 234,326) deposited in GenBank as *A. griseonotus* collected from 99 km SW of Khorram Abad, western Iran, clustered within *A. granularis* (ERP2413, ERP2418, ERP6535, ERP6536, ERP 6537, ERP6538, ERP6539, ERP7515, ERP7516 and ERP7523), suggesting a possible error in this record [21]. Our results of the phylogenetic trees, species delimitation analyses and haplotype networks also implied that *A. nasrullahi* and *A. griseonotus* should be treated as a single taxon. The clade containing the specimens of these two species is sister to **Population 2** with high support (1.00 PP and 97 bootstrap). In addition to the specimens collected in this study from the type locality, the specimen from the Museum of Vertebrate Zoology (MVZ234330) deposited in GenBank as *A. nasrullahi* also clustered within *A. griseonotus*.

**Fig. 3:**
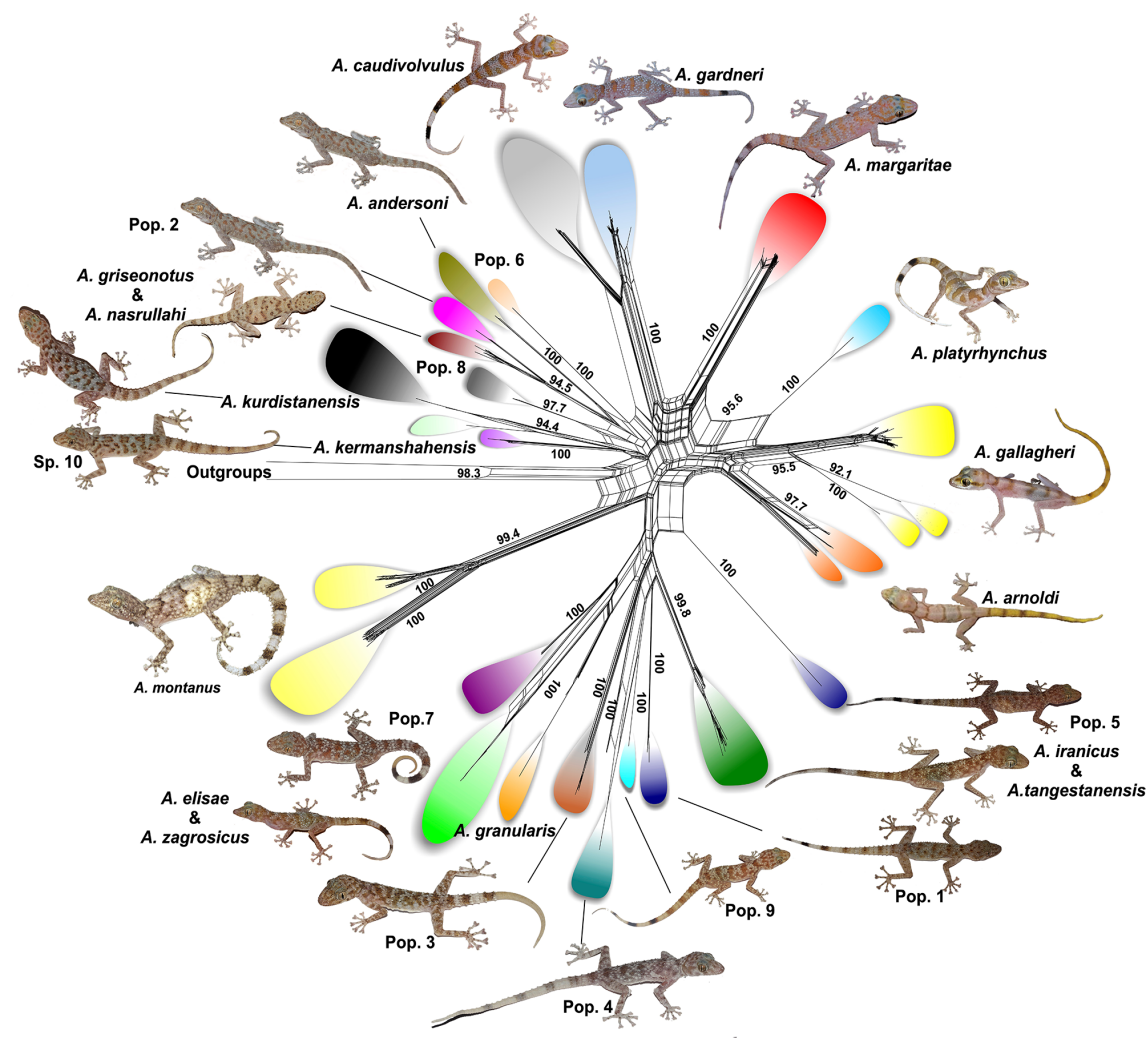
SplitsTree network based on a 473-bp dataset of 12 S (287 sequences), showing 28 distinct haplogroups within *Asaccus*. Colors correspond to those shown in Fig. 1. Numbers represent bootstrap support values. *Ptyodactylus hasselquistii* and *P. ruusaljibalicus* were used as outgroups

**Fig. 4:**
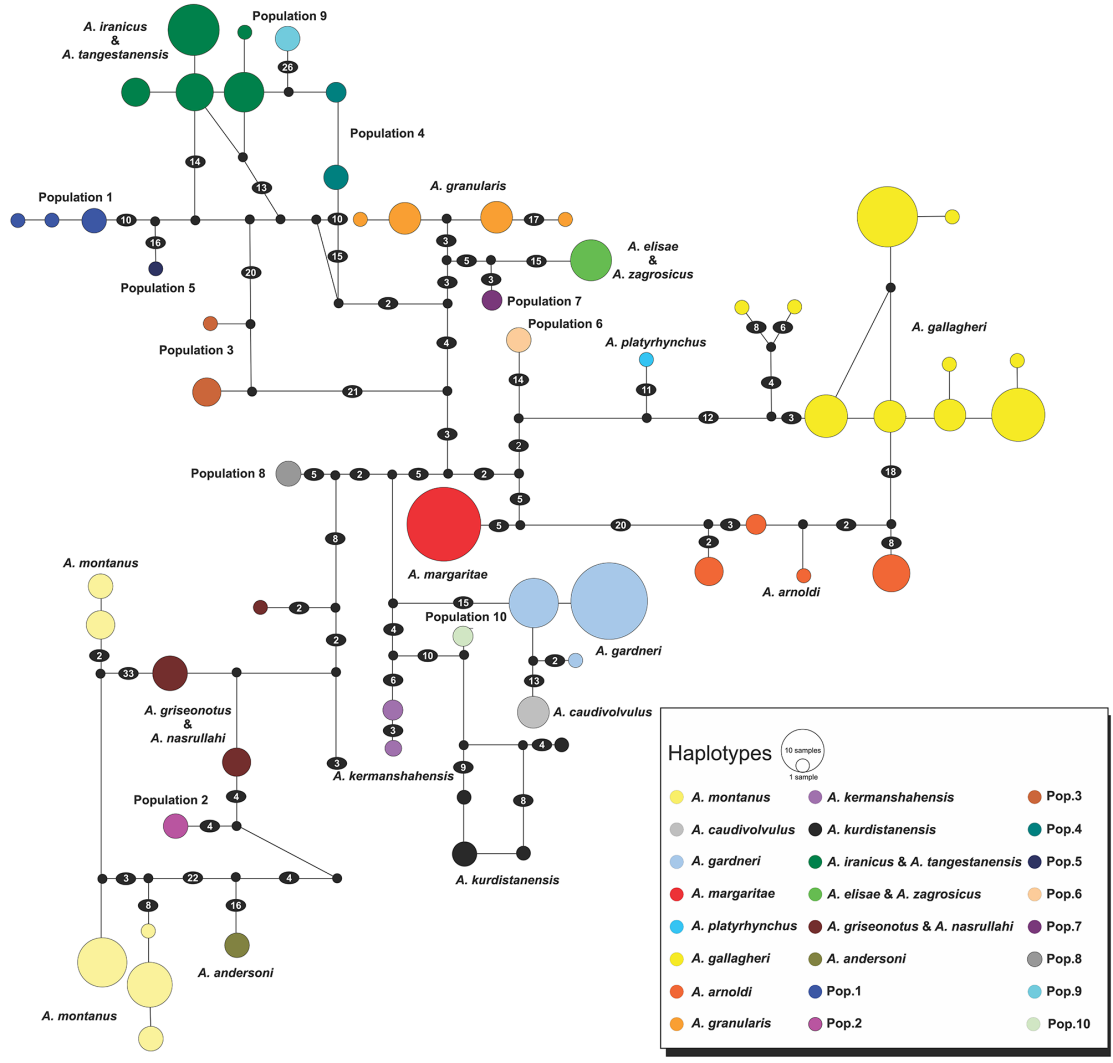
Haplotype network using a total of 287 sequences of 12 S (473 bp) constructed in PopArt using the TCS method. Numbers in the ellipses indicate mutational steps among haplotypes. Lines between two haplotypes indicate one mutation. Circle sizes are proportional to haplotype frequencies. Black circles represent extinct or unsampled haplotypes

In accordance with the phylogenetic tree, the haplotype network constructed in SplitsTree using the 12 S matrix (287 *Asaccus* samples and two *Ptyodactylus* samples as outgroups) identified 28 distinct haplogroups within *Asaccus* (Fig. 3). Similarly, the TCS haplotype network generated by PopArt detected 60 haplotypes using 287 sequences of 12 S (Fig. 4).

A clear result of the haplotype network is that *A*. *granularis* specimens are separated from those collected during the present study from north of Pol-e-Dokhtar, close to the type locality (Figs. 3 and 4). In addition, *A*. *griseonotus* from the type locality (MVZ234326) [12, 13, 21, 46, 47] did not share haplotypes with the specimens collected by the present study from Pol-e-Dokhtar. This agreed with the phylogenetic trees where these specimens clustered into separate clades. The two species *A*. *elisae* and *A*. *zagrosicus* shared a haplogroup and fell into the same phylogenetic clade (Fig. 2); similarly, *A*. *tangestanensis* and *A*. *iranicus* were recognized as a single taxon. Moreover, *A*. *montanus*, *A*. *gallagheri*, and *A*. *arnoldi* each consisted of more than one haplogroup. For *A. montanus*, Tamar et al.’s [46] study revealed two western and eastern populations.

### Molecular divergence times

**Fig. 5:**
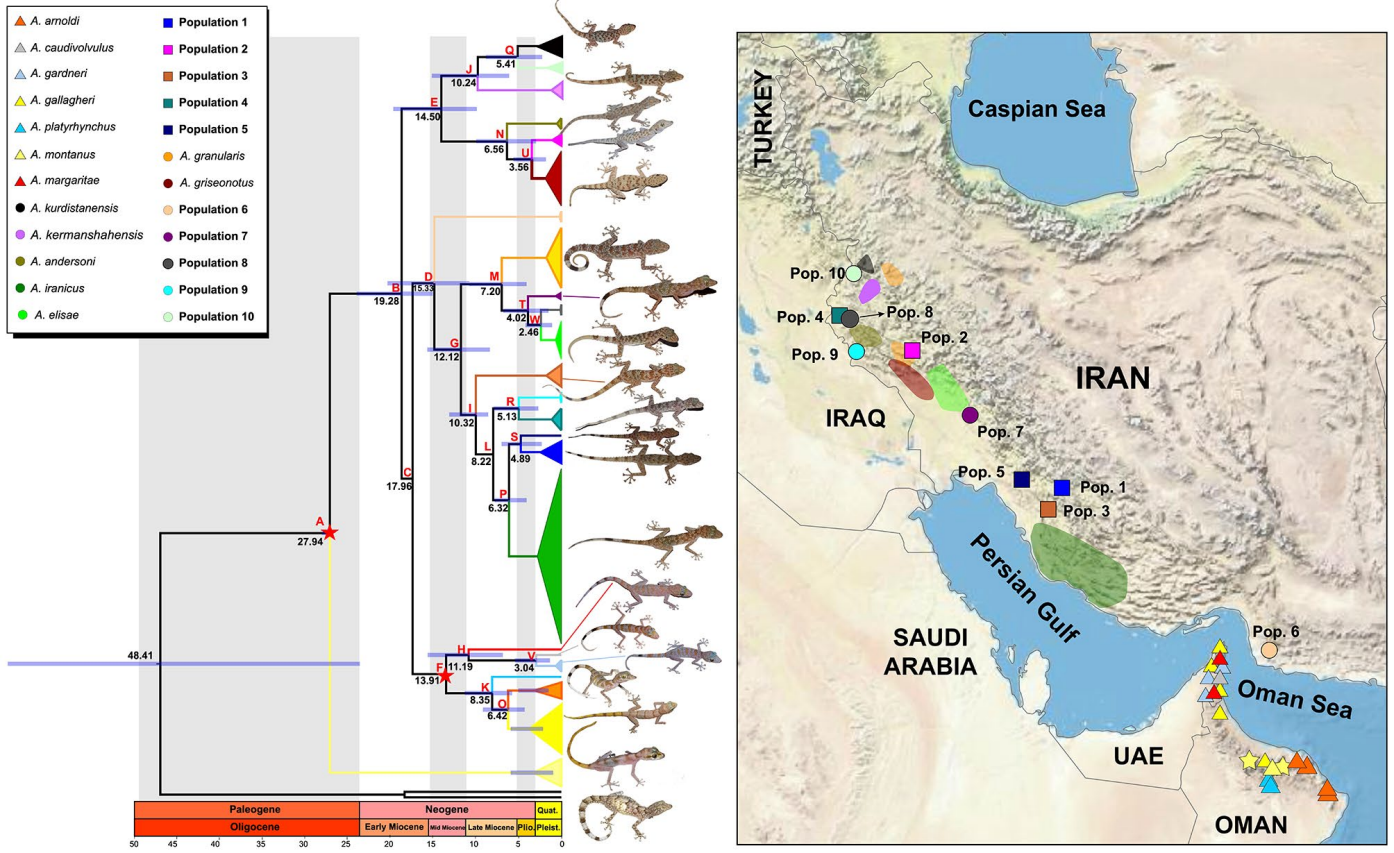
Bayesian estimates of *Asaccus* divergence using the concatenated dataset of two mitochondrial (12 S and Cyt *b*) and one nuclear (*c*-*mos*) genes. Age estimates are given behind the nodes and purple bars denote the mean and HPD 95% confidence intervals. The node with the red star indicates the calibration point. NZ: Northern Zagros, CZ: Central Zagros, and SZ: Southern Zagros

The analysis of divergence times placed the most basal split within Southwest Asian leaf-toed geckos in the Late Oligocene (Chattian Age) at ∼27.94 Mya (95% HPD: 26.96–28.91 Mya; Fig. 5: node A), with *A. montanus* emerging as the sister taxon to all *Asaccus* species. Subsequently, cladogenetic events dating back to the Early Miocene (Burdigalian Age) separated *A*. *kurdistanensis*, *A. kermanshahensis*, *A*. *andersoni*, *A*. *griseonotus*, **Population 2**, and **Population 10** from the other *Asaccus* species in Iran and Arabia at ∼19.28 Mya (95% HPD: 14.57–23.86 Mya; Fig. 5: node B). More recent divergence events dating back to the Early Miocene (Burdigalian Age) at ∼17.96 Mya (95% HPD: 13.46–22.50 Mya; Fig. 5: node C) split *Asaccus* species of the Hajar Mountains from the other species in Iran. The divergence of **Population 6** from the other *Asaccus* species of Zagros occurred in the Middle Miocene (Langhian Age) at ∼15.33 Mya (95% HPD: 10.80–20.28 Mya; Fig. 5: node D). Other divergence events dating back to the Middle Miocene (Langhian Age) at ∼14.50 Mya (95% HPD: 9.60–19.62 Mya; Fig. 5: node E) separated *A*. *kermanshahensis*, *A*. *kurdistanensis* and **Population 10** from *A*. *griseonotus*, *A*. *andersoni*, and **Population 2** in northern Zagros. The molecular dating analysis indicated that cladogenetic events dating to the Middle Miocene (Langhian Age) at ∼13.91 Mya (95% HPD: 9.69–18.27 Mya; Fig. 5: node F) initiated the divergence among *Asaccus* species of the Hajar Mountains (*A*. *arnoldi*, *A*. *margaritae*, *A*. *gardneri*, *A*. *caudivolvulus*, *A*. *platyrhynchus*, and *A*. *gallagheri*). Furthermore, **Population 7** in central Zagros and **Population 8**, *A. elisae*, and *A. granularis* in northern Zagros diverged from those in southern Zagros in the late Middle Miocene (Serravallian Age) at ∼12.12 Mya (95% HPD: 8.49–16.04 Mya; Fig. 5: node G). The next split occurred during the Late Miocene (Tortonian Age) at ~ 11.19 Mya (95% HPD: 7.03–15.64 Mya; Fig. 5: node H), reflecting the divergence of *A. margaritae* from *A. caudivolvulus* and *A. gardneri*. Shortly after that, **Population 3** radiated from the other southern Zagros species in the Late Miocene (Tortonian Age) at ~ 10.32 Mya (Fig. 5; node I). At approximately the same time, *A*. *kurdistanensis* separated from *A*. *kermanshahensis* in northern Zagros during the Late Miocene (Tortonian Age) at ~ 10.24 Mya (95% HPD: 5.92–14.93 Mya; Fig. 5: node J). Following that, *A*. *platyrhynchus* diverged from *A*. *arnoldi* and *A*. *gallagheri* in the Late Miocene (Tortonian Age) at ∼8.35 Mya (95% HPD: 5.31–11.64 Mya; Fig. 5: node K). Diversification of *A*. *iranicus*, **Population 1**, and **Population 5** from **Population 4** and **Population 9** took place in the Late Miocene (Tortonian Age) at ∼8.22 Mya (95%HPD: 5.50–11.15 Mya; Fig. 5: node L). Subsequently, *A. granularis* diverged from *A*. *elisae*, **Population 8**, and **Population 7** in the Late Miocene (Messinian Age) at ∼7.20 Mya (95% HPD: 4.13–10.71 Mya; Fig. 5: node M). Divergence of *A*. *andersoni* from *A*. *griseonotus* and **Population 2** occurred in the Late Miocene (Messinian Age) at ∼6.56 Mya (95% HPD: 3.28–10.07 Mya; Fig. 5: node N), shortly after which *A. arnoldi* split from *A. gallagheri* at ∼6.42 Mya (95% HPD: 4.06–9.09 Mya; Fig. 5: node O). At 6.32 Mya (Messinian Age) (95% HPD: 4.02–8.82 Mya; Fig. 5: node P), *A. iranicus* split from **Population 1** and **Population 5.** Another Late Miocene divergence event at 5.41 Mya (Messinian Age) (95% HPD: 2.39–8.97 Mya; Fig. 5: node. Q) split *A. kurdistanensis* from **Population 10**. Also, **Population 9** and **Population 4** are estimated to have diverged during Pliocene at ∼5.13 Mya (Zanclean Age) (95% HPD: 2.39–8.10 Mya; Fig. 5: node R). Subsequent splits occurred within Pliocene, with **Population 1** diverging from **Population 5** at ~ 4.89 Mya (Zanclean Age) (95% HPD: 2.83–7.16 Mya; Fig. 5: node S), and **Population 7** from **Population 8** and *A. elisae* at ~ 4.02 Mya (95% HPD: 1.57–7.01 Mya; Fig. 5: node T). The divergence between *A. griseonotus* and **Population 2** occurred in the Late Pliocene at ~ 3.56 Mya (Piacenzian Age) (95% HPD: 1.83–5.55 Mya; Fig. 5: node U). During the Late Pliocene-Early Pleistocene, *A. caudivolvulus* split from *A. gardneri* at ~ 3.04 Mya (95% HPD: 1.21–5.29 Mya; Fig. 5: node V). Finally, the split between *A*. *elisae* and **Population 8** occurred within the Early Pleistocene at ∼2.46 Mya (95% HPD: 0.97–4.17 Mya; Fig. 5: node W).

### Species delimitation

**Fig. 6:**
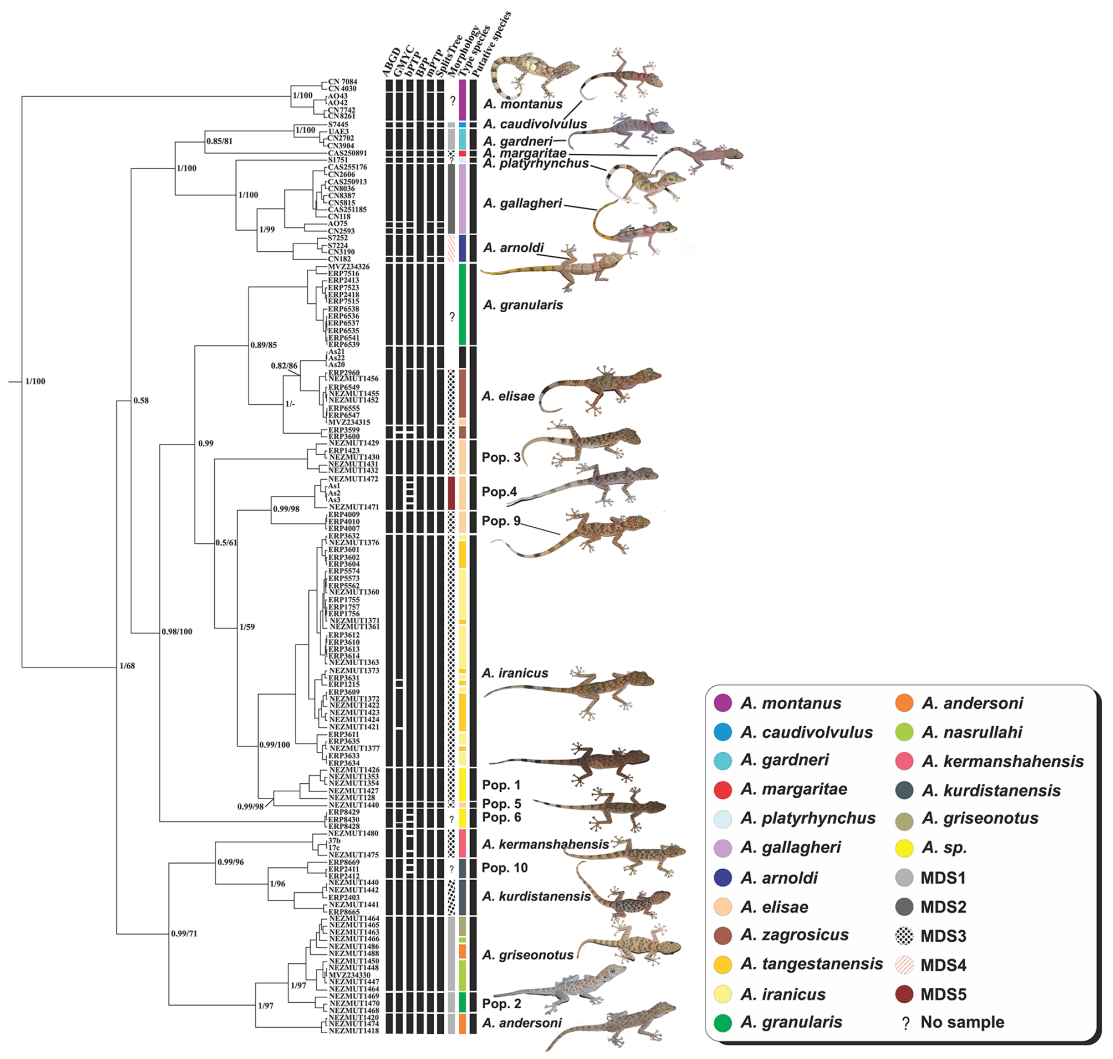
Bayesian phylogenetic tree and species delimitation approaches. The phylogenetic tree was obtained using a concatenated partitioned dataset including 12 S, Cyt *b*, and *c*-*mos*. PP and bootstrap values are given in front of each node. Taxon vouchers are given at branch tips. Species delimitation approaches included ABGD, GMYC, bPTP, BPP, mPTP, SplitsTree network and morphology. Question marks in the morphology column indicate no morphological data for that taxon. The type species column indicates species names based on the original publications. The putative species column corresponds to the proposed species based on consensus data (MDS: Morphologically Diagnosed Species)

The tree-based and distance-based methods of species delimitation yielded incongruent species boundaries and showed considerable differences in the number of well-delimited species within the genus *Asaccus*. With a total of 23 species, the BPP algorithm (based on the guide tree and the multi-locus sequence file) suggested the fewest number of species, while bPTP (based on the ML tree and the MCMC method) detected the greatest number of species (39). GMYC suggested 32 species (confidence interval 18–40; likelihood ratio 27.91426; threshold time − 3.40537), whereas the mPTP algorithm (based on the ML tree) proposed 25 species. The *p*-distance values calculated by ABGD reflected divergence levels typically expected within and between species, indicating highly supported species among the focal groups. Interspecific *p*-distance values ranged from 9 to 10% between *Asaccus* species. In total, ABGD (Fig. 6) proposed 28 species. All of the approaches outlined above agreed that *A*. *platyrhynchus*, *A*. *margaritae*, *A*. *andersoni*, *A. griseonotus*, *A. granularis*, *A. kurdistanensis*, **Population 1**, **Population 2**, **Population 3**, **Population 5**, and **Population 9** each represent a single taxonomic unit. All the approaches, except BPP, consistently suggested the existence of two distinct species in *A*. *montanus*. This is in line with Tamar et al.’s [46] study which identified two populations (eastern and western) for *A*. *montanus*. Similarly, all the approaches, except BPP, delimited *A. arnoldi* as two separate species. Likewise, only BPP suggested lumping *A. caudivolvulus* and *A. gardneri* into a single species. BPP also recovered *A*. *gallagheri* as one species, while ABGD, bPTP, and GMYC proposed three species and mPTP recovered two species for this taxon. In all methods, **Population 6** was treated as a single species, except for bPTP and GMYC which suggested three and two species for **Population 6**, respectively. Only mPTP identified *A*. *elisae*, **Population 7**, and **Population 8** as one single species (**Population 7** and **Population 8** are not included in Fig. 6 as both are considered synonymous to *A. elisae*). With the exception of bPTP, all approaches identified one species for **Population 4**. Also, in all approaches, *A*. *iranicus* and *A*. *tangestanensis* were delineated as one single species, except for GMYC which treated them as four species. With the exception of bPTP, all approaches recovered *A. kermanshahensis* and **Population 10** as one single species.

### Multivariate analyses of morphological data

**Fig. 7:**
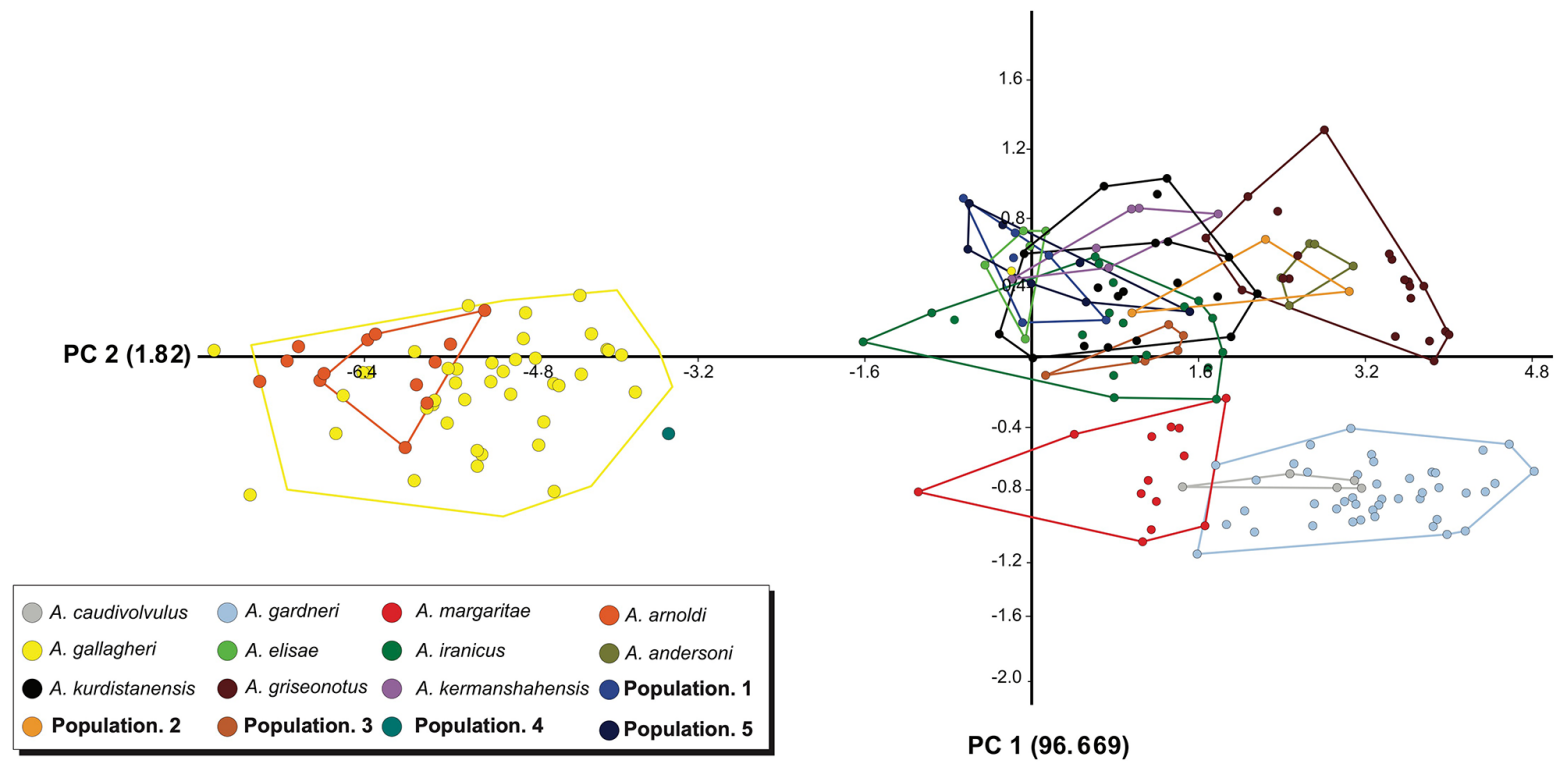
Principal component analysis of morphological measurements; PC1 vs. PC2.

We detected no significant sexual dimorphism in the 12 morphometric and five pholidotic characters. Sexual dimorphism analyses showed no significant differences in all morphometric characters. Therefore, to test shape differences, data from both sexes were pooled for analyses. Interspecific shape differences were evaluated using a one-way ANOVA on the PCA scores for the 11 components. The first component, representing 96.699% of the total variability, showed a significant difference (*P* < 0.0001), which represents almost all of the variability. The results of the PCA analysis for body shape (Fig. 7) showed that *A*. *arnoldi* was nested within *A*. *gallagheri*. Also, **Population 4** was placed near *A. gallagheri* and *A. arnoldi* of Oman. Moreover, *A*. *caudivolvulus* was nested within *A*. *gardneri*. The results also found that *A. caudivolvulus*, *A*. *gardneri* and *A*. *margaritae* were closer to *Asaccus* species of Iran than other Arabian species. Finally, the PCA results indicated that *Asaccus* species of Iran formed a morphologically similar group, which differed from *Asaccus* species of Arabia. Shape differences described above are shown in Fig. 7.

## Discussion

### The phylogenetic relationships of the genus *Asaccus*

The present study provides significant insights into the evolutionary relationships of leaf-toed geckos from Southwest Asia, particularly those within the *Asaccus* genus in Iran. We reconstructed these relationships by analyzing new sequences from Iranian *Asaccus* populations along with available GenBank data from both Iran and Arabia [12, 13, 21–23]. Our analysis identified 22 distinct lineages, elucidating a well-resolved phylogenetic structure for the *Asaccus* complex. Consistent with earlier multi-locus studies [12, 13, 46], our results support the monophyly of the *Asaccus* group, positioning *A. montanus* as the basal lineage relative to other *Asaccus* species. Additionally, our study identifies three new cryptic lineages, enhancing the phylogenetic framework established in previous research [21]. In line with findings by Papenfuss et al. [47]. , Carranza et al. [13]. , Simό-Riudalbas et al. [12]. , and Tamar et al. [46]. , we confirm the placement of the Hajar endemic *A. montanus* as sister to other *Asaccus* species. This contrasts with the study by Fattahi et al. [21]. , which proposed a different phylogenetic relationship, recovering *A. montanus*, *A. gallagheri*, *A. platyrhynchus* and two unresolved specimens as sister to all other species in the genus.

Moreover, our results differ from Fattahi et al. [21]. in several key aspects, wherein the clade comprising *A*. *margaritae*, *A*. *caudivolvulus*, and *A*. *gardneri* was not recovered as the sister clade to the other three Arabian species. Also, our study recovered **Population 6** as sister to *A*. *iranicus*, *A*. *elisae*, *A*. *granularis*, **Population 1**, **Population 3**, **Population 4**, **Population 5**, **Population 7**, **Population 8**, and **Population 9**; in contrast, Fattahi et al. [21]. placed **Population 6** as sister to all Iranian *Asaccus* species, except the two unresolved species that were nested within *A*. *montanus*. Moreover, the clade containing *A*. *griseonotus*, *A*. *andersoni*, *A*. *nasrullahi*, *A*. *kermanshahensis*, *A*. *kurdistanensis*, and **Population 10** was recovered as the sister clade to all other *Asaccus* species, while Fattahi et al. [21]. placed this clade as sister to Iranian Southwest Asian leaf-toed geckos only.

### Divergence and colonization history of southwest Asian leaf-toed geckos

Our divergence time estimates are consistent with those reported by Tamar et al. [46]. , despite their use of different mean rates of molecular evolution for concatenated datasets of mitochondrial and nuclear markers. Similarly, our estimates align closely with those of Carranza et al. [13]. , who used the same mean rates for mitochondrial markers as we did, although our estimate for the divergence time of *A. montanus* is slightly older (27.94 Mya compared to 24.6 Mya). Additionally, our phylogenetic tree shares six nodes with the tree from Simό-Riudalbas et al. [12]. , with three specific nodes (nodes A, K, and O; Fig. 5) exhibiting similar divergence times. In contrast, our study found no congruence in tree topology or divergence times with the study by Fattahi et al. [21]. , precluding any meaningful comparison between these two sets of results.

The dated phylogenetic tree analysis reveals a sister-taxon relationship between *Asaccus* and *Ptyodactylus*, suggesting an Arabian center of origin for *Asaccus*. Additionally, molecular evidence has identified *A. montanus*, an endemic species from the Hajar Mountains, as the sister taxon to other *Asaccus* species [47]. This supports the hypothesis that the colonization of *Asaccus* began in the Hajar Mountains of Arabia. However, this conclusion challenges the hypothesis proposed by Rastegar-Pouyani et al. [8]. , which suggested that the Zagros Mountains were the original habitat of the genus.

The results of molecular dating indicated that the divergence between *Asaccus* species and their sister taxa took place in the Late Oligocene (27.94 Mya in the Chattian Age), although with a wide 95% confidence interval ranging from the Early Oligocene (28.91 Mya in the Rupelian Age) to the Early Miocene. This finding is consistent with previous multi-locus studies [12, 13, 46]. The formation of the Gulf of Muthaymimah in northeastern Arabia in the Early Oligocene [48, 49] likely led to the divergence of *A. montanus* from the other *Asaccus* species. During the Early Miocene (19.28 Mya in the Burdigalian Age), following the final retreat of the Tethys Sea and the collision of the African-Eurasian (Arabian, Iranian, and Anatolian) plates, a gene flow event occurred from Arabian *Asaccus* populations toward northern Zagros [49–54].

In addition, the results of our study revealed divergence times for various *Asaccus* species across the Zagros region and Arabian territories. Population 6 diverged approximately 15.33 Mya, during the Middle Miocene (Langhian Age). Subsequent divergences included *Asaccus* species in northern Zagros, such as *A. kermanshahensis*, *A. kurdistanensis*, *A. griseonotus*, *A. andersoni*, Population 2, and Population 10 around 14.5 Mya. Moreover, the diversification of Arabian *Asaccus* species began at about 13.91 Mya, followed by gene flow from Arabia to southeastern Iran, a region presently known as Makran. This divergence was a consequence of the Arabia-Eurasia collision, which not only formed the Zagros Mountain chain but also created the Gomphotherium land bridge around 19 Mya. This bridge, connecting Eurasia and Africa, enabled the first significant faunal exchanges between these continents, facilitating speciation by dispersal. The collision and the Arabian Peninsula’s anticlockwise rotation led to the uplift of the Zagros Mountains on the Iranian plateau, forming a formidable barrier that impeded further dispersal of the *Asaccus* species. This geological transformation led to the separation of *Asaccus* species in southern Zagros—including *A. iranicus* and **Populations 1, 3, 4, 5**, and **9**—from those in the northern and central Zagros such as *A. elisae*, **Population 8**, and **Population 7**.

### Species boundaries in *Asaccus*

Studies based on morphological [17] or molecular data [12, 13, 21, 47] suggest that the diversity of *Asaccus* species of Arabia is notably higher than currently recognized. Similarly, the present study using both molecular and morphological data highlights the considerable cryptic diversity of *Asaccus* across the Zagros Mountains. Our morphological analyses suggest that most of the Southwest Asian leaf-toed geckos are not morphologically distinguishable and the existing identification keys for the *Asaccus* species are less practical for accurate identification of the members of this complex. Moreover, our findings point to the importance of applying an integrative approach to species delimitation, such as using morphological and molecular data to uncover cryptic species diversity. As observed in previous studies, an integrative approach may increase the reliability and validity of the results [55]. Prior to this study, Fattahi et al. [21]. conducted a pioneering molecular study on the phylogeny of *Asaccus* species using a large number of samples from Iran. However, they identified several phylogenetically unresolved populations and species delimitation analyses within *Asaccus* species were conducted using only phylogenetic and p-distance methods.

Carstens et al. [56]. recommended that a broad range of species delimitation analyses should be applied to data and only consistently congruent delimitation results should be given credence to. Incongruent outputs across methods may be due to variation in the ability of methods to detect cryptic lineages or may be a signature of the assumptions of one or more methods not being observed. Under these conditions, the results should be inferred conservatively as in the majority of cases, failing to identify species is preferred to false delimitation of non-existent evolutionary lineages. To avoid over-splitting, we opted for species that were consistently recognized by all approaches as distinct species and were delimited based on the consensus of five gene-based species delimitation methods (ABGD, GMYC, BPP, bPTP, and mPTP) [57]. The analyses yielded 22 species clusters, eight of which are newly resolved lineages for *Asaccus* (Fig. 6), while the remaining14 species could be assigned to formerly described species, namely *A*. *montanus*, *A*. *margaritae*, *A*. *gallagheri*, *A*. *gardneri*, *A*. *platyrhynchus*, *A*. *caudivolvulus*, *A*. *arnoldi*, *A*. *elisae*, *A*. *andersoni*, *A*. *iranicus*, *A*. *griseonotus*, *A*. *granularis*, *A*. *kermanshahensis*, and *A*. *kurdistanensis*.

*Asaccus* species are known as cryptic species that exhibit high levels of diversity and microendemicity across their distribution range [5, 12, 13, 21, 47]. The results of species genetic delimitation analyses revealed at least five potential new *Asaccus* species from the Zagros Mountains. However, our morphometric analysis did not support these five species to be significantly diverged from each other. Consensus results of the phylogenetic trees, haplotype networks and species delimitation approaches suggest that *A. arnoldi*, *A. gallagheri*, and *A. montanus* might each consist of more than one species. However, as we opted not to update the taxonomic and nomenclatural status of these species, we continued to use the current taxonomic designations of *A. arnoldi*, *A. gallagheri*, and *A. montanus* in this study. Also, due to the lack of morphological data for **Population 6** from Jask, Hormozgan Province, **Population 7** from Masjedsoleyman, Khuzestan Province, **Population 8** from Gilan-e-Gharb, Kermanshah Province, **Population 9** from Bijar, Ilam Province, and **Population 10** from Nosoud, Kurdistan Province and Patagh, Kermanshah Province in the present study, there currently seems to be insufficient morphological evidence to describe these taxa as new species, although our molecular results strongly support recognition of their species status.

Torki et al. [4]. described *A. granularis* from Khersdar Mountain, 5 km NW of Pol-e-Dokhtar, Lorestan province as a new species. In our study, one specimen from the Museum of Vertebrate Zoology (MVZ234326) deposited in GenBank as *A. griseonotus* from 99 km SW of Khorram Abad, Lorestan Province clustered within *A. granularis*, suggesting a possible error in this record. This sample has been used in the present as well as previous studies [12, 13, 21, 47] under the name *A. griseonotus*. A striking result of this study was that *A. granularis* specimens (**Population 2**) collected close to its type locality (NE of Khersdar Mountain, Lorestan Province) represented a separate lineage, which was sister to *A. griseonotus*. Furthermore, *A. elisae* has been previously reported from several localities in Iran [58]. The specimens from these localities have been described morphologically as *A. elisae* but exhibit high genetic divergence in our results as well as in Fattahi et al.’s [21] study. This may suggest that *A. elisae* comprises different species with similar morphological characters. For instance, one distinct lineage from Gilan-e-Gharb, Kermanshah Province (**Population 4**; Figs. 2 and 6) is sister to *A. elisae*, whereas another specimen from Masjedsoleyman, Khuzestan Province (**Population 8**; Fig. 2) is located in a different clade from the type species, *A. elisae*. Four lineages from Bijar in Ilam Province (**Population 9**), Gilan-e-Gharb in Kermanshah Province (**Population 4**), north of Parishan Lake in Fars Province (**Population 3**), and 22 km SE of Gachsaran in Kohgiluyeh and Boyer-Ahmad Province (**Population 5**) (Figs. 2 and 6) were each placed in different clades despite being morphologically similar to *A. elisae*.

The two species of *A. iranicus* and *A. tangestanensis* described by Torki et al. [11]. have until now been recorded from their type localities only. These species formed a single well-supported clade in our phylogenetic trees, haplotype networks and species delimitation analyses, despite being proposed as separate species in Fattahi et al.’s [21] study. Therefore, we synonymized *A. tangestanensis* under *A. iranicus*, given that no nomenclatural priority was involved as they were described in the same paper. In both ML and BI reconstructions, *A. griseonotus* and *A. nasrullahi* were recovered as a single lineage strongly supported by high BI PP (1.00) and ML bootstrap values (97), a finding also confirmed by our species delimitation results. Following nomenclatural priorities, *A. nasrullahi* was synonymized under *A. griseonotus.* Also, *A. zagrosicus* which was recently described from south of Iran [11] was found to be closely related to *A. elisae*. Although *A. zagrosicus* is diagnosably different from *A. elisae* in having secondary postmentals separated from lower labials by 1–3 granules, it was nested within *A. elisae* (Fig. 2) with high support in the phylogenetic trees, species delimitation results and haplotype networks. Therefore, we synonymized *A*. *zagrosicus* under its sister taxon, *A*. *elisae*. Moreover, a geographically distant and isolated population of *Asaccus* found near Jask (**Population 6**), a port town in SE of Iran along the Sea of Oman, was separated into a well-diverged lineage with high BI PP (1.00) and ML bootstrap (97) support values. Finally, another taxon found in southern Zagros from a small cave near NourAbad Mamasani, Fars Province (**Population 1**), diverged from **Population 5** with high BI PP (0.99) and ML bootstrap (98) support values.

### Updating the distribution of Southwest Asian leaf-toed geckos in Iran

We conducted a comprehensive mass sampling of *Asaccus* species in Iran, which enabled us to obtain more reliable data on their distribution. Given that a number of *Asaccus* species have only been recorded from their type localities, the results of the present study and those of Fattahi et al. [21]. could help update the distribution map of recently discovered endemic species. *A*. *kurdistanensis* was collected by Rastegar-Pouyani on 13 June 2004 at ca. 1,850 m above sea level in the Zagros Mountains, 10 km NW of Sarvabad in Kurdistan Province, western Iran, while in the present study and Fattahi et al.’s study [21], it was collected from Palangan, Uraman Takht in Kurdistan Province (Fig. 4). *A. kermanshahensis* was only known from a small cave along the Mianrahan road in Kermanshah Province when described in 2017. Here, we confirmed its formerly unknown presence in other adjacent caves. In addition, this species was collected by Fattahi et al. [21]. from 35 km NE of Kermanshah city. They also collected one specimen from 99 km SW of Khorram Abad, Lorestan Province, and three from Farhad Abad to Darreshahr, which suggests this species likely has a broader geographical range than previously documented. Based on the distinctiveness of *A*. *elisae* from the morphologically similar new species described herein, it could be postulated that *A*. *elisae* might be less widely distributed than previously thought. Further, judging by the many *A*. *granularis* specimens collected by Fattahi et al. [21]. , this species appears to be more widely distributed than currently known. Similarly, as *A*. *griseonotus* was collected from several new localities and was synonymized with *A*. *nasrullahi*, we suggest that *A*. *griseonotus* is more widely distributed than formerly recognized. Finally, as *A*. *iranicus* and *A*. *tangestanensis* were genetically resolved as conspecific and thus *A*. *tangestanensis* was synonymized under *A*. *iranicus*, and considering the many samples we collected of these taxa, we suggest their distribution in southern Iran is wider than previously thought.

### Conservation units and management propositions

For decades, conservationists have debated over the delineation of the basic units of conservation to prioritize management actions [59–61]. The ongoing ambiguity surrounding the definition and delimitation of conservation units at the species or subspecies levels has spurred a great deal of controversy among the scientific community. In their attempt to eliminate this confusion, scientists coined the term evolutionarily significant unit (ESU), aka evolutionary significant unit, which represents intraspecific groups of taxa that merit conservation attention given their evolutionary originality [62, 63]. Although no significant consensus has yet emerged on the definition of ESUs, we leaned toward the definition provided by Fraser & Bernatchez [61] as it places greater focus on conservation of isolated populations. ESUs are generally defined as lineages that pursued independent evolutionary paths and are characterized by highly restricted gene flow with other lineages, making each isolated population of *Asaccus* a fitting ESU for conservation purposes. Here, we proposed 23 ESUs, of which 9 are distributed in the Hajar Mountains of Arabia and 14 in the Zagros Mountains, including two species from Iraq and Turkey that were not targeted in this research.

The International Union for Conservation of Nature (IUCN) has classified *A. caudivolvulus* from the Hajar Mountains of Oman as Critically Endangered, six species from the Hajar Mountains including *A*. *arnoldi*, *A*. *gardneri*, *A*. g*allagheri*, *A*. *margaritae*, *A*. *montanus*, and *A*. *platyrhynchus* and five species from the Zagros Mountains, *A*. *elisae*, *A*. *griseonotus*, *A*. *kermanshahensis*, *A*. *kurdistanensis*, and *A*. *nasrullahi* (herein *A*. *nasrullahi* synonymized with *A*. *griseonotus*) as Least Concern. Yet, the conservation status of other species within the genus has received little consideration, despite the enormous impact of threatening factors such as mining, urban development, and land use change on *Asaccus* populations. In Iran, all *Asaccus* species remain officially unprotected under DOEI laws. In the present research, we showed that 12 out of 21 *Asaccus* species or populations are endemic to Iran; thus, we highly recommend the DOEI to (i) revise the boundaries of protected areas across the distribution range of endemic Southwest Asian leaf-toed geckos in Iran; (ii) renew the list of protected species according to the IUCN criteria to include all those considered worthy of conservation.

### Electronic supplementary material

Below is the link to the electronic supplementary material.

Supplementary Material 1

Supplementary Material 2

Supplementary Material 3

Supplementary Material 4

Supplementary Material 5

## Data Availability

The datasets generated during the current study are available from the corresponding author on reasonable request. All sequence data are deposited in NCBI under accession codes OQ401704-OQ401804.
