# Supplementary material for: Integrating phylogenetic, phylogeographic, and morphometric analyses to reveal cryptic lineages within the genus Asaccus (Reptilia: Squamata: Phyllodactylidae) in Iran
Source: BMC Zool. 2024 Jun 26;9:12. doi: 10.1186/s40850-024-00203-1 (PMC11202258; doi:10.1186/s40850-024-00203-1)
Supplement: Supplementary file 1 — Supplementary Material 1 [file 40850_2024_203_MOESM1_ESM.docx]

**Table S1.** Specimens used in the phylogenetic and morphological analyses, voucher codes, locality data, and GenBank accession numbers.

| **Ingroup species** | **Synonymized sp.** | **Voucher code** | **Elevation (m)** | **Locality** | **Latitude** | **Longitude** | **Morphology** | **12S** | **Cyt *b*** | ***c-mos*** |
| --- | --- | --- | --- | --- | --- | --- | --- | --- | --- | --- |
| *A. iranicus*** | *A. tangestanensis* | NEZMUT1421 | 823 | Fars (Iran) | 27.897 | 53.704 | yes | PP866317 |  | OQ401704 |
| *A. iranicus*** | *A. tangestanensis* | NEZMUT1422 | 823 | Fars (Iran) | 27.897 | 53.704 | yes | PP866318 |  | OQ401705 |
| *A. iranicus*** | *A. tangestanensis* | NEZMUT1423 | 823 | Fars (Iran) | 27.897 | 53.704 | yes | PP866319 |  | OQ401706 |
| *A. iranicus*** | *A. tangestanensis* | NEZMUT1424 | 823 | Fars (Iran) | 27.897 | 53.704 | yes | PP866320 |  | OQ401707 |
| *A. iranicus** |  | NEZMUT1359 | 19 | Hormozgan (Iran) | 27.349 | 52.832 | yes |  |  |  |
| *A. iranicus***T |  | NEZMUT1360 | 19 | Hormozgan (Iran) | 27.349 | 52.832 | yes | PP866321 |  | OQ401708 |
| *A. iranicus***T |  | NEZMUT1361 | 19 | Hormozgan (Iran) | 27.349 | 52.832 | yes | PP866322 |  | OQ401709 |
| *A. iranicus**T |  | NEZMUT1362 | 19 | Hormozgan (Iran) | 27.349 | 52.832 | yes |  |  |  |
| *A. iranicus***T |  | NEZMUT1363 | 19 | Hormozgan (Iran) | 27.349 | 52.832 | yes | PP866323 |  | OQ401710 |
| *A. iranicus** | *A. tangestanensis* | NEZMUT1364 | 362 | Bushehr (Iran) | 28.061 | 51.991 | yes |  |  |  |
| *A. iranicus** | *A. tangestanensis* | NEZMUT1365 | 362 | Bushehr (Iran) | 28.061 | 51.991 | yes |  |  |  |
| *A. iranicus** | *A. tangestanensis* | NEZMUT1366 | 362 | Bushehr (Iran) | 28.061 | 51.991 | yes |  |  |  |
| *A. iranicus** | *A. tangestanensis* | NEZMUT1367 | 362 | Bushehr (Iran) | 28.061 | 51.991 | yes |  |  |  |
| *A. iranicus** | *A. tangestanensis* | NEZMUT1368 | 362 | Bushehr (Iran) | 28.061 | 51.991 | yes |  |  |  |
| *A. iranicus** | *A. tangestanensis* | NEZMUT1369 | 362 | Bushehr (Iran) | 28.061 | 51.991 | yes |  |  |  |
| *A. iranicus** | *A. tangestanensis* | NEZMUT1370 | 362 | Bushehr (Iran) | 28.061 | 51.991 | yes |  |  |  |
| *A. iranicus*** | *A. tangestanensis* | NEZMUT1371 | 362 | Bushehr (Iran) | 28.061 | 51.991 | yes | PP866324 |  | OQ401711 |
| *A. iranicus*** | *A. tangestanensis* | NEZMUT1372 | 362 | Bushehr (Iran) | 28.061 | 51.991 | yes | PP866325 |  | OQ401712 |
| *A. iranicus***T | *A. tangestanensis* | NEZMUT1373 | 494 | Bushehr (Iran) | 28.729 | 51.518 | yes | PP866326 |  | OQ401713 |
| *A. iranicus**T | *A. tangestanensis* | NEZMUT1374 | 494 | Bushehr (Iran) | 28.729 | 51.518 | yes |  |  |  |
| *A. iranicus**T | *A. tangestanensis* | NEZMUT1375 | 494 | Bushehr (Iran) | 28.729 | 51.518 | yes |  |  |  |
| *A. iranicus***T | *A. tangestanensis* | NEZMUT1376 | 494 | Bushehr (Iran) | 28.729 | 51.518 | yes | PP866327 |  | OQ401714 |
| *A. iranicus***T | *A. tangestanensis* | NEZMUT1377 | 494 | Bushehr (Iran) | 28.729 | 51.518 | yes | PP866328 |  | OQ401715 |
| *A. iranicus**T | *A. tangestanensis* | NEZMUT1378 | 494 | Bushehr (Iran) | 28.729 | 51.518 | yes |  |  |  |
| *A. iranicus**T | *A. tangestanensis* | NEZMUT1379 | 494 | Bushehr (Iran) | 28.729 | 51.518 | yes |  |  |  |
| *A. iranicus* |  | ERP 1755 |  | Fars (Iran) | 27.53 | 52.85 |  | MT828072 | MT828166 |  |
| *A. iranicus* |  | ERP 1756 |  | Fars (Iran) | 27.53 | 52.85 |  | MT828073 | MT828167 |  |
| *A. iranicus* |  | ERP 1757 |  | Fars (Iran) | 27.53 | 52.85 |  | MT828074 | MT828168 |  |
| *A. iranicus* | *A. tangestanensis* | ERP 3601 |  | Bushehr (Iran) | 28.57 | 51.22 |  | MT828098 | MT828192 |  |
| *A. iranicus* | *A. tangestanensis* | ERP 3602 |  | Bushehr (Iran) | 28.57 | 51.22 |  | MT828099 | MT828193 |  |
| *A. iranicus* | *A. tangestanensis* | ERP 3604 |  | Bushehr (Iran) | 28.57 | 51.22 |  | MT828100 | MT828194 |  |
| *A. iranicus* | *A. tangestanensis* | ERP 3609 |  | Bushehr (Iran) | 27.21 | 52.38 |  | MT828106 | MT828200 |  |
| *A. iranicus* | *A. tangestanensis* | ERP 3610 |  | Bushehr (Iran) | 27.21 | 52.38 |  | MT828107 | MT828201 |  |
| *A. iranicus* | *A. tangestanensis* | ERP 3611 |  | Bushehr (Iran) | 27.21 | 52.38 |  | MT828108 | MT828202 |  |
| *A. iranicus* | *A. tangestanensis* | ERP 3612 |  | Bushehr (Iran) | 27.21 | 52.38 |  | MT828109 | MT828203 |  |
| *A. iranicus* | *A. tangestanensis* | ERP 3613 |  | Bushehr (Iran) | 27.21 | 52.38 |  | MT828110 | MT828204 |  |
| *A. iranicus* | *A. tangestanensis* | ERP 3614 |  | Bushehr (Iran) | 27.21 | 52.38 |  | MT828111 | MT828205 |  |
| *A. iranicus* | *A. tangestanensis* | ERP 1215 |  | Bushehr (Iran) | 28.81 | 51.38 |  | MT828126 | MT828220 |  |
| *A. iranicus* |  | ERP 5562 |  | Fars (Iran) | 27.38 | 53 |  | MT828081 | MT828175 |  |
| *A. iranicus* |  | ERP 5573 |  | Fars (Iran) | 27.38 | 53 |  | MT828082 | MT828176 |  |
| *A. iranicus* |  | ERP 5574 |  | Fars (Iran) | 27.38 | 53 |  | MT828083 | MT828177 |  |
| *A. iranicus* | *A. tangestanensis* | ERP 3631 |  | Bushehr (Iran) | 28.48 | 51.36 |  | MT828101 | MT828195 |  |
| *A. iranicus* | *A. tangestanensis* | ERP 3632 |  | Bushehr (Iran) | 28.48 | 51.36 |  | MT828102 | MT828196 |  |
| *A. iranicus* | *A. tangestanensis* | ERP 3633 |  | Bushehr (Iran) | 28.48 | 51.36 |  | MT828103 | MT828197 |  |
| *A. iranicus* | *A. tangestanensis* | ERP 3634 |  | Bushehr (Iran) | 28.48 | 51.36 |  | MT828104 | MT828198 |  |
| *A. iranicus* | *A. tangestanensis* | ERP 3635 |  | Bushehr (Iran) | 28.48 | 51.36 |  | MT828105 | MT828199 |  |
| *A. kermanshahensis* T |  | NEZMUT1350 | 157 | Kermanshah (Iran) | 34.589 | 47.448 | yes |  |  |  |
| *A. kermanshahensis* T |  | NEZMUT1351 | 760 | Kermanshah (Iran) | 34.589 | 47.448 | yes |  |  |  |
| *A. kermanshahensis*** T |  | NEZMUT1475 | 157 | Kermanshah (Iran) | 34.589 | 47.448 | yes | PP866329 | PP883785 | OQ401716 |
| *A. kermanshahensis* T |  | NEZMUT1476 | 280 | Kermanshah (Iran) | 34.589 | 47.448 | yes |  |  |  |
| *A. kermanshahensis* T |  | NEZMUT1477 | 256 | Kermanshah (Iran) | 34.589 | 47.448 | yes |  |  |  |
| *A. kermanshahensis* T |  | NEZMUT1478 | 157 | Kermanshah (Iran) | 34.589 | 47.448 | yes |  |  |  |
| *A. kermanshahensis* T |  | NEZMUT1479 | 280 | Kermanshah (Iran) | 34.589 | 47.448 | yes |  |  |  |
| *A. kermanshahensis***T |  | NEZMUT1480 | 280 | Kermanshah (Iran) | 34.589 | 47.448 | yes | PP866330 |  | OQ401717 |
| *A. kermanshahensis* |  | 17c |  | Kermanshah (Iran) | 34.543 | 47.106 |  | MT828033 | MT828130 |  |
| *A. kermanshahensis* |  | 37b |  | Kermanshah (Iran) | 34.543 | 47.106 |  | MT828034 | MT828131 |  |
| *A. kurdistanensis**T |  | NEZMUT1495 | 280 | Kurdistan (Iran) | 35.409 | 46.262 | yes |  |  |  |
| *A. kurdistanensis**T |  | NEZMUT1496 | 200 | Kurdistan (Iran) | 35.409 | 46.262 | yes |  |  |  |
| *A. kurdistanensis**T |  | NEZMUT1497 | 280 | Kurdistan (Iran) | 35.409 | 46.262 | yes |  |  |  |
| *A. kurdistanensis**T |  | NEZMUT1498 | 664 | Kurdistan (Iran) | 35.409 | 46.262 | yes |  |  |  |
| *A. kurdistanensis**T |  | NEZMUT1499 | 921 | Kurdistan (Iran) | 35.409 | 46.262 | yes |  |  |  |
| *A. kurdistanensis** |  | NEZMUT1339 | 1422 | Kurdistan (Iran) | 35.253 | 46.261 | yes |  |  |  |
| *A. kurdistanensis*** |  | NEZMUT1340 | 1422 | Kurdistan (Iran) | 35.253 | 46.261 | yes | PP866331 |  | OQ401718 |
| *A. kurdistanensis*** |  | NEZMUT1341 | 1422 | Kurdistan (Iran) | 35.253 | 46.261 | yes | PP866332 |  | OQ401719 |
| *A. kurdistanensis*** |  | NEZMUT1342 | 1422 | Kurdistan (Iran) | 35.253 | 46.263 | yes | PP866333 |  | OQ401720 |
| *A. kurdistanensis** |  | NEZMUT1501 | 1422 | Kurdistan (Iran) | 35.253 | 46.263 | yes |  |  |  |
| *A. kurdistanensis** |  | NEZMUT1502 | 1422 | Kurdistan (Iran) | 35.253 | 46.263 | yes |  |  |  |
| *A. kurdistanensis** |  | NEZMUT1503 | 1422 | Kurdistan (Iran) | 35.253 | 46.261 | yes |  |  |  |
| *A. kurdistanensis** |  | NEZMUT1504 | 1422 | Kurdistan (Iran) | 35.253 | 46.261 | yes |  |  |  |
| *A. kurdistanensis** |  | NEZMUT1505 | 1422 | Kurdistan (Iran) | 35.253 | 46.261 | yes |  |  |  |
| *A. kurdistanensis** |  | NEZMUT1506 | 1422 | Kurdistan (Iran) | 35.253 | 46.261 | yes |  |  |  |
| *A. kurdistanensis** |  | NEZMUT1343 |  | Kurdistan (Iran) | 35.06 | 46.6 | yes |  |  |  |
| *A. kurdistanensis** |  | NEZMUT1344 |  | Kurdistan (Iran) | 35.06 | 46.6 | yes |  |  |  |
| *A. kurdistanensis** |  | NEZMUT1345 |  | Kurdistan (Iran) | 35.06 | 46.6 | yes |  |  |  |
| *A. kurdistanensis** |  | NEZMUT1346 |  | Kurdistan (Iran) | 35.06 | 46.6 | yes |  |  |  |
| *A. kurdistanensis** |  | NEZMUT1347 |  | Kurdistan (Iran) | 35.06 | 46.6 | yes |  |  |  |
| *A. kurdistanensis* |  | ERP 2403 |  | Kurdistan (Iran) | 35.06 | 46.6 |  | MT828122 | MT828216 |  |
| *A. kurdistanensis* |  | ERP 8665 |  | Kurdistan (Iran) | 35.06 | 46.6 |  | MT828090 | MT828184 |  |
| *A. elisae***T | *A. zagrosicus* | NEZMUT1452 | 746 | Khuzestan (Iran) | 33.038 | 48.667 | yes | PP866334 |  | OQ401721 |
| *A. elisae**T | *A. zagrosicus* | NEZMUT1453 | 746 | Khuzestan (Iran) | 33.038 | 48.667 | yes |  |  |  |
| *A. elisae**T | *A. zagrosicus* | NEZMUT1454 | 746 | Khuzestan (Iran) | 33.038 | 48.667 | yes |  |  |  |
| *A. elisae***T | *A. zagrosicus* | NEZMUT1455 | 746 | Khuzestan (Iran) | 33.038 | 48.667 | yes | PP866335 |  | OQ401722 |
| *A. elisae***T | *A. zagrosicus* | NEZMUT1456 | 746 | Khuzestan (Iran) | 33.038 | 48.667 | yes | PP866336 |  | OQ401723 |
| *A. elisae* | *A. zagrosicus* | ERP 2960 |  | Kermanshah (Iran) | 34.08 | 46.02 |  | MT828118 | MT828212 |  |
| *A. elisae* T | *A. zagrosicus* | ERP 6547 |  | Khuzestan (Iran) | 33.03 | 48.65 |  | MT828127 | MT828221 |  |
| *A. elisae* T | *A. zagrosicus* | ERP 6549 |  | Khuzestan (Iran) | 33.03 | 48.65 |  | MT828128 | MT828222 |  |
| *A. elisae* T | *A. zagrosicus* | ERP 6555 |  | Khuzestan (Iran) | 33.03 | 48.65 |  | MT828129 | MT828223 |  |
| *A. granularis* |  | ERP 2413 |  | Ilam (Iran) | 35.11 | 47.36 |  | MT828065 | MT828159 |  |
| *A. granularis* |  | ERP 2418 |  | Ilam (Iran) | 35.11 | 47.36 |  | MT828066 | MT828160 |  |
| *A. granularis* |  | ERP 6535 |  | Lorestan (Iran) | 33.198 | 47.707 |  | MT828075 | MT828169 |  |
| *A. granularis* |  | ERP 6536 |  | Lorestan (Iran) | 33.198 | 47.707 |  | MT828076 | MT828170 |  |
| *A. granularis* |  | ERP 6537 |  | Lorestan (Iran) | 33.198 | 47.707 |  | MT828077 | MT828171 |  |
| *A. granularis* |  | ERP 6538 |  | Lorestan (Iran) | 33.198 | 47.707 |  | MT828078 | MT828172 |  |
| *A. granularis* |  | ERP 6539 |  | Lorestan (Iran) | 33.198 | 47.707 |  | MT828079 | MT828173 |  |
| *A. granularis* |  | ERP 6541 |  | Lorestan (Iran) | 33.198 | 47.707 |  | MT828080 | MT828174 |  |
| *A. granularis* |  | ERP 7515 |  | Ilam (Iran) | 35.11 | 47.36 |  | MT828115 | MT828209 |  |
| *A. granularis* |  | ERP 7516 |  | Ilam (Iran) | 35.11 | 47.36 |  | MT828116 | MT828210 |  |
| *A. granularis* |  | ERP 7523 |  | Ilam (Iran) | 35.11 | 47.36 |  | MT828117 | MT828211 |  |
| *A. griseonotus**T | *A. nasrullahi* | NEZMUT1446 | 711 | Khuzestan (Iran) | 32.845 | 48.262 | yes |  |  |  |
| *A. griseonotus***T | *A. nasrullahi* | NEZMUT1447 | 711 | Khuzestan (Iran) | 32.845 | 48.262 | yes | PP866337 |  | OQ401724 |
| *A. griseonotus***T | *A. nasrullahi* | NEZMUT1448 | 711 | Khuzestan (Iran) | 32.845 | 48.262 | yes | PP866338 |  | OQ401725 |
| *A. griseonotus**T | *A. nasrullahi* | NEZMUT1449 | 711 | Khuzestan (Iran) | 32.845 | 48.262 | yes |  |  |  |
| *A. griseonotus***T | *A. nasrullahi* | NEZMUT1450 | 711 | Khuzestan (Iran) | 32.845 | 48.262 | yes | PP866339 | PP883784 | OQ401726 |
| *A. griseonotus**T | *A. nasrullahi* | NEZMUT1451 | 711 | Khuzestan (Iran) | 32.845 | 48.262 | yes |  |  |  |
| *A. griseonotus*** |  | NEZMUT1463 | 638 | Lorestan (Iran) | 33.259 | 47.804 | yes | PP866340 | PP883782 | OQ401727 |
| *A. griseonotus*** |  | NEZMUT1464 | 638 | Lorestan (Iran) | 33.259 | 47.804 | yes | PP866341 |  | OQ401728 |
| *A. griseonotus*** |  | NEZMUT1465 | 638 | Lorestan (Iran) | 33.259 | 47.804 | yes | PP866342 |  | OQ401729 |
| *A. griseonotus*** |  | NEZMUT1466 | 638 | Lorestan (Iran) | 33.259 | 47.804 | yes | PP866343 | PP883783 | OQ401730 |
| *A. griseonotus** |  | NEZMUT1467 | 638 | Lorestan (Iran) | 33.259 | 47.804 | yes |  |  |  |
| *A. griseonotus** |  | NEZMUT1507 | 638 | Lorestan (Iran) | 33.259 | 47.804 | yes |  |  |  |
| *A. griseonotus** |  | NEZMUT1508 | 638 | Lorestan (Iran) | 33.259 | 47.804 | yes |  |  |  |
| *A. griseonotus** |  | NEZMUT1483 | 724 | Ilam (Iran) | 33.0822 | 47.451 | yes |  |  |  |
| *A. griseonotus*** |  | NEZMUT1484 | 724 | Ilam (Iran) | 33.0822 | 47.451 | yes | PP866344 |  | OQ401731 |
| *A. griseonotus** |  | NEZMUT1485 | 724 | Ilam (Iran) | 33.0822 | 47.451 | yes |  |  |  |
| *A. griseonotus*** |  | NEZMUT1486 | 724 | Ilam (Iran) | 33.0822 | 47.451 | yes | PP866345 |  | OQ401732 |
| *A. griseonotus** |  | NEZMUT1487 | 724 | Ilam (Iran) | 33.0822 | 47.451 | yes |  |  |  |
| *A. griseonotus*** |  | NEZMUT1488 | 724 | Ilam (Iran) | 33.0822 | 47.451 | yes | PP866346 |  | OQ401733 |
| *A. griseonotus** |  | NEZMUT1489 | 724 | Ilam (Iran) | 33.0822 | 47.451 | yes |  |  |  |
| *A. andersoni***T |  | NEZMUT1418 | 1221 | Ilam (Iran) | 33.967 | 46.145 | yes | PP866347 |  | OQ401734 |
| *A. andersoni**T |  | NEZMUT1419 | 1221 | Ilam (Iran) | 33.967 | 46.145 | yes |  |  |  |
| *A. andersoni***T |  | NEZMUT1420 | 1221 | Ilam (Iran) | 33.967 | 46.145 | yes | PP866348 |  | OQ401735 |
| *A. andersoni**T |  | NEZMUT1473 | 1221 | Ilam (Iran) | 33.967 | 46.145 | yes |  |  |  |
| *A. andersoni***T |  | NEZMUT1474 | 1221 | Ilam (Iran) | 33.967 | 46.145 | yes | PP866349 |  | OQ401736 |
| **Population 7** |  | ERP 3599 |  | Khuzestan (Iran) | 32.03 | 49.16 |  | MT828067 | MT828161 |  |
| **Population 7** |  | ERP 3600 |  | Khuzestan (Iran) | 32.03 | 49.16 |  | MT828068 | MT828162 |  |
| **Population 6** |  | ERP 8428 |  | Hormozgan (Iran) | 25.82 | 57.78 |  | MT828095 | MT828189 |  |
| **Population 6** |  | ERP 8429 |  | Hormozgan (Iran) | 25.82 | 57.78 |  | MT828096 | MT828190 |  |
| **Population 6** |  | ERP 8430 |  | Hormozgan (Iran) | 25.82 | 57.78 |  | MT828097 | MT828191 |  |
| **Population 4** **** |  | NEZMUT1471 | 968 | Kermanshah (Iran) | 34.107 | 45.999 | yes | PP866350 |  | OQ401737 |
| **Population 4** **** |  | NEZMUT1472 | 968 | Kermanshah (Iran) | 34.107 | 45.999 | yes | PP866351 |  | OQ401738 |
| **Population 4** |  | As. 1 |  | Kermanshah (Iran) | 34.268 | 45.761 |  | MT828035 | MT828132 |  |
| **Population 4** |  | As. 2 |  | Kermanshah (Iran) | 34.268 | 45.761 |  | MT828036 | MT828133 |  |
| **Population 4** |  | As. 3 |  | Lorestan (Iran) | 34.268 | 45.761 |  | MT828037 | MT828134 |  |
| **Population 2** **** |  | NEZMUT1468 | 774 | Lorestan (Iran) | 33.219 | 47.6735 | yes | PP866352 |  | OQ401739 |
| **Population 2** **** |  | NEZMUT1469 | 774 | Lorestan (Iran) | 33.219 | 47.6735 | yes | PP866353 |  | OQ401740 |
| **Population 2 **** |  | NEZMUT1470 | 774 | Lorestan (Iran) | 33.219 | 47.6735 | yes | PP866354 |  | OQ401741 |
| **Population 10** |  | ERP 8669 |  | Kermanshah (Iran) | 34.435 | 45.995 |  | MT828057 |  |  |
| **Population 10** |  | ERP 2411 |  | Kurdistan (Iran) | 35.11 | 46.25 |  | MT828123 | MT828217 |  |
| **Population 10** |  | ERP 2412 |  | Kurdistan (Iran) | 35.11 | 46.25 |  | MT828124 | MT828218 |  |
| **Population 3 **** |  | NEZMUT1429 | 851 | Fars (Iran) | 29.545 | 51.783 | yes | PP866355 |  | OQ401742 |
| **Population 3** **** |  | NEZMUT1430 | 851 | Fars (Iran) | 29.545 | 51.783 | yes | PP866356 |  | OQ401743 |
| **Population 3** **** |  | NEZMUT1431 | 851 | Fars (Iran) | 29.545 | 51.783 | yes | PP866357 |  | OQ401744 |
| **Population 3 **** |  | NEZMUT1432 | 851 | Fars (Iran) | 29.545 | 51.783 | yes | PP866358 |  | OQ401745 |
| **Population 3 *** |  | NEZMUT1433 | 851 | Fars (Iran) | 29.545 | 51.783 | yes |  |  |  |
| **Population 3 *** |  | ERP 1423 |  | Fars (Iran) | 29.53 | 51.78 |  | MT828064 | MT828158 |  |
| **Population 1*** |  | NEZMUT1352 | 1025 | Fars (Iran) | 30.069 | 51.554 | yes |  |  |  |
| **Population 1**** |  | NEZMUT1353 | 1025 | Fars (Iran) | 30.069 | 51.554 | yes | PP866359 |  | OQ401746 |
| **Population 1 **** |  | NEZMUT1354 | 1025 | Fars (Iran) | 30.069 | 51.554 | yes | PP866360 |  | OQ401747 |
| **Population 1**** |  | NEZMUT1426 | 1025 | Fars (Iran) | 30.069 | 51.554 | yes | PP866361 |  | OQ401748 |
| **Population 1 **** |  | NEZMUT1427 | 1025 | Fars (Iran) | 30.069 | 51.554 | yes | PP866362 |  | OQ401749 |
| **Population 1 **** |  | NEZMUT1428 | 1025 | Fars (Iran) | 30.069 | 51.554 | yes | PP866363 |  | OQ401750 |
| **Population 5 *** |  | NEZMUT1434 | 719 | Kohgiluyeh Boyerahmad (Iran) | 30.316 | 50.983 | yes |  |  |  |
| **Population 5 *** |  | NEZMUT1435 | 615 | Kohgiluyeh Boyerahmad (Iran) | 30.316 | 50.983 | yes |  |  |  |
| **Population 5 *** |  | NEZMUT1436 | 719 | Kohgiluyeh Boyerahmad (Iran) | 30.316 | 50.983 | yes |  |  |  |
| **Population 5 *** |  | NEZMUT1437 | 719 | Kohgiluyeh Boyerahmad (Iran) | 30.316 | 50.983 | yes |  |  |  |
| **Population 5 *** |  | NEZMUT1438 | 719 | Kohgiluyeh Boyerahmad (Iran) | 30.316 | 50.983 | yes |  |  |  |
| **Population 5 *** |  | NEZMUT1439 | 719 | Kohgiluyeh Boyerahmad (Iran) | 30.316 | 50.983 | yes |  |  |  |
| **Population 5 *** |  | NEZMUT1440 | 719 | Kohgiluyeh Boyerahmad (Iran) | 30.316 | 50.983 | yes | PP866364 |  | OQ401751 |
| **Population 9** |  | ERP 4007 |  | Ilam (Iran) | 33.39 | 45.59 |  | MT828069 | MT828163 |  |
| **Population 9** |  | ERP 4009 |  | Ilam (Iran) | 33.39 | 45.59 |  | MT828070 | MT828164 |  |
| **Population 9** |  | ERP 4010 |  | Ilam (Iran) | 33.39 | 45.59 |  | MT828071 | MT828165 |  |
| **Population 8** |  | As. 20 |  | Kermanshah (Iran) |  |  |  | MT828051 | MT828148 |  |
| **Population 8** |  | As. 21 |  | Kermanshah (Iran) |  |  |  | MT828052 | MT828149 |  |
| **Population 8** |  | As. 22 |  | Kermanshah (Iran) |  |  |  | MT828053 | MT828150 |  |
| *A. arnoldi* |  | IBES7224 |  | Oman | 23.132 | 58.619 | yes | MG019481 | MG019706 | MG019556 |
| *A. arnoldi* |  | IBECN416 |  | Oman | 23.219 | 58.820 | yes | MG019483 | MG019704 | MG019518 |
| *A. arnoldi* |  | IBECN3190 |  | Oman | 23.085 | 58.872 | yes | MG019484 | MG019705 | MG019521 |
| *A. arnoldi* |  | ONHM4234 |  | Oman | 22.616 | 59.094 | yes | MG019492 | MG019709 | MG019555 |
| *A. arnoldi* |  | BMNH2008.962 |  | Oman | 22.616 | 59.094 | yes | MG019480 | MG019708 | MG019553 |
| *A. arnoldi* |  | BMNH2008.961 |  | Oman | 22.616 | 59.094 | yes | MG019482 | MG019710 | MG019554 |
| *A. arnoldi* |  | IBES7576 |  | Oman | 22.616 | 59.094 | yes | MG019479 | MG019707 | MG019552 |
| *A. arnoldi* |  |  |  | Oman | 22.182 | 59.318 |  | MG019485 | MG019713 | MG019558 |
| *A. arnoldi* |  | IBECN182 |  | Oman | 22.169 | 59.414 | yes | MG019490 | MG019712 | MG019523 |
| *A. arnoldi* |  | IBECN4013 |  | Oman | 22.107 | 59.357 | yes | MG019489 | MG019716 | MG019525 |
| *A. arnoldi* |  | IBECN4043 |  | Oman | 22.107 | 59.357 | yes | MG019488 | MG019715 | MG019524 |
| *A. arnoldi* |  | IBECN4264 |  | Oman | 22.107 | 59.357 | yes | MG019487 | MG019711 | MG019519 |
| *A. arnoldi* |  | IBECN4356 |  | Oman | 22.107 | 59.357 | yes | MG019486 | MG019714 | MG019522 |
| *A. arnoldi* |  | IBECN4310 |  | Oman | 23.132 | 58.619 | yes | KX550526 | KX550534 | KX550707 |
| *A. caudivolvulus* |  | IBES7445 |  | UAE | 25.502 | 56.36 | yes | KX550491 | KX550584 | KX550758 |
| *A. caudivolvulus* |  | IBES7866 |  | UAE | 25.502 | 56.36 | yes | KX550492 | KX550585 | KX550759 |
| *A. caudivolvulus* |  | IBES8088 |  | UAE | 25.502 | 56.36 | yes | KX550493 | KX550586 | KX550760 |
| *A. caudivolvulus* |  |  |  | UAE | 25.502 | 56.36 | yes | KX550494 | KX550587 | KX550761 |
| *A. caudivolvulus* |  |  |  | UAE | 25.502 | 56.36 | yes | KX550495 | KX550588 | KX550762 |
| *A. gallagheri* |  | IBECN8294 |  | Oman | 25.502 | 56.36 | yes | MG019437 | MG019690 | MG019569 |
| *A. gallagheri* |  | IBECN5815 |  | Oman | 26.150 | 56.162 | yes | MG019469 | MG019691 | MG019526 |
| *A. gallagheri* |  | IBECN5844 |  | Oman | 26.149 | 56.162 | yes | MG019468 | MG019692 | MG019527 |
| *A. gallagheri* |  |  |  | Oman | 26.099 | 56.329 |  | MG019434 | MG019693 | MG019565 |
| *A. gallagheri* |  |  |  | Oman | 25.979 | 56.205 |  | MG019433 | MG019696 | MG019514 |
| *A. gallagheri* |  | IBECN7945 |  | Oman | 25.978 | 56.205 | yes | MG019425 | MG019675 | MG019568 |
| *A. gallagheri* |  |  |  | Oman | 25.966 | 56.198 |  | MG019428 | MG019683 | MG019564 |
| *A. gallagheri* |  |  |  | Oman | 25.952 | 56.240 |  | MG019423 | MG019688 | MG019501 |
| *A. gallagheri* |  |  |  | Oman | 25.943 | 56.236 |  | MG019436 | MG019694 | MG019515 |
| *A. gallagheri* |  |  |  | Oman | 25.783 | 56.213 |  | MG019417 | MG019685 | MG019516 |
| *A. gallagheri* |  |  |  | Oman | 25.783 | 56.214 |  | MG019427 | MG019686 | MG019500 |
| *A. gallagheri* |  | TW1026 |  | UAE | 25.614 | 56.030 | yes | MG019432 | MG019681 | MG019559 |
| *A. gallagheri* |  | TW1027 |  | UAE | 25.614 | 56.030 | yes | MG019431 | MG019682 | MG019560 |
| *A. gallagheri* |  | IBES7734 |  | UAE | 25.478 | 56.362 | yes | MG019462 | MG019699 | MG019557 |
| *A. gallagheri* |  | IBECN7074 |  | UAE | 25.460 | 56.181 | yes | MG019416 | MG019678 | MG019510 |
| *A. gallagheri* |  | IBECN204 |  | UAE | 25.459 | 56.184 | yes | MG019418 | MG019676 | MG019495 |
| *A. gallagheri* |  | IBECN4179 |  | UAE | 25.459 | 56.184 | yes | MG019419 | MG019679 | MG019503 |
| *A. gallagheri* |  |  |  | UAE | 25.459 | 56.184 |  | MG019426 | MG019677 | MG019512 |
| *A. gallagheri* |  |  |  | UAE | 25.459 | 56.184 |  | MG019422 | MG019684 | MG019504 |
| *A. gallagheri* |  | IBECN8036 |  | UAE | 25.459 | 56.184 | yes | MG019429 | MG019687 | MG019517 |
| *A. gallagheri* |  | IBECN8040 |  | UAE | 25.459 | 56.184 | yes | MG019421 | MG019680 | MG019513 |
| *A. gallagheri* |  | IBECN7106 |  | UAE | 25.324 | 56.163 | yes | MG019460 | MG019700 | MG019493 |
| *A. gallagheri* |  |  |  | UAE | 25.324 | 56.163 |  | MG019458 | MG019697 | MG019507 |
| *A. gallagheri* |  |  |  | UAE | 25.324 | 56.163 |  | MG019459 | MG019701 | MG019494 |
| *A. gallagheri* |  |  |  | UAE | 25.324 | 56.162 |  | MG019456 | MG019647 | MG019509 |
| *A. gallagheri* |  | IBES7662 |  | UAE | 25.322 | 56.343 | yes | MG019463 | MG019698 | MG019551 |
| *A. gallagheri* |  |  |  | UAE | 25.182 | 56.229 |  | MG019444 | MG019646 | MG019508 |
| *A. gallagheri* |  |  |  | UAE | 25.182 | 56.229 |  | MG019439 | MG019648 | MG019505 |
| *A. gallagheri* |  |  |  | UAE | 25.182 | 56.229 |  | MG019420 | MG019674 | MG019506 |
| *A. gallagheri* |  | IBECN14 |  | UAE | 25.182 | 56.226 | yes | MG019443 | MG019650 | MG019497 |
| *A. gallagheri* |  | IBECN96 |  | UAE | 25.182 | 56.226 | yes | MG019454 | MG019649 | MG019499 |
| *A. gallagheri* |  | IBECN89 |  | UAE | 25.016 | 56.209 | yes | MG019455 | MG019651 | MG019528 |
| *A. gallagheri* |  | IBECN2598 |  | UAE | 25.016 | 56.208 | yes | MG019453 | MG019654 | MG019533 |
| *A. gallagheri* |  | IBECN2606 |  | UAE | 25.016 | 56.208 | yes | MG019457 | MG019662 | MG019532 |
| *A. gallagheri* |  | IBECN2613 |  | UAE | 25.016 | 56.208 | yes | MG019452 | MG019661 | MG019535 |
| *A. gallagheri* |  | IBECN2800 |  | UAE | 25.016 | 56.208 | yes | MG019450 | MG019652 | MG019529 |
| *A. gallagheri* |  | IBECN2803 |  | UAE | 25.016 | 56.208 | yes | MG019449 | MG019653 | MG019530 |
| *A. gallagheri* |  | IBECN2817 |  | UAE | 25.016 | 56.208 | yes | MG019448 | MG019660 | MG019531 |
| *A. gallagheri* |  | IBECN2790 |  | UAE | 25.015 | 56.210 | yes | MG019451 | MG019655 | MG019534 |
| *A. gallagheri* |  | TW1037 |  | UAE | 24.991 | 56.217 | yes | MG019442 | MG019659 | MG019561 |
| *A. gallagheri* |  | TW1038 |  | UAE | 24.991 | 56.217 | yes | MG019441 | MG019663 | MG019562 |
| *A. gallagheri* |  | TW1039 |  | UAE | 24.991 | 56.217 | yes | MG019440 | MG019664 | MG019563 |
| *A. gallagheri* |  | IBECN4024 |  | Oman | 24.621 | 56.339 | yes | MG019445 | MG019656 | MG019538 |
| *A. gallagheri* |  | IBECN3728 |  | Oman | 24.621 | 56.339 | yes | MG019447 | MG019657 | MG019539 |
| *A. gallagheri* |  | IBECN4011 |  | Oman | 24.621 | 56.340 | yes | MG019446 | MG019658 | MG019540 |
| *A. gallagheri* |  | IBECN118 |  | Oman | 24.513 | 56.463 | yes | MG019474 | MG019672 | MG019548 |
| *A. gallagheri* |  | IBECN195 |  | Oman | 24.513 | 56.463 | yes | MG019473 | MG019671 | MG019549 |
| *A. gallagheri* |  | IBECN3484 |  | Oman | 24.513 | 56.463 | yes | MG019471 | MG019667 | MG019544 |
| *A. gallagheri* |  | IBECN3524 |  | Oman | 24.513 | 56.463 | yes | MG019470 | MG019673 | MG019543 |
| *A. gallagheri* |  | IBECN3739 |  | Oman | 24.513 | 56.463 | yes | MG019467 | MG019665 | MG019541 |
| *A. gallagheri* |  | IBECN4167 |  | Oman | 24.513 | 56.463 | yes | MG019466 | MG019666 | MG019542 |
| *A. gallagheri* |  | IBECN4313 |  | Oman | 24.513 | 56.463 | yes | MG019465 | MG019670 | MG019547 |
| *A. gallagheri* |  | IBECN5817 |  | Oman | 24.513 | 56.463 | yes | MG019464 | MG019668 | MG019545 |
| *A. gallagheri* |  | IBECN217 |  | Oman | 24.513 | 56.464 | yes | MG019472 | MG019669 | MG019546 |
| *A. gallagheri* |  | IBECN2593 |  | Oman | 23.192 | 57.199 | yes | MG019477 | MG019702 | MG019537 |
| *A. gallagheri* |  | IBECN6034 |  | Oman | 23.173 | 57.432 | yes | MG019478 | MG019703 | MG019566 |
| *A. gardneri* |  |  |  | Oman |  |  | yes | KX550444 | KX550540 | KX550714 |
| *A. gardneri* |  | ONHM4221 |  | Oman |  |  | yes | KX550445 | KX550541 | KX550715 |
| *A. gardneri* |  | IBECN3042 |  | Oman |  |  | yes | KX550446 | KX550542 | KX550716 |
| *A. gardneri* |  |  |  | Oman |  |  | yes | KX550479 | KX550572 | KX550746 |
| *A. gardneri* |  | IBECN3681 |  | Oman |  |  | yes | KX550447 | KX550543 | KX550717 |
| *A. gardneri* |  |  |  | Oman |  |  | yes | KX550448 | KX550544 | KX550718 |
| *A. gardneri* |  | IBECN3901 |  | Oman |  |  | yes | KX550487 | KX550580 | KX550754 |
| *A. gardneri* |  | IBECN3903 |  | Oman |  |  | yes | KX550480 | KX550573 | KX550747 |
| *A. gardneri* |  | IBECN3904 |  | Oman |  |  | yes | KX550463 | KX550558 | KX550732 |
| *A. gardneri* |  | BMNH2008.100 |  | Oman |  |  | yes | KX550485 | KX550578 | KX550752 |
| *A. gardneri* |  | IBECN3907 |  | Oman |  |  | yes | KX550449 | KX550545 | KX550719 |
| *A. gardneri* |  | IBECN3910 |  | Oman |  |  | yes | KX550481 | KX550574 | KX550748 |
| *A. gardneri* |  | IBECN3914 |  | Oman |  |  | yes | KX550450 | KX550546 | KX550720 |
| *A. gardneri* |  | IBECN3955 |  | Oman |  |  | yes | KX550464 | KX550559 | KX550733 |
| *A. gardneri* |  | IBECN3965 |  | Oman |  |  | yes | KX550489 | KX550582 | KX550756 |
| *A. gardneri* |  | BMNH2008.999 |  | Oman |  |  | yes | KX550451 | KX550547 | KX550721 |
| *A. gardneri* |  | IBECN751 |  | Oman |  |  | yes | KX550452 | KX550548 | KX550722 |
| *A. gardneri* |  | IBECN757 |  | Oman |  |  | yes | KX550453 | KX550549 | KX550723 |
| *A. gardneri* |  |  |  | Oman |  |  | yes | KX550454 | KX550550 | KX550724 |
| *A. gardneri* |  | IBECN784 |  | Oman |  |  | yes | KX550482 | KX550575 | KX550749 |
| *A. gardneri* |  | IBECN797 |  | Oman |  |  | yes | KX550486 | KX550579 | KX550753 |
| *A. gardneri* |  | IBECN801 |  | Oman |  |  | yes | KX550455 | KX550551 | KX550725 |
| *A. gardneri* |  | IBECN8109 |  | Oman |  |  | yes | KX550469 | KX550562 | KX550736 |
| *A. gardneri* |  | IBECN817 |  | Oman |  |  | yes | KX550456 | KX550552 | KX550726 |
| *A. gardneri* |  | IBECN8370 |  | Oman |  |  | yes | KX550472 | KX550565 | KX550739 |
| *A. gardneri* |  | IBECN842 |  | Oman |  |  | yes | KX550488 | KX550581 | KX550755 |
| *A. gardneri* |  | IBECN844 |  | Oman |  |  | yes | KX550457 | KX550553 | *KX550727* |
| *A. gardneri* |  | IBECN848 |  | Oman |  |  | yes | KX550458 | KX550554 | KX550728 |
| *A. gardneri* |  | IBECN8673 |  | Oman |  |  | yes | KX550470 | KX550563 | KX550737 |
| *A. gardneri* |  | IBECN8700 |  | UAE |  |  | yes | KX550477 | KX550570 | KX550744 |
| *A. gardneri* |  | IBECN8701 |  | Oman |  |  | yes | KX550461 | KX550556 | KX550730 |
| *A. gardneri* |  | IBECN8715 |  | UAE |  |  | yes | KX550484 | KX550577 | KX550751 |
| *A. gardneri* |  | IBECN9008 |  | UAE |  |  | yes | KX550471 | KX550564 | KX550738 |
| *A. gardneri* |  |  |  | UAE |  |  | yes | KX550478 | KX550571 | KX550745 |
| *A. gardneri* |  | IBECN9024 |  | UAE |  |  | yes | KX550462 | KX550557 | KX550731 |
| *A. gardneri* |  | IBECN10426 |  | Oman |  |  | yes | KX550473 | KX550566 | KX550740 |
| *A. gardneri* |  | IBECN10427 |  | Oman |  |  | yes | KX550466 | KX550560 | KX550734 |
| *A. gardneri* |  |  |  | Oman |  |  | yes | KX550474 | KX550567 | KX550741 |
| *A. gardneri* |  | IBECN10428 |  | UAE |  |  | yes | KX550476 | KX550569 | KX550743 |
| *A. gardneri* |  |  |  | Oman |  |  | yes | KX550490 | KX550583 | KX550757 |
| *A. gardneri* |  |  |  | Oman |  |  | yes | KX550483 | KX550576 | KX550750 |
| *A. gardneri* |  |  |  | Oman |  |  | yes | KX550459 | KX550555 | KX550729 |
| *A. margaritae* |  | CAS250891 |  | Oman |  |  |  | KX550522 | KX550615 | KX550788 |
| *A. margaritae* |  | CAS250891 |  | Oman |  |  |  | KX550524 | KX550617 | KX550790 |
| *A. margaritae* |  | IBECN2997 |  | Oman |  |  | yes | KX550496 | KX550589 | KX550763 |
| *A. margaritae* |  | IBECN3592 |  | Oman |  |  |  | KX550498 | KX550591 | KX550765 |
| *A. margaritae* |  | ONHM4222 |  | UAE |  |  | yes | KX550505 | KX550598 | KX550772 |
| *A. margaritae* |  | BMNH2008.989 |  | UAE |  |  | yes | KX550517 | KX550610 | KX550783 |
| *A. margaritae* |  | BMNH2008.988 |  | UAE |  |  | yes | KX550523 | KX550616 | KX550789 |
| *A. margaritae* |  | IBECN7126 |  | Oman |  |  |  | KX550502 | KX550595 | KX550769 |
| *A. margaritae* |  |  |  | UAE |  |  |  | KX550506 | KX550599 | KX550773 |
| *A. margaritae* |  |  |  | UAE |  |  |  | KX550507 | KX550600 | KX550774 |
| *A. margaritae* |  |  |  | UAE |  |  |  | KX550508 | KX550601 | KX550775 |
| *A. margaritae* |  | IBECN8191 |  | Oman |  |  | yes | KX550499 | KX550592 | KX550766 |
| *A. margaritae* |  | IBECN8195 |  | Oman |  |  |  | KX550500 | KX550593 | KX550767 |
| *A. margaritae* |  |  |  | UAE |  |  |  | KX550509 | KX550602 | KX550776 |
| *A. margaritae* |  |  |  | UAE |  |  |  | KX550503 | KX550596 | KX550770 |
| *A. margaritae* |  |  |  | UAE |  |  |  | KX550510 | KX550603 | KX550777 |
| *A. margaritae* |  | IBECN8631 |  | Oman |  |  |  | KX550501 | KX550594 | KX550768 |
| *A. margaritae* |  |  |  | UAE |  |  |  | KX550511 | KX550604 | KX550778 |
| *A. margaritae* |  |  |  | UAE |  |  |  | KX550512 | KX550605 | KX550779 |
| *A. margaritae* |  | IBECN8708 |  | UAE |  |  | yes | KX550518 | KX550611 | KX550784 |
| *A. margaritae* |  | IBECN9012 |  | UAE |  |  | yes | KX550519 | KX550612 | KX550785 |
| *A. margaritae* |  | IBECN9020 |  | UAE |  |  | yes | KX550520 | KX550613 | KX550786 |
| *A. margaritae* |  | IBECN9023 |  | UAE |  |  | yes | KX550521 | KX550614 | KX550787 |
| *A. margaritae* |  | IBECN10419 |  | UAE |  |  | yes | KX550513 | KX550606 | KX550780 |
| *A. margaritae* |  | IBECN10420 |  | UAE |  |  | yes | KX550504 | KX550597 | KX550771 |
| *A. margaritae* |  | IBECN10421 |  | UAE |  |  | yes | KX550514 | KX550607 | KX550781 |
| *A. margaritae* |  |  |  | UAE |  |  | yes | KX550516 | KX550609 | KX550782 |
| *A. montanus* |  |  |  | Oman |  |  |  | MH752841 |  | MH752942 |
| *A. montanus* |  |  |  | Oman |  |  |  | KX550530 | KX550537 | KX550711 |
| *A. montanus* |  |  |  | Oman |  |  |  | MH752844 |  | MH752945 |
| *A. montanus* |  |  |  | Oman |  |  |  | MH752848 |  | MH752946 |
| *A. montanus* |  |  |  | Oman |  |  |  | MH752861 |  | MH752947 |
| *A. montanus* |  |  |  | Oman |  |  |  | MH752864 |  | MH752969 |
| *A. montanus* |  |  |  | Oman |  |  |  | MH752849 |  | MH752948 |
| *A. montanus* |  |  |  | Oman |  |  |  | MH752873 |  | MH752949 |
| *A. montanus* |  |  |  | Oman |  |  |  | MH752865 |  | MH752970 |
| *A. montanus* |  |  |  | Oman |  |  |  | MH782871 |  | MH752975 |
| *A. montanus* |  |  |  | Oman |  |  |  | MH752874 |  | MH752961 |
| *A. montanus* |  |  |  | Oman |  |  |  | MH752845 |  | MH752950 |
| *A. montanus* |  |  |  | Oman |  |  |  | MH752872 |  | MH752971 |
| *A. montanus* |  |  |  | Oman |  |  |  | MH752866 |  | MH752972 |
| *A. montanus* |  |  |  | Oman |  |  |  | MH752867 |  | MH752962 |
| *A. montanus* |  |  |  | Oman |  |  |  | MH752868 |  | MH752951 |
| *A. montanus* |  |  |  | Oman |  |  |  | MH752862 |  | MH752973 |
| *A. montanus* |  |  |  | Oman |  |  |  | MH752863 |  | MH752963 |
| *A. montanus* |  |  |  | Oman |  |  |  | MH752869 |  | MH752974 |
| *A. montanus* |  |  |  | Oman |  |  |  | MH752870 |  | MH752964 |
| *A. montanus* |  |  |  | Oman |  |  |  | MH752850 |  | MH752952 |
| *A. montanus* |  |  |  | Oman |  |  |  | MH752854 |  | MH752965 |
| *A. montanus* |  |  |  | Oman |  |  |  | MH752857 |  | MH752953 |
| *A. montanus* |  |  |  | Oman |  |  |  | MH752860 |  | MH752966 |
| *A. montanus* |  |  |  | Oman |  |  |  | MH752851 |  | MH752954 |
| *A. montanus* |  |  |  | Oman |  |  |  | MH752852 |  | MH752955 |
| *A. montanus* |  |  |  | Oman |  |  |  | MH752858 |  | MH752956 |
| *A. montanus* |  |  |  | Oman |  |  |  | MH752855 |  | MH752957 |
| *A. montanus* |  |  |  | Oman |  |  |  | MH752853 |  | MH752967 |
| *A. montanus* |  |  |  | Oman |  |  |  | MH752856 |  | MH752958 |
| *A. montanus* |  |  |  | Oman |  |  |  | MH752859 |  | MH752968 |
| *A. montanus* |  |  |  | Oman |  |  |  | MH752846 |  | MH752959 |
| *A. montanus* |  |  |  | Oman |  |  |  | MH752847 |  | MH752960 |
| *A. griseonotus* |  | MVZ234326 |  | Iran | 33.2595 | 47.80417 |  | KX550528 | KX550535 | KX550709 |
| *A. elisae* |  | MVZ234315 |  | Iran | 32.84867 | 48.26417 |  | KX550527 |  | KX550708 |
| *A. nasrullahi* |  | MVZ234330 |  | Iran | 32.84867 | 48.26417 |  | KX550529 | KX550536 | KX550710 |
| *A. platyrhynchus* |  | IBES1751 |  | Oman |  |  |  | KX550525 | KX550533 | KX550706 |
| **Outgroup species** |  |  |  |  |  |  |  |  |  |  |
| *Ptyodactylus hasselquistii* |  | E603625 |  |  |  |  |  | KX981451 | KP868045 | KP868093 |
| *Ptyodactylus ruusaljibalicus* |  | CN3951 |  |  |  |  |  | MF084456 | MF084691 | MF084517 |

* Specimen collected in this study

** Sequenced specimens

T Specimens collected from the type locality
