# Supplementary material for: Integrating phylogenetic, phylogeographic, and morphometric analyses to reveal cryptic lineages within the genus Asaccus (Reptilia: Squamata: Phyllodactylidae) in Iran
Source: BMC Zool. 2024 Jun 26;9:12. doi: 10.1186/s40850-024-00203-1 (PMC11202258; doi:10.1186/s40850-024-00203-1)
Supplement: Supplementary file 2 — Supplementary Material 2 [file 40850_2024_203_MOESM2_ESM.docx]

**Table S2.** Uncorrected interspecific genetic distances between all *Asaccus* species. *p*-distances for 12S gene (lower-left) and SE estimates (upper-rights) 1:*A*. *arnoldi*; 2: *A*. *caudivolvulus*; 3: *A*. *gallagheri* 4: *A*. *gardneri*; 5: *A*. *griseonotus*; 6: *A*. *margaritae*; 7: *A*. *platyrhynchus*; 8: *A*. *nasrullahi*; 9: *A*. *montanus*; 10: *A*. *elisae*; 11: *A*. *iranicus*; 12: *A*. *kurdistanensis*; 13: *A*. *granularis*; 14: *A*. *zagrosicus*; 15: **Population 7**; 16: *A*. *tangestanensis*; 17: **Population 9**; 18: **Population 10**; 19: **Population 4**; 20: **Population 8**; 21: **Population 6**; 22: *A*. *kermanshahensis*; 23: **Population 3**; 24: **Population 1**; 25: **Population 5**; 26: **Population 2**; 27: *A*. *andersoni*

|  | 1 | 2 | 3 | 4 | 5 | 6 | 7 | 8 | 9 | 10 | 11 | 12 | 13 | 14 | 15 | 16 | 17 | 18 | 19 | 20 | 21 | 22 | 23 | 24 | 25 | 26 | 27 |
| --- | --- | --- | --- | --- | --- | --- | --- | --- | --- | --- | --- | --- | --- | --- | --- | --- | --- | --- | --- | --- | --- | --- | --- | --- | --- | --- | --- |
| 1 |  | **0.018** | **0.014** | **0.017** | **0.016** | **0.017** | **0.015** | **0.016** | **0.017** | **0.019** | **0.018** | **0.017** | **0.018** | **0.018** | **0.018** | **0.018** | **0.018** | **0.016** | **0.018** | **0.017** | **0.019** | **0.015** | **0.018** | **0.017** | **0.018** | **0.017** | **0.018** |
| 2 | **0.187** |  | **0.018** | **0.011** | **0.017** | **0.017** | **0.020** | **0.019** | **0.020** | **0.021** | **0.019** | **0.019** | **0.020** | **0.020** | **0.020** | **0.019** | **0.022** | **0.019** | **0.020** | **0.019** | **0.019** | **0.018** | **0.020** | **0.019** | **0.020** | **0.019** | **0.019** |
| 3 | **0.115** | **0.183** |  | **0.019** | **0.017** | **0.017** | **0.014** | **0.018** | **0.019** | **0.020** | **0.018** | **0.018** | **0.018** | **0.020** | **0.018** | **0.019** | **0.020** | **0.017** | **0.019** | **0.018** | **0.019** | **0.016** | **0.019** | **0.019** | **0.019** | **0.018** | **0.017** |
| 4 | **0.169** | **0.069** | **0.183** |  | **0.016** | **0.017** | **0.019** | **0.017** | **0.019** | **0.020** | **0.020** | **0.018** | **0.019** | **0.020** | **0.020** | **0.019** | **0.020** | **0.018** | **0.020** | **0.018** | **0.019** | **0.017** | **0.020** | **0.018** | **0.020** | **0.017** | **0.019** |
| 5 | **0.172** | **0.170** | **0.181** | **0.158** |  | **0.017** | **0.019** | **0.005** | **0.018** | **0.018** | **0.016** | **0.016** | **0.016** | **0.017** | **0.017** | **0.016** | **0.018** | **0.015** | **0.017** | **0.013** | **0.017** | **0.014** | **0.017** | **0.016** | **0.017** | **0.009** | **0.014** |
| 6 | **0.153** | **0.167** | **0.161** | **0.152** | **0.185** |  | **0.017** | **0.018** | **0.020** | **0.021** | **0.020** | **0.018** | **0.021** | **0.020** | **0.020** | **0.020** | **0.021** | **0.018** | **0.020** | **0.019** | **0.019** | **0.017** | **0.020** | **0.019** | **0.019** | **0.018** | **0.018** |
| 7 | **0.131** | **0.189** | **0.112** | **0.185** | **0.197** | **0.136** |  | **0.019** | **0.020** | **0.021** | **0.019** | **0.019** | **0.020** | **0.021** | **0.020** | **0.019** | **0.021** | **0.018** | **0.021** | **0.020** | **0.019** | **0.018** | **0.019** | **0.019** | **0.020** | **0.019** | **0.020** |
| 8 | **0.157** | **0.162** | **0.171** | **0.147** | **0.044** | **0.175** | **0.186** |  | **0.020** | **0.020** | **0.018** | **0.016** | **0.019** | **0.018** | **0.018** | **0.017** | **0.019** | **0.016** | **0.019** | **0.015** | **0.019** | **0.015** | **0.018** | **0.017** | **0.019** | **0.010** | **0.015** |
| 9 | **0.181** | **0.232** | **0.217** | **0.225** | **0.215** | **0.227** | **0.198** | **0.214** |  | **0.020** | **0.019** | **0.019** | **0.019** | **0.019** | **0.020** | **0.018** | **0.020** | **0.019** | **0.020** | **0.019** | **0.020** | **0.019** | **0.020** | **0.019** | **0.020** | **0.020** | **0.019** |
| 10 | **0.207** | **0.241** | **0.223** | **0.218** | **0.205** | **0.225** | **0.221** | **0.213** | **0.234** |  | **0.018** | **0.019** | **0.016** | **0.002** | **0.013** | **0.018** | **0.018** | **0.019** | **0.017** | **0.017** | **0.020** | **0.018** | **0.018** | **0.019** | **0.020** | **0.020** | **0.021** |
| 11 | **0.173** | **0.199** | **0.184** | **0.195** | **0.177** | **0.187** | **0.198** | **0.173** | **0.190** | **0.167** |  | **0.018** | **0.016** | **0.018** | **0.018** | **0.002** | **0.015** | **0.018** | **0.016** | **0.018** | **0.017** | **0.017** | **0.016** | **0.014** | **0.016** | **0.018** | **0.017** |
| 12 | **0.169** | **0.192** | **0.183** | **0.169** | **0.162** | **0.174** | **0.187** | **0.152** | **0.222** | **0.192** | **0.159** |  | **0.019** | **0.019** | **0.019** | **0.018** | **0.018** | **0.009** | **0.017** | **0.017** | **0.020** | **0.016** | **0.018** | **0.016** | **0.018** | **0.016** | **0.017** |
| 13 | **0.179** | **0.176** | **0.179** | **0.170** | **0.144** | **0.191** | **0.196** | **0.163** | **0.199** | **0.097** | **0.117** | **0.167** |  | **0.015** | **0.013** | **0.016** | **0.016** | **0.018** | **0.016** | **0.017** | **0.018** | **0.018** | **0.016** | **0.017** | **0.018** | **0.019** | **0.019** |
| 14 | **0.195** | **0.232** | **0.219** | **0.211** | **0.201** | **0.223** | **0.215** | **0.207** | **0.222** | **0.002** | **0.159** | **0.182** | **0.097** |  | **0.013** | **0.017** | **0.018** | **0.019** | **0.016** | **0.017** | **0.019** | **0.018** | **0.017** | **0.017** | **0.018** | **0.018** | **0.019** |
| 15 | **0.165** | **0.187** | **0.165** | **0.178** | **0.156** | **0.189** | **0.182** | **0.159** | **0.209** | **0.072** | **0.136** | **0.162** | **0.071** | **0.077** |  | **0.018** | **0.018** | **0.018** | **0.017** | **0.017** | **0.019** | **0.017** | **0.018** | **0.017** | **0.018** | **0.018** | **0.019** |
| 16 | **0.180** | **0.205** | **0.194** | **0.201** | **0.186** | **0.195** | **0.208** | **0.180** | **0.197** | **0.181** | **0.007** | **0.168** | **0.117** | **0.170** | **0.137** |  | **0.015** | **0.017** | **0.016** | **0.018** | **0.017** | **0.017** | **0.016** | **0.014** | **0.016** | **0.018** | **0.017** |
| 17 | **0.186** | **0.218** | **0.200** | **0.209** | **0.179** | **0.214** | **0.201** | **0.179** | **0.199** | **0.147** | **0.112** | **0.158** | **0.119** | **0.145** | **0.137** | **0.113** |  | **0.018** | **0.016** | **0.018** | **0.019** | **0.018** | **0.017** | **0.016** | **0.017** | **0.019** | **0.019** |
| 18 | **0.145** | **0.152** | **0.155** | **0.135** | **0.129** | **0.153** | **0.164** | **0.118** | **0.198** | **0.175** | **0.145** | **0.049** | **0.158** | **0.174** | **0.166** | **0.148** | **0.156** |  | **0.018** | **0.016** | **0.019** | **0.015** | **0.018** | **0.015** | **0.018** | **0.016** | **0.016** |
| 19 | **0.214** | **0.238** | **0.214** | **0.233** | **0.207** | **0.219** | **0.234** | **0.207** | **0.235** | **0.166** | **0.136** | **0.160** | **0.111** | **0.155** | **0.133** | **0.146** | **0.108** | **0.160** |  | **0.018** | **0.019** | **0.018** | **0.016** | **0.017** | **0.017** | **0.018** | **0.018** |
| 20 | **0.157** | **0.146** | **0.165** | **0.134** | **0.091** | **0.157** | **0.164** | **0.084** | **0.193** | **0.117** | **0.157** | **0.127** | **0.127** | **0.116** | **0.134** | **0.159** | **0.150** | **0.121** | **0.151** |  | **0.018** | **0.016** | **0.018** | **0.017** | **0.019** | **0.016** | **0.018** |
| 21 | **0.178** | **0.154** | **0.178** | **0.170** | **0.164** | **0.158** | **0.172** | **0.165** | **0.196** | **0.182** | **0.141** | **0.171** | **0.152** | **0.178** | **0.159** | **0.141** | **0.169** | **0.164** | **0.190** | **0.162** |  | **0.018** | **0.019** | **0.018** | **0.019** | **0.019** | **0.019** |
| 22 | **0.138** | **0.163** | **0.149** | **0.142** | **0.137** | **0.150** | **0.154** | **0.127** | **0.207** | **0.189** | **0.157** | **0.126** | **0.166** | **0.187** | **0.164** | **0.162** | **0.174** | **0.102** | **0.200** | **0.119** | **0.157** |  | **0.017** | **0.017** | **0.019** | **0.015** | **0.016** |
| 23 | **0.198** | **0.233** | **0.198** | **0.226** | **0.212** | **0.212** | **0.204** | **0.213** | **0.244** | **0.187** | **0.148** | **0.197** | **0.135** | **0.184** | **0.158** | **0.151** | **0.145** | **0.173** | **0.163** | **0.163** | **0.175** | **0.190** |  | **0.015** | **0.017** | **0.018** | **0.018** |
| 24 | **0.184** | **0.207** | **0.201** | **0.198** | **0.196** | **0.214** | **0.208** | **0.192** | **0.220** | **0.186** | **0.110** | **0.172** | **0.132** | **0.182** | **0.139** | **0.116** | **0.120** | **0.142** | **0.162** | **0.126** | **0.167** | **0.192** | **0.146** |  | **0.015** | **0.018** | **0.017** |
| 25 | **0.181** | **0.218** | **0.176** | **0.206** | **0.206** | **0.206** | **0.197** | **0.201** | **0.239** | **0.198** | **0.145** | **0.185** | **0.143** | **0.202** | **0.149** | **0.154** | **0.142** | **0.169** | **0.178** | **0.167** | **0.181** | **0.207** | **0.183** | **0.132** |  | **0.019** | **0.019** |
| 26 | **0.169** | **0.166** | **0.181** | **0.140** | **0.081** | **0.165** | **0.175** | **0.057** | **0.228** | **0.201** | **0.183** | **0.145** | **0.166** | **0.201** | **0.151** | **0.192** | **0.188** | **0.118** | **0.221** | **0.102** | **0.164** | **0.132** | **0.223** | **0.191** | **0.199** |  | **0.015** |
| 27 | **0.167** | **0.166** | **0.165** | **0.189** | **0.129** | **0.180** | **0.185** | **0.118** | **0.209** | **0.235** | **0.162** | **0.160** | **0.158** | **0.232** | **0.170** | **0.169** | **0.160** | **0.122** | **0.196** | **0.133** | **0.153** | **0.134** | **0.195** | **0.194** | **0.205** | **0.122** |  |
