# Supplementary material for: Integrating phylogenetic, phylogeographic, and morphometric analyses to reveal cryptic lineages within the genus Asaccus (Reptilia: Squamata: Phyllodactylidae) in Iran
Source: BMC Zool. 2024 Jun 26;9:12. doi: 10.1186/s40850-024-00203-1 (PMC11202258; doi:10.1186/s40850-024-00203-1)
Supplement: Supplementary file 3 — Supplementary Material 3 [file 40850_2024_203_MOESM3_ESM.docx]

| Codons | BEAST | IQtree | MrBayes | | |
| --- | --- | --- | --- | --- | --- |
|  |  |  | Substitution model | nst | rates |
| Cyt *b*-codon1 | GTR+I+G+X | GTR+I+G | GTR+I+G | 6 | invgamma |
| Cyt *b*-codon2 | GTR+G+X | GTR+G | GTR+G | 6 | gamma |
| Cyt *b*-codon3 | TRN+I+G+X | HKY+G | HKY+G | 2 | gamma |
| *c-mos*-codon1 | K80+I | K80+I | K80+I | 2 | propinv |
| *c-mos*-codon2 | K80+I | K80+I | K80+I | 2 | propinv |
| *c-mos*-codon3 | K80+G | K80+G | K80+G | 2 | gamma |
| 12S rRNA | GTR+I+G+X | GTR+I+G | GTR+I+G | 6 | invgamma |

**Table S3.** Best-fit partitioning schemes and substitution models for molecular evolution based on BEAST, IQtree, and MrBayes.
