# Supplementary material for: Integrating phylogenetic, phylogeographic, and morphometric analyses to reveal cryptic lineages within the genus Asaccus (Reptilia: Squamata: Phyllodactylidae) in Iran
Source: BMC Zool. 2024 Jun 26;9:12. doi: 10.1186/s40850-024-00203-1 (PMC11202258; doi:10.1186/s40850-024-00203-1)
Supplement: Supplementary file 4 — Supplementary Material 4 [file 40850_2024_203_MOESM4_ESM.docx]

**Table S4.** Sex, metric and meristic variables of *Asaccus* species used in the study; **SVL**: snouth-vent length; **TrL**: trunk length; **HL**: head length; **HW**: head width; **HH**: head height; **SL**: snout length; **SW**: snout width; **ED**: eye diameter; **Lun**: ulna length; **LHu**: humerus length; **LTb**: tibia length; **LFe**: femur length; **Trow**: rows of dorsal tubercles; **LT4**: number of expanded lamellae rows under the 4th toe; **ULS**: upper labials; **LLS**: lower labials; **PMS**: postmentals.

| **Species name** | **Voucher code** | **Sex** | **SVL** | **TrL** | **HL** | **HW** | **HH** | **SL** | **SW** | **ED** | **LUn** | **LHu** | **LTb** | **LFe** | **Trow** | **LT4** | **ULS** | **LLS** | **PMS** |
| --- | --- | --- | --- | --- | --- | --- | --- | --- | --- | --- | --- | --- | --- | --- | --- | --- | --- | --- | --- |
| *A. kurdistanensis* | NEZMUT1495 | M | 60.38 | 29.72 | 15.82 | 10.46 | 5.28 | 7.80 | 9.58 | 3.80 | 8.38 | 8.90 | 11.84 | 13.28 | 13 | 8 | 11 | 8 | 3 |
| *A. kurdistanensis* | NEZMUT1496 | M | 61.74 | 27.46 | 16.10 | 11.08 | 5.62 | 7.76 | 9.60 | 3.54 | 9.82 | 8.30 | 10.90 | 11.62 | 14 | 9 | 12 | 8 | 3 |
| *A. kurdistanensis* | NEZMUT1497 | F | 58.30 | 28.10 | 15.26 | 10.40 | 4.96 | 6.94 | 8.82 | 3.56 | 8.42 | 9.02 | 10.84 | 11.76 | 14 | 9 | 13 | 8 | 2 |
| *A. kurdistanensis* | NEZMUT1498 | M | 59.48 | 27.80 | 14.92 | 10.56 | 5.28 | 6.80 | 8.92 | 3.32 | 9.02 | 7.80 | 11.42 | 13.54 | 14 | 9 | 12 | 8 | 2 |
| *A. kurdistanensis* | NEZMUT1499 | F | 53.72 | 24.84 | 13.90 | 9.12 | 4.80 | 6.96 | 8.72 | 3.60 | 8.24 | 7.80 | 9.72 | 12.00 | 13 | 9 | 12 | 9 | 2 |
| *A. kurdistanensis* | NEZMUT1339 | M | 47.60 | 26.90 | 13.00 | 9.00 | 4.32 | 5.64 | 6.54 | 3.62 | 7.76 | 7.98 | 9.00 | 12.01 | 13 | 9 | 11 | 8 | 3 |
| *A. kurdistanensis* | NEZMUT1340 | J | 27.06 | 13.82 | 8.34 | 5.82 | 2.90 | 4.42 | 3.72 | 2.26 | 4.12 | 3.40 | 5.72 | 6.52 | 13 | 8 | 11 | 8 | 3 |
| *A. kurdistanensis* | NEZMUT1341 | F | 51.34 | 26.76 | 13.54 | 9.58 | 5.98 | 6.78 | 7.76 | 3.62 | 8.54 | 7.56 | 10.00 | 8.54 | 14 | 8 | 12 | 10 | 2 |
| *A. kurdistanensis* | NEZMUT1342 | F | 53.84 | 29.32 | 14.38 | 9.74 | 5.20 | 6.38 | 7.16 | 3.84 | 8.04 | 7.98 | 10.20 | 11.62 | 12 | 9 | 12 | 8 | 2 |
| *A. kurdistanensis* | NEZMUT1501 | M | 49.20 | 20.88 | 12.76 | 9.80 | 4.88 | 6.26 | 8.14 | 3.58 | 7.94 | 7.32 | 9.10 | 11.16 | 14 | 9 | 11 | 8 | 4 |
| *A. kurdistanensis* | NEZMUT1502 | F | 54.62 | 26.20 | 14.22 | 9.88 | 5.18 | 6.86 | 8.04 | 3.90 | 8.54 | 7.50 | 11.00 | 12.26 | 15 | 9 | 11 | 8 | 3 |
| *A. kurdistanensis* | NEZMUT1503 | M | 59.38 | 27.18 | 15.82 | 11.26 | 5.90 | 7.60 | 9.78 | 3.90 | 9.36 | 9.80 | 11.72 | 13.74 | 13 | 9 | 12 | 9 | 3 |
| *A. kurdistanensis* | NEZMUT1504 | F | 50.20 | 26.02 | 14.32 | 9.88 | 4.98 | 6.38 | 9.02 | 3.74 | 8.16 | 8.48 | 10.64 | 12.40 | 13 | 10 | 13 | 8 | 2 |
| *A. kurdistanensis* | NEZMUT1505 | M | 60.78 | 26.66 | 16.18 | 11.03 | 6.30 | 7.10 | 9.72 | 3.70 | 8.50 | 8.74 | 11.64 | 13.52 | 14 | 9 | 11 | 8 | 3 |
| *A. kurdistanensis* | NEZMUT1506 | F | 52.82 | 24.38 | 14.54 | 9.42 | 5.24 | 6.48 | 8.58 | 3.64 | 8.64 | 7.48 | 10.42 | 11.80 | 14 | 9 | 12 | 9 | 3 |
| *A. kurdistanensis* | NEZMUT1343 | M | 52.36 | 31.38 | 13.54 | 9.92 | 4.02 | 6.68 | 7.16 | 3.44 | 9.72 | 7.96 | 10.34 | 11.58 | 13 | 8 | 12 | 8 | 2 |
| *A. kurdistanensis* | NEZMUT1344 | F | 55.54 | 30.90 | 13.90 | 9.42 | 4.82 | 6.22 | 8.84 | 4.12 | 9.38 | 8.64 | 10.52 | 11.98 | 13 | 10 | 11 | 8 | 2 |
| *A. kurdistanensis* | NEZMUT1345 | F | 54.70 | 30.54 | 13.54 | 10.00 | 5.38 | 6.44 | 7.82 | 3.34 | 9.90 | 9.82 | 10.14 | 12.12 | 13 | 8 | 10 | 8 | 2 |
| *A. kurdistanensis* | NEZMUT1346 | F | 54.84 | 33.12 | 15.16 | 9.82 | 5.00 | 6.62 | 7.96 | 3.34 | 8.80 | 9.52 | 10.64 | 12.20 | 13 | 10 | 11 | 8 | 2 |
| *A. kurdistanensis* | NEZMUT1347 | F | 47.48 | 24.82 | 12.32 | 8.44 | 5.02 | 5.00 | 7.84 | 3.24 | 7.92 | 8.90 | 9.84 | 11.32 | 12 | 9 | 10 | 9 | 2 |
| **Population 1** | NEZMUT1352 | F | 45.28 | 20.24 | 11.32 | 6.92 | 4.02 | 6.14 | 7.02 | 3.18 | 8.34 | 9.98 | 10.34 | 10.11 | 12 | 9 | 12 | 10 | 3 |
| **Population 1** | NEZMUT1353 | F | 49.28 | 25.76 | 12.99 | 7.99 | 4.99 | 6.78 | 7.32 | 3.20 | 7.46 | 7.56 | 10.42 | 11.08 | 13 | 9 | 14 | 10 | 3 |
| **Population 1** | NEZMUT1354 | F | 47.18 | 21.80 | 12.06 | 8.10 | 4.78 | 6.08 | 7.14 | 3.14 | 8.38 | 9.84 | 10.32 | 10.40 | 12 | 9 | 13 | 10 | 3 |
| **Population 1** | NEZMUT1426 | F | 49.04 | 24.60 | 12.99 | 8.62 | 5.12 | 5.80 | 7.80 | 3.10 | 8.86 | 8.71 | 9.72 | 11.10 | 13 | 9 | 13 | 8 | 3 |
| **Population 1** | NEZMUT1427 | F | 51.42 | 23.90 | 12.38 | 8.46 | 4.30 | 5.40 | 7.26 | 3.34 | 8.92 | 8.68 | 9.26 | 10.58 | 12 | 8 | 13 | 9 | 3 |
| **Population 1** | NEZMUT1428 | M | 52.4 | 23.00 | 13.38 | 9.30 | 5.40 | 6.48 | 8.01 | 3.38 | 9.20 | 8.30 | 10.51 | 12.71 | 13 | 9 | 11 | 8 | 3 |
| *A. iranicus* | NEZMUT1421 | J | 35.78 | 16.80 | 10.74 | 5.98 | 4.02 | 5.06 | 5.18 | 3.28 | 7.20 | 6.34 | 9.46 | 10.44 | 11 | 10 | 11 | 9 | 2 |
| *A. iranicus* | NEZMUT1422 | J | 37.02 | 15.48 | 10.28 | 6.20 | 3.48 | 4.70 | 5.54 | 2.78 | 7.46 | 6.18 | 8.50 | 8.84 | 12 | 10 | 13 | 10 | 2 |
| *A. iranicus* | NEZMUT1423 | J | 40.54 | 17.14 | 10.38 | 5.66 | 3.64 | 4.60 | 5.40 | 3.08 | 7.50 | 6.62 | 8.54 | 9.41 | 12 | 10 | 11 | 9 | 3 |
| *A. iranicus* | NEZMUT1424 | F | 54.12 | 24.18 | 13.08 | 8.40 | 5.80 | 6.48 | 7.96 | 3.72 | 9.66 | 7.36 | 11.38 | 12.08 | 14 | 10 | 11 | 9 | 3 |
| *A. iranicus* | NEZMUT1359 | J | 35.40 | 14.10 | 10.40 | 6.82 | 3.18 | 4.71 | 5.62 | 2.91 | 6.48 | 6.68 | 7.89 | 8.98 | 14 | 10 | 11 | 10 | 2 |
| *A. iranicus* | NEZMUT1360 | SA | 43.86 | 19.78 | 11.68 | 6.92 | 4.32 | 6.08 | 6.64 | 3.10 | 8.50 | 7.60 | 10.24 | 10.82 | 14 | 9 | 13 | 10 | 2 |
| *A. iranicus* | NEZMUT1361 | F | 47.66 | 20.44 | 12.08 | 7.96 | 5.08 | 7.60 | 7.72 | 3.80 | 9.24 | 10.48 | 11.36 | 12.94 | 13 | 9 | 13 | 10 | 2 |
| *A. iranicus* | NEZMUT1362 | M | 54.98 | 22.72 | 14.42 | 9.56 | 6.48 | 7.00 | 8.38 | 4.32 | 10.20 | 9.70 | 12.70 | 13.30 | 12 | 9 | 13 | 10 | 2 |
| *A. iranicus* | NEZMUT1363 | M | 54.82 | 25.18 | 14.14 | 9.54 | 5.98 | 7.26 | 7.96 | 4.04 | 9.98 | 10.28 | 12.36 | 13.50 | 13 | 11 | 13 | 9 | 2 |
| *A. iranicus* | NEZMUT1364 | M | 52.42 | 22.16 | 13.43 | 9.20 | 5.60 | 6.58 | 7.72 | 3.70 | 10.30 | 8.91 | 12.16 | 13.18 | 13 | 10 | 12 | 10 | 2 |
| *A. iranicus* | NEZMUT1365 | M | 50.30 | 21.14 | 13.58 | 8.82 | 5.66 | 6.28 | 8.02 | 3.64 | 9.34 | 10.11 | 11.60 | 12.84 | 13 | 10 | 13 | 9 | 2 |
| *A. iranicus* | NEZMUT1366 | M | 50.84 | 22.90 | 13.48 | 9.06 | 5.62 | 6.08 | 7.84 | 3.42 | 8.78 | 10.34 | 10.82 | 12.46 | 13 | 9 | 13 | 9 | 2 |
| *A. iranicus* | NEZMUT1367 | J | 29.98 | 10.70 | 8.42 | 5.36 | 3.44 | 3.80 | 4.42 | 2.30 | 4.72 | 5.02 | 6.12 | 6.68 | 12 | 10 | 13 | 10 | 3 |
| *A. iranicus* | NEZMUT1368 | J | 32.22 | 12.84 | 9.12 | 6.38 | 3.68 | 4.32 | 4.82 | 2.54 | 5.88 | 6.08 | 6.99 | 7.70 | 13 | 10 | 14 | 8 | 2 |
| *A. iranicus* | NEZMUT1369 | SA | 41.40 | 18.12 | 11.34 | 6.58 | 3.99 | 5.46 | 6.30 | 2.90 | 7.98 | 6.50 | 9.39 | 9.16 | 14 | 9 | 12 | 10 | 3 |
| *A. iranicus* | NEZMUT1370 | F | 51.66 | 22.16 | 13.44 | 8.80 | 5.18 | 6.80 | 8.11 | 4.10 | 9.99 | 7.42 | 11.38 | 11.92 | 15 | 11 | 12 | 9 | 3 |
| *A. iranicus* | NEZMUT1371 | F | 51.64 | 20.68 | 13.28 | 9.01 | 5.98 | 6.50 | 8.58 | 4.12 | 9.72 | 10.14 | 10.20 | 11.88 | 15 | 10 | 12 | 9 | 3 |
| *A. iranicus* | NEZMUT1372 | M | 43.99 | 18.54 | 11.99 | 7.99 | 4.22 | 5.42 | 6.14 | 3.14 | 7.98 | 8.32 | 9.98 | 9.96 | 15 | 9 | 13 | 10 | 2 |
| *A. iranicus* | NEZMUT1373 | F | 49.12 | 21.72 | 12.96 | 8.38 | 5.52 | 6.18 | 7.50 | 3.44 | 9.44 | 9.16 | 11.58 | 10.98 | 12 | 11 | 13 | 10 | 2 |
| *A. iranicus* | NEZMUT1374 | F | 48.64 | 24.14 | 13.28 | 8.14 | 5.48 | 6.58 | 7.56 | 3.30 | 9.16 | 9.92 | 11.16 | 11.66 | 12 | 10 | 13 | 10 | 2 |
| *A. iranicus* | NEZMUT1375 | F | 53.50 | 27.36 | 14.82 | 9.66 | 5.48 | 7.44 | 8.78 | 3.70 | 9.82 | 8.40 | 12.14 | 11.98 | 13 | 10 | 13 | 10 | 2 |
| *A. iranicus* | NEZMUT1376 | F | 51.40 | 26.9 | 13.44 | 8.72 | 5.6 | 6.34 | 7.18 | 3.50 | 10.3 | 8.10 | 11.08 | 10.90 | 12 | 10 | 13 | 10 | 2 |
| *A. iranicus* | NEZMUT1377 | M | 55.72 | 27.76 | 14.42 | 9.16 | 6.88 | 6.98 | 8.60 | 4.28 | 9.62 | 9.18 | 12.96 | 13.04 | 14 | 10 | 13 | 10 | 2 |
| *A. iranicus* | NEZMUT1378 | F | 52.48 | 24.40 | 14.22 | 9.68 | 5.88 | 7.36 | 8.20 | 3.90 | 9.18 | 10.84 | 12.42 | 12.82 | 14 | 10 | 13 | 10 | 2 |
| *A. iranicus* | NEZMUT1379 | M | 55.79 | 26.46 | 15.10 | 10.70 | 6.44 | 6.78 | 8.44 | 3.70 | 10.16 | 8.38 | 12.89 | 12.94 | 13 | 11 | 14 | 11 | 2 |
| **Population 3** | NEZMUT1429 | F | 50.91 | 20.78 | 13.46 | 8.74 | 4.82 | 5.78 | 7.68 | 3.40 | 8.58 | 7.20 | 11.01 | 11.94 | 12 | 9 | 12 | 9 | 2 |
| **Population 3** | NEZMUT1430 | M | 53.22 | 22.36 | 14.98 | 9.08 | 5.66 | 6.80 | 8.50 | 3.88 | 10.80 | 10.10 | 11.70 | 12.88 | 11 | 8 | 12 | 9 | 2 |
| **Population 3** | NEZMUT1431 | M | 53.80 | 22.48 | 14.42 | 9.90 | 5.80 | 6.84 | 8.78 | 4.10 | 9.84 | 9.74 | 10.78 | 12.56 | 12 | 9 | 13 | 11 | 2 |
| **Population 3** | NEZMUT1432 | M | 54.08 | 22.16 | 13.58 | 9.58 | 5.76 | 7.14 | 8.34 | 3.92 | 9.80 | 10.14 | 10.98 | 12.44 | 11 | 8 | 13 | 10 | 2 |
| **Population 3** | NEZMUT1433 | F | 52.20 | 22.22 | 14.31 | 9.24 | 5.72 | 6.72 | 8.20 | 3.88 | 9.75 | 9.18 | 11.29 | 11.68 | 10 | 9 | 13 | 10 | 2 |
| *A. griseonotus* | NEZMUT1446 | F | 70.00 | 31.98 | 17.98 | 12.90 | 7.76 | 8.65 | 10.52 | 4.36 | 11.94 | 11.08 | 15.40 | 16.06 | 14 | 9 | 11 | 8 | 2 |
| *A. griseonotus* | NEZMUT1447 | F | 64.78 | 29.10 | 16.41 | 12.04 | 7.48 | 7.38 | 10.32 | 4.70 | 12.38 | 11.82 | 13.38 | 16.64 | 14 | 8 | 11 | 8 | 2 |
| *A. griseonotus* | NEZMUT1448 | M | 68.08 | 26.48 | 18.64 | 13.14 | 7.58 | 8.54 | 10.74 | 4.32 | 12.96 | 11.90 | 14.62 | 15.88 | 11 | 9 | 12 | 8 | 2 |
| *A. griseonotus* | NEZMUT1449 | F | 65.60 | 31.10 | 16.78 | 12.10 | 7.36 | 8.36 | 10.48 | 4.44 | 12.26 | 12.38 | 12.98 | 15.70 | 12 | 9 | 11 | 8 | 2 |
| *A. griseonotus* | NEZMUT1450 | F | 66.86 | 31.72 | 17.18 | 12.40 | 7.06 | 8.54 | 10.56 | 3.86 | 11.02 | 11.54 | 13.88 | 15.30 | 14 | 8 | 12 | 8 | 2 |
| *A. griseonotus* | NEZMUT1451 | M | 54.72 | 26.00 | 15.01 | 10.16 | 6.58 | 7.66 | 9.02 | 3.74 | 10.50 | 11.08 | 11.62 | 12.56 | 11 | 9 | 11 | 8 | 2 |
| *A. griseonotus* | NEZMUT1463 | F | 63.82 | 30.50 | 16.38 | 11.72 | 4.48 | 7.76 | 9.92 | 3.64 | 11.98 | 12.16 | 13.00 | 16.58 | 11 | 8 | 10 | 9 | 3 |
| *A. griseonotus* | NEZMUT1464 | F | 58.72 | 26.78 | 15.86 | 10.82 | 4.98 | 6.84 | 9.56 | 3.42 | 10.58 | 11.52 | 12.56 | 14.52 | 10 | 9 | 10 | 8 | 2 |
| *A. griseonotus* | NEZMUT1465 | M | 60.40 | 25.36 | 17.32 | 11.22 | 5.08 | 7.80 | 9.62 | 3.94 | 10.90 | 11.62 | 13.38 | 14.62 | 12 | 9 | 11 | 9 | 3 |
| *A. griseonotus* | NEZMUT1466 | F | 61.18 | 28.12 | 15.36 | 10.60 | 5.16 | 6.72 | 9.48 | 3.84 | 10.92 | 11.58 | 12.90 | 15.90 | 13 | 9 | 10 | 8 | 2 |
| *A. griseonotus* | NEZMUT1467 | F | 60.94 | 27.36 | 15.50 | 10.96 | 5.64 | 7.16 | 8.70 | 3.98 | 11.38 | 10.84 | 14.00 | 14.56 | 10 | 9 | 11 | 8 | 3 |
| *A. griseonotus* | NEZMUT1507 | F | 67.30 | 29.98 | 17.44 | 12.40 | 7.22 | 8.06 | 10.68 | 4.36 | 12.06 | 12.82 | 14.22 | 16.6 | 10 | 9 | 11 | 8 | 2 |
| *A. griseonotus* | NEZMUT1508 | M | 67.40 | 27.34 | 17.80 | 12.76 | 7.40 | 8.72 | 10.08 | 4.50 | 13.62 | 12.92 | 15.58 | 16.38 | 12 | 9 | 11 | 8 | 2 |
| *A. griseonotus* | NEZMUT1483 | F | 67.38 | 29.10 | 17.90 | 10.16 | 7.46 | 8.14 | 10.16 | 3.80 | 12.90 | 12.48 | 15.78 | 16.68 | 11 | 9 | 12 | 8 | 3 |
| *A. griseonotus* | NEZMUT1484 | F | 60.00 | 27.58 | 15.8 | 10.70 | 5.98 | 6.98 | 9.20 | 3.80 | 10.82 | 10.70 | 13.18 | 15.32 | 11 | 9 | 12 | 8 | 3 |
| *A. griseonotus* | NEZMUT1485 | M | 68.24 | 32.06 | 17.28 | 12.48 | 7.64 | 8.40 | 10.40 | 4.06 | 8.68 | 11.98 | 14.70 | 17.18 | 13 | 9 | 12 | 6 | 3 |
| *A. griseonotus* | NEZMUT1486 | F | 67.46 | 33.04 | 16.98 | 11.28 | 7.36 | 8.14 | 10.08 | 3.38 | 11.72 | 11.20 | 13.70 | 16.88 | 8 | 8 | 12 | 8 | 3 |
| *A. griseonotus* | NEZMUT1487 | F | 56.36 | 27.50 | 14.98 | 10.24 | 5.40 | 6.86 | 8.58 | 3.54 | 10.80 | 10.10 | 10.70 | 13.38 | 12 | 9 | 12 | 8 | 3 |
| *A. griseonotus* | NEZMUT1488 | F | 66.68 | 30.78 | 17.98 | 11.58 | 6.58 | 7.54 | 10.8 | 4.42 | 13.44 | 11.64 | 15.48 | 15.58 | 11 | 9 | 12 | 7 | 3 |
| *A. griseonotus* | NEZMUT1489 | F | 68.12 | 30.20 | 17.90 | 12.34 | 6.52 | 8.84 | 11.36 | 4.96 | 12.12 | 10.52 | 15.24 | 15.98 | 12 | 8 | 12 | 8 | 3 |
| *A. elisae* | NEZMUT1452 | M | 49.69 | 20.82 | 12.70 | 7.58 | 4.37 | 6.10 | 7.18 | 3.02 | 8.30 | 8.12 | 9.32 | 10.84 | 12 | 8 | 11 | 8 | 2 |
| *A. elisae* | NEZMUT1453 | M | 52.20 | 21.58 | 13.10 | 8.62 | 4.36 | 6.08 | 7.98 | 3.00 | 8.42 | 8.82 | 9.22 | 10.90 | 12 | 10 | 11 | 7 | 2 |
| *A. elisae* | NEZMUT1454 | F | 52.56 | 22.06 | 13.16 | 8.62 | 4.62 | 6.24 | 7.42 | 2.90 | 8.84 | 8.46 | 9.32 | 11.00 | 14 | 9 | 12 | 8 | 2 |
| *A. elisae* | NEZMUT1455 | M | 50.62 | 20.62 | 13.08 | 8.72 | 4.96 | 6.50 | 7.18 | 3.38 | 8.18 | 7.76 | 9.10 | 10.98 | 14 | 10 | 10 | 9 | 2 |
| *A. elisae* | NEZMUT1456 | F | 51.80 | 24.30 | 13.44 | 9.06 | 4.76 | 6.80 | 7.62 | 2.80 | 8.34 | 7.98 | 9.12 | 11.40 | 13 | 9 | 12 | 8 | 2 |
| *A. andersoni* | NEZMUT1418 | F | 64.26 | 28.36 | 16.41 | 10.95 | 6.04 | 7.50 | 8.99 | 3.86 | 10.90 | 11.28 | 13.84 | 15.28 | 15 | 10 | 13 | 8 | 2 |
| *A. andersoni* | NEZMUT1419 | M | 59.58 | 25.60 | 16.48 | 11.38 | 6.38 | 7.66 | 9.38 | 3.64 | 10.30 | 10.70 | 13.04 | 15.20 | 15 | 9 | 11 | 8 | 2 |
| *A. andersoni* | NEZMUT1420 | F | 62.94 | 28.99 | 16.38 | 11.48 | 6.38 | 7.76 | 9.56 | 3.42 | 10.82 | 11.22 | 13.6 | 14.26 | 16 | 10 | 11 | 8 | 2 |
| *A. andersoni* | NEZMUT1473 | M | 61.34 | 25.98 | 16.52 | 11.48 | 6.10 | 7.04 | 8.90 | 3.60 | 10.68 | 10.54 | 13.98 | 15.12 | 14 | 9 | 12 | 8 | 2 |
| *A. andersoni* | NEZMUT1474 | M | 64.92 | 28.28 | 16.98 | 12.00 | 6.32 | 7.66 | 9.96 | 3.54 | 11.60 | 11.28 | 14.64 | 16.44 | 15 | 8 | 12 | 8 | 2 |
| **Population 5** | NEZMUT1434 | M | 58.10 | 24.02 | 14.68 | 9.40 | 6.12 | 6.60 | 8.01 | 3.80 | 9.40 | 10.61 | 11.42 | 12.82 | 12 | 9 | 13 | 10 | 2 |
| **Population 5** | NEZMUT1435 | F | 51.78 | 23.59 | 13.36 | 8.78 | 4.98 | 6.68 | 7.40 | 3.40 | 8.82 | 8.22 | 10.98 | 11.84 | 13 | 10 | 12 | 9 | 2 |
| **Population 5** | NEZMUT1436 | F | 47.70 | 22.10 | 12.28 | 7.98 | 3.66 | 5.90 | 7.20 | 3.12 | 8.00 | 8.38 | 9.08 | 11.02 | 13 | 9 | 13 | 10 | 2 |
| **Population 5** | NEZMUT1437 | F | 49.22 | 22.56 | 12.82 | 8.20 | 3.92 | 6.16 | 6.94 | 3.18 | 7.10 | 7.56 | 9.02 | 10.54 | 13 | 9 | 10 | 9 | 2 |
| **Population 5** | NEZMUT1438 | F | 50.30 | 22.20 | 12.99 | 8.61 | 4.72 | 5.24 | 7.60 | 3.56 | 8.84 | 8.99 | 9.18 | 11.18 | 12 | 9 | 12 | 8 | 2 |
| **Population 5** | NEZMUT1439 | M | 52.08 | 22.46 | 13.02 | 9.34 | 4.86 | 6.32 | 7.38 | 3.18 | 9.62 | 8.99 | 9.68 | 12.50 | 13 | 9 | 11 | 9 | 2 |
| **Population 5** | NEZMUT1440 | M | 49.96 | 21.28 | 12.44 | 8.22 | 4.29 | 6.30 | 7.12 | 3.00 | 7.48 | 8.98 | 9.48 | 11.68 | 13 | 9 | 12 | 9 | 2 |
| **Population 2** | NEZMUT1468 | M | 58.34 | 26.10 | 15.35 | 10.68 | 5.84 | 7.26 | 9.48 | 3.44 | 10.38 | 11.58 | 13.24 | 14.62 | 14 | 10 | 13 | 10 | 2 |
| **Population 2** | NEZMUT1469 | M | 63.26 | 26.36 | 16.56 | 12.20 | 6.50 | 7.65 | 9.54 | 3.74 | 12.21 | 11.68 | 13.99 | 16.04 | 14 | 9 | 13 | 8 | 2 |
| **Population 2** | NEZMUT1470 | F | 53.18 | 24.64 | 14.48 | 9.38 | 6.12 | 6.28 | 8.74 | 3.08 | 9.38 | 8.60 | 11.08 | 11.84 | 14 | 9 | 12 | 9 | 4 |
| **Population 4** | NEZMUT1471 | J | 35.58 | 15.60 | 9.48 | 6.10 | 3.70 | 4.38 | 5.12 | 2.34 | 5.40 | 4.48 | 7.86 | 6.94 | 12 | 9 | 10 | 8 | 2 |
| **Population 4** | NEZMUT1472 | M | 49.02 | 21.42 | 12.45 | 7.97 | 4.50 | 5.60 | 7.64 | 3.24 | 8.66 | 8.44 | 9.41 | 12.16 | 13 | 8 | 9 | 8 | 2 |
| *A. kermanshahensis* | NEZMUT1350 | F | 49.72 | 25.8 | 13.24 | 9.12 | 4.54 | 5.08 | 8.24 | 3.54 | 9.24 | 7.54 | 8.91 | 11.34 | 12 | 7 | 13 | 10 | 4 |
| *A. kermanshahensis* | NEZMUT1351 | M | 54.22 | 23.90 | 14.64 | 9.28 | 4.28 | 6.38 | 8.16 | 3.48 | 8.18 | 8.92 | 10.43 | 12.32 | 11 | 9 | 12 | 9 | 4 |
| *A. kermanshahensis* | NEZMUT1475 | F | 54.82 | 27.01 | 14.60 | 9.52 | 5.06 | 6.48 | 8.60 | 3.24 | 8.96 | 9.72 | 9.90 | 12.72 | 12 | 9 | 12 | 9 | 4 |
| *A. kermanshahensis* | NEZMUT1476 | J | 41.48 | 20.82 | 11.06 | 7.42 | 3.84 | 4.64 | 5.58 | 2.80 | 6.40 | 6.98 | 7.56 | 9.02 | 10 | 8 | 12 | 10 | 4 |
| *A. kermanshahensis* | NEZMUT1477 | F | 53.54 | 28.76 | 14.00 | 9.11 | 5.20 | 6.21 | 8.03 | 3.18 | 9.22 | 9.40 | 10.68 | 12.62 | 11 | 8 | 12 | 10 | 4 |
| *A. kermanshahensis* | NEZMUT1478 | M | 59.20 | 28.08 | 15.12 | 11.18 | 5.48 | 7.98 | 8.86 | 3.22 | 8.64 | 9.90 | 11.28 | 13.46 | 14 | 9 | 12 | 11 | 4 |
| *A. kermanshahensis* | NEZMUT1479 | J | 41.22 | 20.78 | 11.18 | 7.56 | 3.74 | 5.40 | 6.74 | 2.66 | 7.28 | 7.38 | 8.74 | 10.10 | 12 | 8 | 13 | 10 | 4 |
| *A. kermanshahensis* | NEZMUT1480 | F | 53.98 | 25.92 | 14.20 | 9.31 | 5.38 | 6.36 | 7.70 | 3.18 | 8.26 | 8.61 | 10.16 | 12.82 | 12 | 8 | 13 | 9 | 4 |
| *A. gardneri* | BMNH1976.1416 | M | 60.4 | 25.6 | 17.9 | 11.1 | 7.1 | 7.0 | 9.3 | 5.2 | 11.5 | 9.2 | 14.7 | 16.0 | 14 | 9 | 13 | 10 | 2 |
| *A. gardneri* | BMNH1976.1417 | M | 59.6 | 25.0 | 18.4 | 10.7 | 7.1 | 7.3 | 9.0 | 5.1 | 11.4 | 9.1 | 14.6 | 15.7 | 14 | 9 | 14 | 10 | 2 |
| *A. gardneri* | BMNH1976.1418 | M | 56.1 | 22.4 | 17.3 | 10.5 | 6.4 | 6.7 | 8.8 | 5.1 | 9.8 | 8.8 | 12.4 | 13.5 | 14 | 10 | 14 | 10 | 2 |
| *A. gardneri* | BMNH2008.1000* | M | 63.4 | 29.3 | 18.3 | 13.2 | 8.8 | 7.0 | 9.5 | 4.7 | 11.6 | 9.1 | 14.0 | 14.7 | 15 | 10 | 14 | 10 | 2 |
| *A. gardneri* | IBECN3907 | M | 66.8 | 26.1 | 19.2 | 13.3 | 9.1 | 7.8 | 10.8 | 5.1 | 13.0 | 10.0 | 15.9 | 15.4 | 15 | 11 | 15 | 11 | 2 |
| *A. gardneri* | IBECN3910 | M | 60.9 | 25.0 | 17.9 | 11.1 | 6.6 | 8.8 | 10.1 | 4.5 | 11.8 | 9.5 | 14.0 | 15.6 | 13 | 10 | 14 | 11 | 2 |
| *A. gardneri* | IBECN3955 | M | 68.4 | 28.2 | 20.0 | 13.5 | 8.8 | 9.3 | 11.0 | 4.8 | 13.7 | 9.7 | 15.8 | 17.1 | 14 | 9 | 15 | 12 | 2 |
| *A. gardneri* | IBECN751 | M | 67.0 | 27.2 | 19.8 | 13.8 | 8.3 | 7.2 | 10.6 | 5.2 | 12.8 | 9.6 | 14.8 | 16.6 | 14 | 10 | 12 | 9 | 2 |
| *A. gardneri* | IBECN757 | M | 66.8 | 27.0 | 19.6 | 12.0 | 7.5 | 9.2 | 10.3 | 5.4 | 11.6 | 9.2 | 14.9 | 15.3 | 14 | 10 | 12 | 9 | 2 |
| *A. gardneri* | IBECN784 | M | 64.7 | 25.8 | 19.5 | 12.4 | 7.9 | 7.8 | 10.9 | 5.8 | 12.3 | 10.0 | 15.0 | 16.7 | 14 | 10 | 14 | 10 | 2 |
| *A. gardneri* | IBECN797 | M | 68.6 | 28.3 | 19.9 | 14.1 | 8.4 | 7.1 | 10.3 | 5.5 | 13.7 | 9.7 | 16.1 | 16.8 | 12 | 11 | 14 | 11 | 2 |
| *A. gardneri* | IBECN842 | M | 63.9 | 28.6 | 19.0 | 12.1 | 7.8 | 8.1 | 10.3 | 5.0 | 13.6 | 9.7 | 15.8 | 16.4 | 13 | 10 | 14 | 11 | 2 |
| *A. gardneri* | IBECN848 | M | 69.8 | 28.1 | 20.9 | 12.6 | 8.0 | 9.1 | 10.9 | 5.8 | 12.9 | 11.0 | 16.1 | 16.5 | 14 | 9 | 16 | 11 | 2 |
| *A. gardneri* | IBECN9008 | M | 59.0 | 23.7 | 17.7 | 10.8 | 6.4 | 7.4 | 9.6 | 5.1 | 10.9 | 9.6 | 13.8 | 14.0 | 14 | 9 | 14 | 10 | 2 |
| *A. gardneri* | SQU1988.48 | M | 70.7 | 29.8 | 20.7 | 12.9 | 8.1 | 8.1 | 9.9 | 5.5 | 13.8 | 12.2 | 16.8 | 18.0 | 14 | 9 | 13 | 11 | 2 |
| *A. gardneri* | IBECN10426 | M | 62.0 | 26.7 | 18.7 | 12.0 | 7.3 | 6.6 | 10.0 | 4.6 | 11.9 | 10.0 | 15.0 | 17.0 | 15 | 9 | 14 | 11 | 2 |
| *A. gardneri* | IBECN10427 | M | 63.7 | 26.4 | 19.7 | 12.1 | 7.8 | 7.8 | 10.5 | 4.7 | 12.2 | 9.9 | 15.5 | 16.4 | 14 | 9 | 13 | 10 | 2 |
| *A. gardneri* | BMNH1976.1414 | F | 62.0 | 24.2 | 18.4 | 11.9 | 6.9 | 7.6 | 9.9 | 5.5 | 11.1 | 9.2 | 14.7 | 15.5 | 14 | 9 | 14 | 9 | 2 |
| *A. gardneri* | BMNH1976.1415 | F | 63.6 | 24.5 | 19.3 | 12.0 | 7.1 | 7.8 | 9.6 | 5.0 | 11.9 | 9.3 | 14.8 | 16.4 | 15 | 9 | 15 | 12 | 2 |
| *A. gardneri* | BMNH1976.1419 | F | 54.0 | 20.9 | 16.6 | 10.1 | 6.4 | 6.6 | 8.3 | 4.6 | 10.3 | 8.5 | 13.8 | 14.1 | 15 | 10 | 14 | 10 | 2 |
| *A. gardneri* | ONHM4221 | F | 64.0 | 26.5 | 19.0 | 11.7 | 7.6 | 8.2 | 10.3 | 5.1 | 12.0 | 9.1 | 14.6 | 16.5 | 14 | 10 | 14 | 10 | 2 |
| *A. gardneri* | IBECN3042 | F | 62.9 | 27.0 | 19.0 | 11.7 | 7.1 | 8.1 | 10.0 | 5.0 | 11.5 | 9.5 | 14.0 | 14.9 | 14 | 9 | 13 | 9 | 2 |
| *A. gardneri* | IBECN3901 | F | 57.9 | 22.7 | 17.4 | 10.7 | 6.4 | 7.1 | 9.1 | 5.4 | 11.2 | 9.7 | 14.9 | 15.2 | 14 | 10 | 15 | 10 | 2 |
| *A. gardneri* | IBECN3903 | F | 51.8 | 19.8 | 16.1 | 10.2 | 6.3 | 6.4 | 8.3 | 5.0 | 10.1 | 8.4 | 12.9 | 12.6 | 14 | 10 | 13 | 10 | 2 |
| *A. gardneri* | IBECN3904 | F | 70.7 | 30.0 | 20.6 | 13.4 | 9.0 | 9.0 | 11.4 | 5.2 | 14.3 | 11.1 | 17.4 | 17.8 | 15 | 10 | 14 | 11 | 2 |
| *A. gardneri* | IBECN3914 | F | 61.5 | 24.9 | 18.4 | 11.8 | 7.6 | 7.7 | 9.8 | 4.9 | 11.4 | 9.5 | 13.9 | 14.2 | 14 | 9 | 12 | 9 | 2 |
| *A. gardneri* | IBECN3965 | F | 60.7 | 25.1 | 18.3 | 11.4 | 8.0 | 7.4 | 9.8 | 5.0 | 11.9 | 9.7 | 14.5 | 16.3 | 13 | 9 | 14 | 10 | 2 |
| *A. gardneri* | BMNH2008.999 | F | 61.6 | 26.0 | 18.1 | 11.2 | 7.4 | 7.6 | 10.1 | 5.0 | 12.3 | 8.6 | 13.9 | 15.5 | 14 | 9 | 15 | 10 | 2 |
| *A. gardneri* | IBECN760 | F | 67.4 | 28.0 | 19.6 | 13.2 | 8.4 | 7.4 | 10.7 | 5.3 | 13.5 | 10.7 | 16.4 | 16.7 | 14 | 10 | 12 | 10 | 2 |
| *A. gardneri* | IBECN801 | F | 69.2 | 30.0 | 21.0 | 13.7 | 7.4 | 8.9 | 11.4 | 5.0 | 13.4 | 9.7 | 16.2 | 16.8 | 14 | 10 | 13 | 11 | 2 |
| *A. gardneri* | IBECN8109 | F | 56.8 | 21.1 | 17.4 | 9.6 | 6.9 | 7.3 | 8.3 | 4.6 | 10.4 | 8.7 | 14.2 | 13.8 | 11 | 9 | 14 | 11 | 2 |
| *A. gardneri* | IBECN817 | F | 54.8 | 22.7 | 16.5 | 10.7 | 6.3 | 6.9 | 9.3 | 4.8 | 10.6 | 9.1 | 13.5 | 13.3 | 16 | 10 | 14 | 10 | 2 |
| *A. gardneri* | IBECN8370 | F | 59.9 | 24.4 | 18.0 | 10.3 | 6.4 | 8.1 | 9.5 | 4.8 | 11.0 | 9.4 | 12.9 | 14.8 | 13 | 9 | 14 | 10 | 2 |
| *A. gardneri* | IBECN844 | F | 61.5 | 25.6 | 18.7 | 12.6 | 7.5 | 7.6 | 9.8 | 5.2 | 12.0 | 9.0 | 13.5 | 16.1 | 13 | 9 | 13 | 9 | 2 |
| *A. gardneri* | IBECN8673 | F | 59.5 | 22.5 | 18.0 | 12.0 | 8.2 | 7.8 | 9.9 | 4.5 | 12.2 | 9.7 | 14.4 | 14.5 | 13 | 9 | 14 | 11 | 2 |
| *A. gardneri* | IBECN8674 | F | 57.6 | 21.5 | 17.5 | 11.1 | 6.2 | 7.3 | 9.5 | 4.8 | 10.8 | 10.1 | 13.4 | 15.0 | 14 | 11 | 15 | 11 | 2 |
| *A. gardneri* | IBECN8700 | F | 52.8 | 20.8 | 15.7 | 9.9 | 5.8 | 6.7 | 8.7 | 4.6 | 10.9 | 9.3 | 13.2 | 13.5 | 12 | 10 | 14 | 10 | 2 |
| *A. gardneri* | IBECN8715 | F | 65.4 | 29.5 | 19.0 | 12.1 | 8.5 | 7.6 | 10.0 | 5.1 | 12.2 | 10.4 | 15.8 | 16.4 | 15 | 11 | 15 | 11 | 2 |
| *A. gardneri* | SQU1988.49 | F | 62.0 | 25.6 | 17.6 | 11.0 | 7.5 | 7.6 | 9.3 | 5.3 | 11.8 | 10.2 | 14.9 | 16.6 | 15 | 10 | 13 | 11 | 2 |
| *A. gardneri* | IBECN10423 | F | 65.4 | 27.4 | 20.7 | 13.6 | 7.2 | 7.8 | 10.5 | 5.4 | 12.6 | 10.0 | 15.5 | 15.9 | 15 | 10 | 14 | 10 | 2 |
| *A. gardneri* | IBECN10424 | F | 60.6 | 26.7 | 18.1 | 12.1 | 7.4 | 7.4 | 10.3 | 4.8 | 11.1 | 9.1 | 13.7 | 16.2 | 15 | 10 | 12 | 9 | 2 |
| *A. gardneri* | IBECN10428 | F | 59.9 | 22.6 | 18.7 | 11.1 | 7.1 | 7.3 | 9.4 | 4.8 | 11.2 | 8.9 | 13.7 | 14.5 | 15 | 9 | 14 | 10 | 2 |
| *A. gardneri* | IBECN10425 | F | 63.3 | 24.5 | 19.3 | 11.9 | 8.0 | 8.3 | 10.5 | 5.2 | 12.6 | 11.0 | 15.1 | 15.8 | 14 | 10 | 13 | 9 | 2 |
| *A. caudivolvulus* | BMNH1973.1850 | M | 63.2 | 26.5 | 18.7 | 13.0 | 7.3 | 7.2 | 10.1 | 5.0 | 10.0 | 9.2 | 14.0 | 15.7 | 15 | 10 | 13 | 11 | 2 |
| *A. caudivolvulus* | BMNH1973.18951 | M | 62.9 | 27.7 | 18.6 | 11.6 | 7.0 | 7.0 | 9.3 | 5.4 | 10.8 | 9.3 | 13.8 | 15.6 | 16 | 9 | 15 | 11 | 2 |
| *A. caudivolvulus* | IBES7445 | M | 61.3 | 25.7 | 18.9 | 12.0 | 7.4 | 7.7 | 10.3 | 4.8 | 10.8 | 8.9 | 12.5 | 14.1 | 14 | 9 | 14 | 9 | 2 |
| *A. caudivolvulus* | IBES8088 | M | 53.6 | 21.9 | 15.9 | 9.9 | 6.2 | 6.7 | 8.7 | 4.3 | 9.3 | 7.9 | 12.0 | 13.0 | 16 | 9 | 13 | 9 | 2 |
| *A. caudivolvulus* | IBES7866 | F | 58.5 | 23.9 | 17.7 | 11.6 | 6.4 | 8.2 | 9.9 | 4.9 | 10.0 | 8.7 | 12.3 | 13.1 | 16 | 8 | 13 | 9 | 2 |
| *A. margaritae* | IBECN2997 | M | 58.6 | 26.1 | 17.1 | 11.8 | 6.2 | 7.7 | 9.5 | 3.6 | 9.3 | 7.7 | 11.2 | 12.5 | 14 | 9 | 13 | 10 | 2 |
| *A. margaritae* | BMNH2008.989* | M | 54.8 | 23.4 | 15.8 | 11.2 | 6.2 | 7.0 | 8.9 | 4.2 | 8.9 | 7.8 | 10.6 | 12.2 | 14 | 9 | 14 | 10 | 2 |
| *A. margaritae* | IBECN8191 | M | 51.7 | 21.6 | 15.2 | 10.5 | 4.8 | 6.3 | 8.7 | 3.8 | 8.1 | 6.7 | 10.2 | 10.5 | 13 | 8 | 14 | 10 | 2 |
| *A. margaritae* | IBECN8708 | M | 56.0 | 24.5 | 16.2 | 10.5 | 5.9 | 6.9 | 9.0 | 4.2 | 8.5 | 7.9 | 10.8 | 12.0 | 13 | 7 | 13 | 10 | 2 |
| *A. margaritae* | IBECN9012 | M | 53.7 | 23.4 | 15.5 | 10.1 | 6.1 | 6.9 | 9.0 | 3.9 | 8.9 | 7.6 | 10.5 | 11.5 | 16 | 8 | 14 | 11 | 2 |
| *A. margaritae* | IBECN9020 | M | 53.8 | 22.5 | 15.9 | 10.6 | 6.1 | 6.6 | 8.8 | 4.1 | 8.5 | 7.2 | 10.4 | 12.0 | 13 | 8 | 13 | 10 | 2 |
| *A. margaritae* | IBECN9023 | M | 56.3 | 22.9 | 15.9 | 10.2 | 5.6 | 7.1 | 8.9 | 4.4 | 8.6 | 8.2 | 10.4 | 12.0 | 12 | 8 | 14 | 10 | 2 |
| *A. margaritae* | IBECN10419 | M | 53.9 | 18.9 | 15.9 | 11.5 | 6.8 | 6.4 | 8.8 | 3.8 | 8.3 | 7.2 | 10.3 | 12.8 | 16 | 8 | 13 | 9 | 2 |
| *A. margaritae* | IBECN10420 | M | 55.7 | 22.6 | 16.7 | 11.7 | 7.6 | 7.2 | 9.4 | 3.9 | 8.9 | 7.2 | 10.9 | 12.0 | 14 | 8 | 13 | 10 | 2 |
| *A. margaritae* | IBECN10421 | M | 52.9 | 22.3 | 16.4 | 11.4 | 7.0 | 6.5 | 9.0 | 4.0 | 8.0 | 7.3 | 9.0 | 13.1 | 15 | 8 | 12 | 9 | 2 |
| *A. margaritae* | ONHM4222 | F | 54.7 | 22.9 | 16.0 | 10.5 | 5.7 | 7.3 | 9.0 | 4.4 | 8.2 | 6.6 | 10.6 | 10.4 | 14 | 9 | 14 | 10 | 2 |
| *A. margaritae* | BMNH2008.988 | F | 44.3 | 17.5 | 12.7 | 9.1 | 5.4 | 5.2 | 7.3 | 3.2 | 6.9 | 6.1 | 8.4 | 9.6 | 12 | 9 | 14 | 11 | 2 |
| *A. margaritae* | IBECN10422 | F | 53.2 | 20.4 | 15.6 | 11.1 | 6.8 | 7.1 | 9.3 | 3.9 | 7.6 | 7.0 | 11.3 | 11.9 | 14 | 8 | 12 | 10 | 2 |
| *A. arnoldi* | IBES7224 | M | 33.6 | 13.2 | 8.7 | 5.2 | 3.2 | 3.9 | 2.8 | 1.9 | 5.1 | 5.2 | 6.8 | 7 |  |  |  | 12 | 2 |
| *A. arnoldi* | BMNH2008.961 | M | 31.4 | 11.8 | 8.1 | 5.3 | 3 | 3.7 | 2.9 | 1.9 | 4.9 | 4.4 | 6.3 | 6.4 |  |  |  | 11 | 2 |
| *A. arnoldi* | IBES7576 | M | 30.8 | 11.3 | 8.3 | 5.4 | 3.1 | 3.6 | 2.7 | 2 | 4.9 | 4.3 | 6 | 6.2 |  |  |  | 10 | 2 |
| *A. arnoldi* | IBECN3190 | F | 29.3 | 12.1 | 7.7 | 4.9 | 2.8 | 3.7 | 2.5 | 1.9 | 4.3 | 4.6 | 5.9 | 6.3 |  |  |  | 10 | 2 |
| *A. arnoldi* | ONHM4234 | F | 28.9 | 11.4 | 7.5 | 4.9 | 2.7 | 3.4 | 2.6 | 1.8 | 4.6 | 3.9 | 5.2 | 6.2 |  |  |  | 10 | 2 |
| *A. arnoldi* | BMNH2008.962 | F | 33.3 | 12 | 8.4 | 5.3 | 3.1 | 4 | 2.9 | 1.9 | 4.7 | 4.9 | 6.2 | 6.8 |  |  |  | 12 | 2 |
| *A. arnoldi* | IBECN416 | F | 31.8 | 11.9 | 8.3 | 5.1 | 3.1 | 3.8 | 2.7 | 2 | 4.6 | 5 | 6.1 | 7 |  |  |  | 11 | 2 |
| *A. arnoldi* | IBECN4310 | F | 29.4 | 11.5 | 7.4 | 4.7 | 2.9 | 3.4 | 2.6 | 1.7 | 4.6 | 3.9 | 5.5 | 6.1 |  |  |  | 10 | 2 |
| *A. arnoldi* | IBECN4356 | F | 32.4 | 13.3 | 8.5 | 5.1 | 3.1 | 3.7 | 2.8 | 2.1 | 4.7 | 4.3 | 6 | 6.4 |  |  |  | 10 | 2 |
| *A. arnoldi* | IBECN182 | F | 29.9 | 12.6 | 7.9 | 5.1 | 2.8 | 3.4 | 2.8 | 1.8 | 4.5 | 4.3 | 5.9 | 6.6 |  |  |  | 11 | 2 |
| *A. arnoldi* | IBECN4013 | M | 28.6 | 11.3 | 7.6 | 5 | 2.8 | 3.2 | 2.3 | 1.6 | 4.2 | 4.2 | 5.3 | 6 |  |  |  | 10 | 2 |
| *A. arnoldi* | IBECN4043 | M | 27.4 | 10.5 | 7.1 | 4.6 | 2.6 | 3.2 | 2.4 | 1.7 | 4.1 | 3.9 | 5.4 | 5.6 |  |  |  | 10 | 2 |
| *A. arnoldi* | IBECN4264 | M | 27.4 | 10.9 | 7.4 | 4.8 | 2.7 | 3.4 | 2.3 | 1.7 | 4.2 | 4.2 | 5.3 | 5.7 |  |  |  | 11 | 2 |
| *A. gallagheri* | IBES7734 | M | 35.3 | 15.1 | 9.2 | 5.8 | 3.4 | 4.1 | 3 | 2.3 | 5.4 | 4.5 | 7.3 | 6.5 |  |  |  | 12 | 2 |
| *A. gallagheri* | IBECN118 | M | 32.8 | 12.5 | 8.5 | 5.7 | 3.3 | 3.7 | 2.7 | 2 | 5 | 4.7 | 6.3 | 7 |  |  |  | 12 | 2 |
| *A. gallagheri* | IBECN195 | M | 33.1 | 13.3 | 8.6 | 5.8 | 3.4 | 4 | 3 | 2.1 | 5.1 | 5.2 | 6.5 | 7.4 |  |  |  | 11 | 2 |
| *A. gallagheri* | IBECN2598 | M | 30 | 11.3 | 8 | 4.6 | 3.1 | 3.7 | 2.6 | 1.8 | 4.6 | 4.5 | 5.6 | 6.4 |  |  |  | 11 | 2 |
| *A. gallagheri* | IBECN2606 | M | 35.9 | 13.8 | 9.7 | 6 | 3.8 | 4.4 | 3.2 | 2.2 | 5.5 | 6 | 7.7 | 8.5 |  |  |  | 12 | 2 |
| *A. gallagheri* | IBECN2790 | M | 35.7 | 15.1 | 9.4 | 6.1 | 3.3 | 4.2 | 3 | 2.2 | 5.4 | 4.7 | 7.1 | 6.8 |  |  |  | 12 | 2 |
| *A. gallagheri* | IBECN2803 | M | 36.4 | 14.4 | 9.4 | 6.2 | 3.7 | 4.1 | 3.1 | 2.2 | 5.6 | 5.8 | 7.7 | 7.9 |  |  |  | 11 | 2 |
| *A. gallagheri* | IBECN2817 | M | 32.9 | 13.6 | 8.6 | 5.2 | 3.4 | 3.9 | 2.8 | 2.1 | 4.9 | 5 | 6.3 | 7.6 |  |  |  | 10 | 2 |
| *A. gallagheri* | IBECN3739 | M | 37.2 | 15.4 | 9.5 | 6 | 3.8 | 4.4 | 3.3 | 2.5 | 5.3 | 5.5 | 7.8 | 8.3 |  |  |  | 10 | 2 |
| *A. gallagheri* | IBECN4179 | M | 33.6 | 13 | 8.5 | 6 | 3.5 | 4 | 2.9 | 2 | 5.1 | 4.8 | 7 | 7.2 |  |  |  | 11 | 2 |
| *A. gallagheri* | IBECN5815 | M | 32.7 | 13.1 | 8.5 | 5.2 | 3.1 | 3.7 | 2.7 | 2.2 | 4.7 | 4.4 | 6.4 | 6.6 |  |  |  | 11 | 2 |
| *A. gallagheri* | IBECN5817 | M | 32.7 | 13.6 | 8.6 | 5.4 | 3.2 | 3.7 | 2.6 | 2.1 | 4.9 | 4.9 | 6.1 | 6.3 |  |  |  | 12 | 2 |
| *A. gallagheri* | IBECN89 | M | 31.4 | 12.8 | 8.2 | 5.3 | 3.1 | 3.7 | 2.6 | 2.1 | 4.5 | 5 | 6.7 | 6.9 |  |  |  | 12 | 2 |
| *A. gallagheri* | IBES7662 | M | 32 | 12.7 | 8.8 | 5.7 | 3.7 | 3.8 | 2.9 | 2.3 | 4.7 | 4.3 | 6.1 | 6 |  |  |  | 12 | 2 |
| *A. gallagheri* | IBECN2613 | F | 37.3 | 15.3 | 9.1 | 5.7 | 3.7 | 4.1 | 3.1 | 2.2 | 5.2 | 5.5 | 7.4 | 8.1 |  |  |  | 11 | 2 |
| *A. gallagheri* | IBECN204 | F | 36 | 15.4 | 8.8 | 5.7 | 3.3 | 3.7 | 2.9 | 2.1 | 4.9 | 5.1 | 6.5 | 7.7 |  |  |  | 13 | 2 |
| *A. gallagheri* | IBECN217 | F | 36.2 | 15.9 | 9.2 | 5.9 | 3.6 | 4.1 | 3 | 2.4 | 5.1 | 5.2 | 6.9 | 7.7 |  |  |  | 12 | 2 |
| *A. gallagheri* | IBECN2800 | F | 28.3 | 11.2 | 8 | 4.8 | 2.9 | 3.6 | 2.7 | 1.9 | 4.1 | 4.4 | 5.4 | 6.1 |  |  |  | 11 | 2 |
| *A. gallagheri* | IBECN3484 | F | 32.7 | 14.3 | 8.6 | 5.3 | 3.2 | 3.8 | 2.6 | 2.3 | 4.9 | 4.9 | 6.6 | 7.2 |  |  |  | 13 | 2 |
| *A. gallagheri* | IBECN4011 | F | 34.9 | 13 | 9.2 | 5.9 | 3.3 | 4.2 | 2.7 | 2.4 | 5.3 | 4.8 | 6.5 | 7.9 |  |  |  | 12 | 2 |
| *A. gallagheri* | IBECN4024 | F | 34.2 | 13.7 | 8.9 | 5.7 | 3.5 | 4 | 2.9 | 2.2 | 5.5 | 5.5 | 6.5 | 7 |  |  |  | 12 | 2 |
| *A. gallagheri* | IBECN4167 | F | 30.9 | 12.7 | 8.4 | 5.4 | 3.1 | 3.8 | 2.8 | 2 | 4.8 | 4.3 | 6 | 6.8 |  |  |  | 12 | 2 |
| *A. gallagheri* | IBECN4313 | F | 33.8 | 12.5 | 8.8 | 5.6 | 3.5 | 4.1 | 2.8 | 2 | 5.1 | 4.2 | 6.4 | 7 |  |  |  | 12 | 2 |
| *A. gallagheri* | IBECN7074 | F | 33.7 | 14.3 | 8.6 | 5.7 | 3.2 | 3.5 | 2.6 | 2.1 | 4.8 | 4.6 | 6.1 | 6.2 |  |  |  | 12 | 2 |
| *A. gallagheri* | IBECN7945 | F | 33.2 | 14.5 | 8.4 | 5.7 | 2.9 | 3.9 | 2.6 | 2 | 4.9 | 4.8 | 6.4 | 6.7 |  |  |  | 11 | 2 |
| *A. gallagheri* | IBECN8040 | F | 29.7 | 11.7 | 7.9 | 4.8 | 3.1 | 3.6 | 2.2 | 1.8 | 4.7 | 4.5 | 5.5 | 6.5 |  |  |  | 11 | 2 |
| *A. gallagheri* | IBECN8294 | F | 32 | 12.5 | 8.2 | 4.9 | 3.3 | 3.9 | 2.5 | 1.8 | 4.6 | 4.7 | 6.1 | 6.3 |  |  |  | 12 | 2 |
| *A. gallagheri* | IBECN96 | F | 35.5 | 14.4 | 8.9 | 5.9 | 3.3 | 3.8 | 2.9 | 2.2 | 4.8 | 5.4 | 6.5 | 7 |  |  |  | 12 | 2 |
| *A. gallagheri* | IBECN3524 | F | 35.5 | 14.3 | 9.1 | 5.6 | 3.4 | 4.2 | 2.8 | 2.3 | 5.3 | 5.1 | 7.5 | 7.5 |  |  |  | 13 | 2 |
| *A. gallagheri* | IBECN3728 | F | 33.9 | 14.1 | 8.9 | 5.5 | 3.4 | 4 | 2.5 | 2.2 | 4.6 | 4.1 | 6.6 | 6.7 |  |  |  | 11 | 2 |
| *A. gallagheri* | IBECN7106 | F | 26.5 | 9.8 | 7.3 | 4.9 | 2.8 | 3.4 | 2.2 | 1.8 | 3.7 | 3.6 | 5.3 | 5.5 |  |  |  | 12 | 2 |
| *A. gallagheri* | IBECN8036 | F | 29 | 11.7 | 7.9 | 5 | 2.9 | 3.2 | 2.5 | 2 | 4.2 | 4.1 | 5.3 | 6.2 |  |  |  | 11 | 2 |
| *A. gallagheri* | IBECN5844 | F | 27.2 | 11.9 | 6.9 | 4.4 | 2.6 | 3.1 | 2.1 | 1.6 | 4 | 3.6 | 4.8 | 5 |  |  |  | 13 | 2 |
| *A. gallagheri* | TW1037 | F | 30.9 | 13 | 8 | 5.7 | 3.2 | 3.7 | 2.7 | 2.1 | 4.4 | 4.5 | 5.8 | 6.5 |  |  |  | 11 | 2 |
| *A. gallagheri* | TW1038 | F | 37.3 | 15.5 | 9.4 | 6.1 | 3.6 | 4.2 | 3.2 | 2.4 | 5.2 | 5.6 | 6.9 | 8.2 |  |  |  | 11 | 2 |
| *A. gallagheri* | TW1039 | F | 36.2 | 16.8 | 9 | 6.1 | 3.6 | 4.1 | 3.1 | 2.2 | 5.2 | 5.5 | 6.3 | 7.7 |  |  |  | 11 | 2 |
| *A. gallagheri* | TW1026 | F | 33.9 | 14.3 | 8.6 | 5.8 | 3.3 | 3.9 | 2.8 | 2.2 | 4.7 | 4.9 | 6.3 | 7.3 |  |  |  | 11 | 2 |
| *A. gallagheri* | TW1027 | F | 32.3 | 13.1 | 8.2 | 5.3 | 3.1 | 3.5 | 2.4 | 2.2 | 4.9 | 4.7 | 6.1 | 6.5 |  |  |  | 10 | 2 |
| *A. gallagheri* | IBES6034 | F | 34.5 | 13.1 | 9.7 | 6.1 | 3.4 | 4.4 | 2.9 | 2.4 | 5.4 | 4.5 | 7.1 | 7.2 |  |  |  | 11 | 2 |
| *A. gallagheri* | IBECN2593 | F | 33.4 | 13.4 | 8.7 | 5.3 | 3.5 | 4 | 2.5 | 2.1 | 4.9 | 4.3 | 6.9 | 6.7 |  |  |  | 12 | 2 |
| *A. gallagheri* | IBECN14 | M | 31.4 | 11.5 | 8.3 | 5.2 | 3.4 | 3.7 | 2.5 | 2 | 4.7 | 4.2 | 6.4 | 6.3 |  |  |  | 10 | 2 |
