# Supplementary material for: Integrating phylogenetic, phylogeographic, and morphometric analyses to reveal cryptic lineages within the genus Asaccus (Reptilia: Squamata: Phyllodactylidae) in Iran
Source: BMC Zool. 2024 Jun 26;9:12. doi: 10.1186/s40850-024-00203-1 (PMC11202258; doi:10.1186/s40850-024-00203-1)
Supplement: Supplementary file 5 — Supplementary Material 5 [file 40850_2024_203_MOESM5_ESM.docx]

| PC | Eigenvalue | Variability (%) | Cumulative (%) |
| --- | --- | --- | --- |
| **1** | 10.4136 | 94.669 | 96.669 |
| **2** | 0.31203 | 2.8367 | 97.5057 |
| **3** | 0.12954 | 1.1776 | 98.6833 |
| **4** | 0.06930 | 0.6300 | 99.3133 |
| **5** | 0.03413 | 0.3103 | 99.6236 |
| **6** | 0.02164 | 0.1967 | 99.8203 |

**Table S5.** Eigenvalues, proportion and cumulative proportion of variance explained of the first six components selected from the PCA performed on shape residuals for each morphological variable
